# Sociodemographic and clinical predictors of adherence to antidepressants in depressive disorders: a systematic review with a meta-analysis

**DOI:** 10.3389/fphar.2024.1327155

**Published:** 2024-01-22

**Authors:** Tasmania Del Pino-Sedeño, Diego Infante-Ventura, Diego Hernández-González, Yadira González-Hernández, Beatriz González de León, Amado Rivero-Santana, Isabel Hurtado, Francisco Javier Acosta Artiles

**Affiliations:** ^1^ Canary Islands Health Research Institute Foundation (FIISC), Santa Cruz de Tenerife, Spain; ^2^ Evaluation Unit (SESCS), Canary Islands Health Service (SCS), Santa Cruz de Tenerife, Spain; ^3^ Research Network on Chronicity, Primary Care and Prevention and Health Promotion (RICAPPS), Tenerife, Spain; ^4^ Faculty of Health Sciences, Universidad Europea de Canarias, Tenerife, Spain; ^5^ Department of Clinical Psychology, Psychobiology and Methodology, University of La Laguna, Tenerife, Spain; ^6^ Multiprofessional Teaching Unit of Family and Community Care La Laguna-Tenerife Norte, Management of Primary Care of Tenerife, Santa Cruz de Tenerife, Spain; ^7^ The Foundation for the Promotion of Health and Biomedical Research of Valencia Region (FISABIO), Valencia, Spain; ^8^ Department of Mental Health, General Management of Health care Programs, Canary Islands Health Service, Las Palmas de Gran Canaria, Spain; ^9^ Department of Psychiatry, Insular University Hospital of Gran Canaria, Las Palmas de Gran Canaria, Spain

**Keywords:** adherence, antidepressants, depression, depressive disorder, sociodemographic predictors, clinical predictors, systematic review, meta-analysis

## Abstract

**Introduction:** Current evidence reveals concerning rates of non-adherence to antidepressant treatment, possibly influenced by various relevant determinants such as sociodemographic factors or those related to the health system and their professionals. The aim of this paper is to review the scientific evidence on sociodemographic and clinical predictors of adherence to pharmacological treatment in patients diagnosed with a depressive disorder.

**Methods:** a systematic review (SR) was conducted. The search for a previous SR was updated and *de novo* searches were performed in Medline, EMBASE, Web of Science (WoS) and PsycInfo (last 10 years). The risk of bias was assessed using the Cochrane tool for non-randomized studies—of Exposure (ROBINS-E). Meta-analyses were conducted.

**Results:** Thirty-nine studies (*n* = 2,778,313) were included, 24 of them in the meta-analyses. In the initiation phase, no association of adherence was found with any of the predictors studied. In the implementation and discontinuation phases, middle-aged and older patients had better adherence rates and lower discontinuation rates than younger ones. White patients adhered to treatment better than African-American patients.

**Discussion:** Age and ethnicity are presented as the predictive factors of pharmacological adherence. However, more research is needed in this field to obtain more conclusive results on other possible factors.

**Systematic Review Registration:** [https://www.crd.york.ac.uk/prospero/display_record.php?ID=CRD42023414059], identifier [CRD42023414059]

## 1 Introduction

Mood disorders have become a central axis of public health policies due to both their high prevalence and the consequences that this group of disorders have in patients (GBD, 2019 Mental Disorders Collaborator, 2022).

Depressive disorders are a common mental health condition that can have a significant impact on an individual’s overall well-being and daily functioning (World Health Organization, 2017; GBD, 2019 Mental Disorders Collaborator, 2022). This condition results in a reduction in the average life expectancy of 15 years with respect to the population that does not suffer from it (Rivera et al., 2019). In 2019, around 3.9% of the global population suffered from some type of depressive disorder, which translates into a figure of more than 279 million people (Santomauro et al., 2021). On the other hand, persistent depressive disorder, due to the long-lasting manifestation of symptoms, is related to higher rates of comorbidity and a considerable reduction in wellbeing and health-related quality of life (HRQoL) (Nübel et al., 2020).

There is a wide variability of therapeutic options available for the management of depressive disorders. Psychotherapy is indicated for mild to moderate depression, due to its proven effectiveness (NICE, 2022), its long-term superiority, as well as lower dropout rates and lower relapse rates than pharmacological treatments with tricyclic and second generation antidepressants (ADs) (selective serotonin reuptake inhibitors -SSRIs) (Cano-Vindel et al., 2012). However, for the approach and treatment of moderate to severe depressive disorders (Kok and Reynolds, 2017), pharmacological treatment with AD medications, accompanied by a relevant high-intensity psychological intervention is the recommended therapeutic choice (NICE, 2022). Therefore, pharmacological treatment is also among the treatments with proven effectiveness for the management of depression (NICE, 2022). The most recommended current pharmacological regimen, due to its benefit-risk balance, is monotherapy with second-generation ADs, such as SSRIs, among others. Therefore, it should be mentioned that the most recent generations of therapeutic agents have been shown to have higher adherence rates (Sheehan et al., 2008). Nevertheless, most patients do not achieve remission of their symptoms, which is why clinical practice guidelines recommend different second-order options, such as changing monotherapy or combined treatment with two types of ADs (Wolff et al., 2021). However, the effectiveness of a treatment depends on both the efficacy of a medication and patient adherence to the therapeutic regimen (Jimmy and Jose, 2011).

As stated by the World Health Organization (WHO) (World Health Organization, 2004), adherence is defined as the degree to which the person’s behavior-taking medication, following a diet and executing lifestyle changes-corresponds to the agreed recommendations from a healthcare provider. The pharmacological adherence process consists of three phases (Vrijens et al., 2012): initiation, when the patient takes the first dose of a prescribed drug; implementation, defined as the extent to which a patient’s actual dose corresponds to the dose of the prescribed regimen, and discontinuation, when the patient stops the medication on their own initiative, taking no doses thereafter.

Adherence to treatment with ADs significantly impacts the clinical outcomes of the recovery process, with non-adherent patients showing higher rates of relapse, hospitalizations, and visits to the emergency room for events related to depression. This increased need for ongoing medical care imposes a significant burden and economic impact on any healthcare system (Ho et al., 2016), especially considering that, 3 months after starting treatment, the percentage of non-adherent patients ranges from 30% to 70% (Párraga Martínez et al., 2014).

In this context, numerous studies have been carried out to determine the degree of adherence to psychopharmacological treatment with ADs and to analyze its correlates and predictors (Rivero-Santana et al., 2013; Párraga Martínez et al., 2014). The WHO identifies five groups of factors that influence, to a certain extent, the lack of adherence to drug treatment: social and economic factors, therapy-related factors, disease-related factors, patient-related factors, and healthcare system-related factors (Pagès-Puigdemont and Valverde-Merino, 2018). However, current evidence is not consistent regarding the factors relevant to predicting good adherence.

Lack of adherence has serious consequences for patients. Therefore, it is essential to identify the factors that influence the decision-making process regarding the initiation, continuation, or discontinuation of treatment. This information will help enhance current theoretical models and develop more precise and effective interventions tailored to diverse subgroups within the population (Saldaña et al., 2019) at a higher risk of non-adherence (Akincigil et al., 2007). However, the last systematic review (SR) published in this field was conducted 10 years ago (Rivero-Santana et al., 2013), and, thus, updating the available evidence is necessary.

The objective of this systematic review (SR) is to identify, critically evaluate and synthesize the new evidence available in the scientific literature on the sociodemographic and clinical predictive factors influencing adherence to drug treatment in adult patients diagnosed with a depressive disorder.

## 2 Methods

A systematic review (SR) was conducted by updating the search of a previous SR (Rivero-Santana et al., 2013), following the methodology of the Cochrane Collaboration, according to the MECIR (Methodological Expectations of Cochrane Intervention Reviews) standards (Higgins et al., 2016). The information related to this SR is presented following the guidelines of the PRISMA statement (Page et al., 2021). The SR protocol was registered in PROSPERO (registration number: CRD42023414059).

### 2.1 Selection criteria

Studies that evaluated sociodemographic and clinical factors predictive of adherence to AD treatment in patients diagnosed with depressive disorders and which met the selection criteria described below were selected.

Observational studies of prospective and retrospective cohorts were included for the study design. Randomized clinical trials, non-randomized clinical trials, experimental studies with a before-after design, case-control studies, cross-sectional studies, case series and isolated cases, animal studies, and *in vitro* studies were excluded.

The patients included were those over the age of 18 diagnosed with a depressive disorder (ICD-10: F32, depressive episodes; F33, recurrent depressive disorder; F34.1, dysthymia; DSM-V: 296.33, major depressive disorder; 300.4, persistent depressive disorder) by a healthcare provider or by the study investigator. Studies with patients with a manic episode and bipolar affective disorder (ICD-10: F-30-31), schizophrenia, schizotypal, and delusional disorders (ICD-10: F20-29), as well as patients receiving AD treatment without reported diagnosis, were excluded.

The following sociodemographic and clinical variables were considered as predictive factors: age, sex, ethnicity, education, marital status, income, employment status, diagnostic subtype, severity of depression, previous episodes, psychiatric and medical comorbidities, cognitive impairment, and self-perceived health or HRQoL.

Adherence (initiation, implementation and discontinuation) of the pharmacological prescriptions were included as result measures.

Regarding language, only studies published in English and/or Spanish were considered.

As for the type of publication, complete original papers and those published in scientific journals were considered. Conference papers, editorials, conference abstracts, letters to the editor, and opinions were excluded.

### 2.2 Bibliographic search

The search for relevant studies was performed following a search strategy around the terms depressive disorders, antidepressants and adherence in Medline (Ovid platform), EMBASE (Elsevier interface), Web of Science (WoS) (Clarivate Analytics) and PsycInfo (11/09/2022) (see Supplementary Table S1). The search was restricted to studies published in English or Spanish in the last 10 years, the date of the search for the previous SR (Rivero-Santana et al., 2013). The search for published studies was completed with the review of the bibliography lists of the relevant publications retrieved from the electronic databases and with verification in Google Scholar of the studies citing the selected studies.

### 2.3 Study selection processes

The bibliographic references recovered from the different databases were imported into the RAYYAN platform (Ouzzani et al., 2016) where duplicates were eliminated to subsequently select the pertinent studies.

Five reviewers performed the pairwise selection process independently and in parallel. The studies were selected in two phases, a first phase when the studies were selected based on the information provided in the title and abstract; and a second phase when the full texts of the studies selected as relevant in the first phase were analyzed and classified as included or excluded according to the specified selection criteria.

### 2.4 Data extraction processes

Data extraction from the studies was performed using data extraction sheets in Excel format designed *ad hoc*. A pilot test was conducted with two of the studies, independently by the all reviewers, with the aim of unifying extraction criteria. The rest of the extraction from each study was carried out in duplicate.

### 2.5 Data list

Data related to the identification of the article (authors, date of publication, country where the study was conducted, funding, etc.), the design and methodology (objective, design and duration of the study, characteristics and sociodemographic and clinical variables of participants and measure of adherence), as well as predictive factors and adherence, were extracted.

### 2.6 Assessment of risk of bias

The methodological quality of the included studies was assessed independently and in parallel by all reviewers using the Cochrane tool for non-randomized studies - of Exposure, ROBINS-E (ROBINS-E Development Group et al., 2023). Following the guidelines of the ROBINS-E tool, some specific characteristics of the study led directly to the result having a very high risk of bias since the control of confounders did not match the study’s objective. In this SR, this minimal set of confounders include age, sex, and the level of depression.

The graphs for the summary of the risk of bias assessments were drawn with the Rovbis web app (McGuinness and Higgins, 2020).

Disagreements in the selection, extraction and risk of bias assessment phases were resolved after discussion and, if consensus was not reached, a third reviewer was consulted. The discussions and agreements were documented.

### 2.7 Synthesis of the evidence

The information collected was synthesized narratively with tabulation of the results from each included study. A quantitative synthesis using meta-analyses (MA) was performed when the reported data were combinable and the studies were homogeneous in their methodology (population, predictive factors, etc.). To estimate adherence rates (implementation and discontinuation), MA was conducted using the metaprop command (Nyaga et al., 2014) in the STATA software version 17 for Windows (Stata Corp LLC, College Station, TX, United States). To synthesize the predictors of adherence, taking into account the weeks of follow-up, odds ratio (OR) or hazard ratio (HR) and their 95% confidence intervals were synthesized using the generic inverse variance method with the Review Manager software for Windows (RevMan, version 5.4.1., 2020; The Nordic Cochrane Center, The Cochrane Collaboration, Copenhagen, Denmark). MA were performed using univariate estimates only, multivariate estimates only, and preferably univariate or multivariate estimates for each predictor. MA were performed for each predictive factor using both univariate and/or multivariate estimates. An MA was performed exclusively using the respective data type in scenarios where only univariate or multivariate data were available. Conversely, when both univariate and multivariate data were present, preference was given to conducting multivariate estimates. If multivariate data were not available, univariate estimates were preferably used as an alternative. The I^2^ was used to assess statistical heterogeneity. Even so, a random effects model was used to address the inherent variability between studies. In the case of psychiatric comorbidities, the analysis was performed both globally (having a psychiatric comorbidity or not), and separately for different psychiatric comorbidities (sleep disorders vs. alcohol-related disorders vs. substance-related disorders). It was not possible to perform meta-regression or publication bias analyses due to the small number of studies included in each MA.

## 3 Results

The number of references identified during the bibliographic search, once the duplicates were eliminated, came to 1,066. After the title and abstract screening, 58 publications were retrieved for full-text evaluation. After applying the pre-established selection criteria, 45 were excluded. On the other hand, the review of the studies included in the previous SR according to the current selection criteria resulted in 16 additionally included studies. Finally, by hand-examining the bibliography listings of the selected studies, as well as by checking Google Scholar for studies citing the selected studies, an additional 10 studies were located.

**FIGURE 1:**
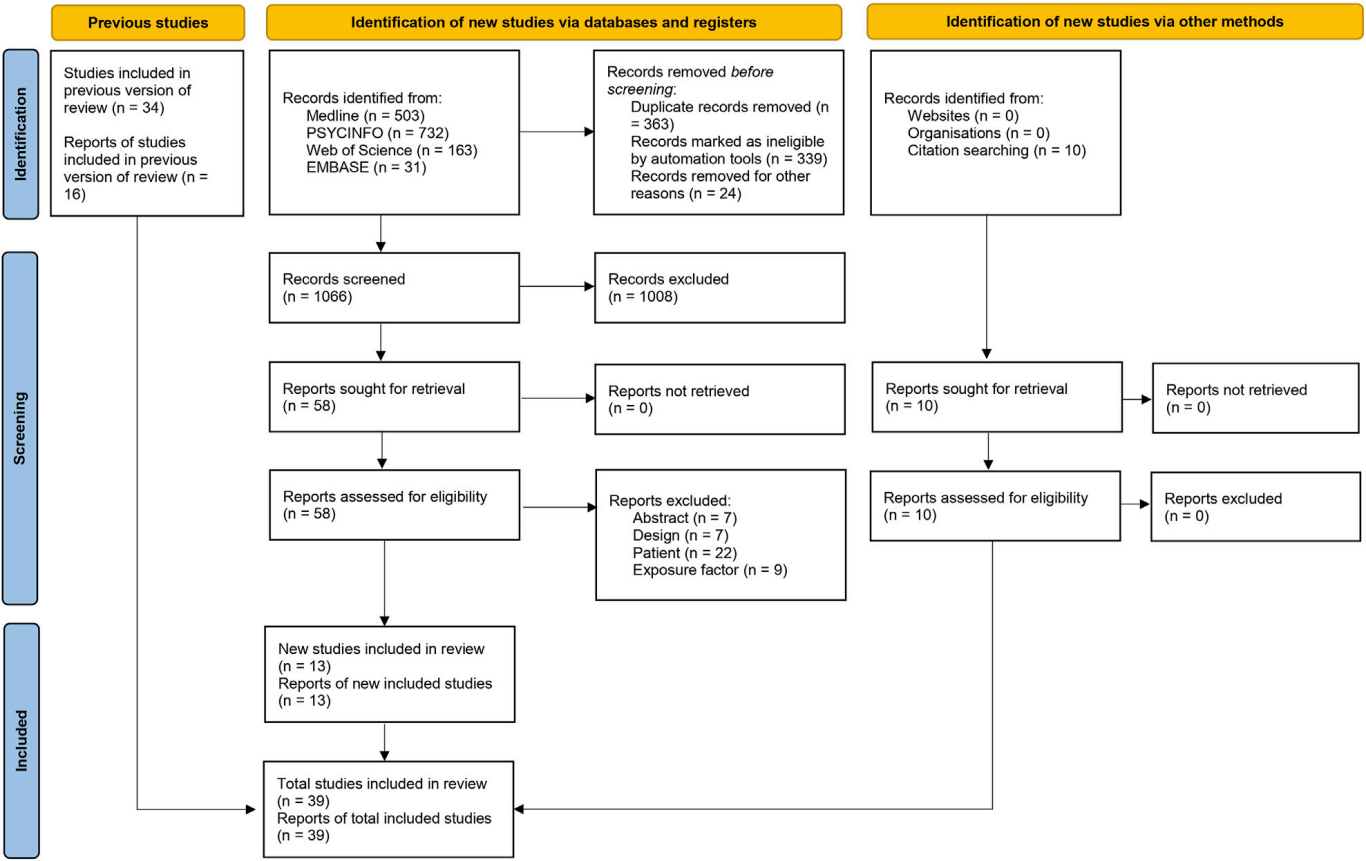
Flow diagram of the selection process of studies.

Thus, 39 studies were included in the final selection (Lin et al., 1995; Lin et al., 2011; Keeley et al., 2000; Keeley et al., 2007; Demyttenaere et al., 2001; Sirey et al., 2001; Cohen et al., 2004; Donohue et al., 2004; Olfson et al., 2006; Akincigil et al., 2007; Goethe et al., 2007; McLaughlin et al., 2007; Stang et al., 2007; ten Doesschate et al., 2009; Yen et al., 2009; Chen et al., 2010; Ereshefsky et al., 2010; Holma et al., 2010; Liu et al., 2010; Liu et al., 2011; Milea et al., 2010; Woolley et al., 2010; Hung et al., 2011; Vlahiotis et al., 2011; Merrick et al., 2012; Wu et al., 2012; Wu et al., 2013; Kales et al., 2013; Kales et al., 2016; Wu and Davis-Ajami (2014); Yau et al., 2014; Kogut et al., 2016; Gerlach et al., 2017; Gerlach et al., 2019; Holvast et al., 2019; Bhattacharjee et al., 2020; Nam-Ju and Yeon-Pyo, 2020; Noh et al., 2020; Noh et al., 2022; Shin et al., 2022) (See Figure 1).

Although many of these articles were excluded because they did not meet more than one selection criteria, Supplementary Table S2 shows the main reason for their exclusion.

### 3.1 Characteristics of included studies

The characteristics of the studies, participants, predictive factors and adherence can be seen in more detail in Tables 1, 2, 3; however, a description of the main characteristics is provided below.

**TABLE 1:** General characteristics of the included studies.

Study	Country	Setting	Funding	Design	No of cohorts	Follow-up (weeks)
Akincigil et al. (2007)	United States	Database	No	RCS	1	33
Bhattacharjee et al. (2020)	United States	Database	No	RCS	1	52
Chen et al. (2010)	United States	Database	NR	RCS	1	39
Cohen et al. (2004)	Canada	Psychiatric center	Yes	RCS	1	14
Demyttenaere et al. (2001)	Belgium	Primary care	Yes	RCS	1	24
Donohue et al. (2004)	United States	Database	No	RCS	1	26
Ereshefsky et al. (2010)	United States	Database	Yes	RCS	1	26
Gerlach et al. (2017)	United States	Primary care and veteran center	No	PCS	1	16
Gerlach et al. (2019)	United States	Veterans center	No	RCS	1	52
Goethe et al. (2007)	United States	Database	Yes	PCS	1	12
Holma et al. (2010)	Finland	Psychiatric center	No	PCS	1	260
Holvast et al. (2019)	Netherlands	Database	No	RCS	1	42
Hung et al. (2011)	Taiwan	Hospital	No	PCS	1	24
Kales et al. (2013)	United States	Primary care	No	PCS	2	16
Kales et al. (2016)	United States	Veterans center	No	PCS	1	16
Keeley et al. (2000)	United States	Primary care	NI	RCS	1	14
Keeley et al. (2007)	United States	Primary care	No	PCS	1	12
Kogut et al. (2016)	United States	Database	No	RCS	1	12
Lin et al. (1995)	United States	Primary care	No	RCS	1	16
Lin et al., 2011	United States	Database	NI	RCS	1	52
Liu et al. (2010)	United States	Database	Yes	RCS	3	52
Liu et al. (2011)	United States	Database	Yes	RCS	1	52
McLaughlin et al. (2007)	United States	Database	Yes	RCS	1	39
Merrick et al. (2012)	United States	Database	No	RCS	1	16
Milea et al. (2010)	France	Database	NR	RCS	1	52
Nam-Ju and yeon-pyo (2020)	South Korea	Database	No	RCS	1	26
Noh et al. (2022)	South Korea	Database	No	RCS	1	26
Olfson et al. (2006)	United States	Database	No	RCS	1	12
Shin et al. (2022)	South Korea	Database	No	RCS	2	26
Sirey et al. (2001)	United States	Outpatient clinic	No	RCS	1	12
Stang et al. (2007)	United Kingdom	Database	Yes	RCS	2	39
ten Doesschate et al. (2009)	Netherlands	Psychiatric center	No	PCS	1	104
Vlahiotis et al. (2011)	United States	Database	No	RCS	1	26
Woolley et al. (2010)	United States	Hospital	Yes	PCS	1	12
Wu et al. (2012)	United States	Database	No	RCS	1	52
Wu et al. (2013)	Taiwan	Database	No	RCS	1	26
Wu and Davis-Ajami, 2014	United States	Database	No	RCS	1	26
Yau et al. (2014)	China	Hospital	No	RCS	1	52
Yen et al. (2009)	Taiwan	Database	NR	PCS	1	52

Note: Funding: industry funding; N: number; NR: NR: not reported; PCS: prospective cohort study; RCS: retrospective cohort study.

**TABLE 2:** Main demographic and baseline clinical characteristics of the participants in the included studies.

Study	Diagnosis of depression	Population subgroup	Inclusion criteria	Exclusion criteria	N	N loss	N women (%)	Mean (SD) range
Akincigil et al. (2007)	MDE	Adults	1. ≥ 18 years	NR	4312	0	2907 (67.42)	NR
			2. New MDE					
			3. New indication AD.					
Bhattacharjee et al. (2020)	CS depression with dementia	Older adults	1. ≥ 65 years	1. End-stage renal disease	6239	0	4666 (74.79)	NR
			2. Dementia	2. Liver disease				
			3. Depression CS or greater (ICD-9-CM: 296.2, 296.3, 309.1, 300.4 or 311)					
Chen et al. (2010)	MDD	Adults	1. MDD (ICD-9-CM: 296.20-296.24)	1. Age < 18	4102	0	2679 (65.31)	40 (12)
				2. Bipolar disorder or schizophrenia				
			2. AD second generation	3. AD (previous 6 months)				NR
				4. AD empowerment				
Cohen et al. (2004)	MDE	Adults	1. MDE	1. Substance abuse or dependence (previous 6 months)	65	57	34 (52.31)	41.4 (11.4)
			2. AD.	2. Bipolar disorders or schizophrenia (previous 12 months)				NR
				3. CS unstable medical condition				
Demyttenaere et al. (2001)	MDD	Adults	1. 18–65 years	NR	272	0	196 (72.06)	43 (13)
			2. MDD (DSM-IV-TR)					NR
			3. AD.					
Donohue et al. (2004)	MDD	Adults	1. 18–64 years	1. Bipolar disorder or schizophrenia	36062	0	24342 (67.5)	44 (NR)
			2. AD	2. No AD medical coverage				NR
			3. MDD (DSM-IV)					
Ereshefsky et al. (2010)	Depression	Adults	1. ≥ 18 years	NR	45481	0	NR	NR
			2. SSRIs					
			3. Depression (ICD-9: 296.2, 296.3, 300.4 or 311.x9)					
			4. Without AD (6 months before)					
Gerlach et al. (2017)	CS depression	Older adults	1. ≥ 60 years	1. Bipolar disorder or schizophrenia	452	0	108 (23.98)	NR
			2. CS depression (PHQ-9 > 5)	2. Cognitive impairment				
			3. Start of AD.	3. Suicidal risk				
Gerlach et al. (2019)	CS depression	Older adults	1. ≥ 60 years	1. Bipolar disorder or schizophrenia	278	12	8 (2.88)	65 (6.3)
			2. CS depression (PHQ-9 > 5)	2. Cognitive impairment				NR
			3. New AD prescription	3. Suicidal risk				
Goethe et al. (2007)	MDD	Mixed population	1. MDD (DSM-IV: 296.2x or 296.3x)	1. Bipolar disorder or schizophrenia or dementia	445	39	291 (65.39)	41.0 (12.7)
			2. 18–75 years	2. Electroconvulsive therapy				18–75
			3. SSRI.	3. ≥ 1 AD.				
Holma et al. (2010)	MDD	Adults	1. Depressive symptoms (previous 18 months)	NR	542	360	128 (23.62)	41.5 (11.1)
			2. MDD (DSM-IV)					NI
Holvast et al. (2019)	Depression	Older adults	1. ≥ 60 years	NR	1512	0	1052 (69.58)	68* (NR)
			2. Depression (ICPC P03 or P76)					63–75*
Hung et al. (2011)	MDD	Mixed population	1. 18–65 years old	1. Substance dependence or abuse (prior 1 month)	135	0	101 (74.81)	30.2 (NR)
			2. MDD (DSM-IV-TR)	2. Psychotic, catatonic symptoms or psychomotor retardation				18–65
				3. Chronic medical conditions				
Kales et al. (2013)	CS depression	Older adults	1. ≥ 60 years	1. Suicidal ideation, bipolar disorder or schizophrenia or impairment	198	10	102 (51,52)	67.3 (NR)
			2. CS depression (GDS ≥ 5)	2. Not Caucasian or African-American				NR
			3. New AD.	3. Not English				
Kales et al. (2016)	CS depression	Older adults	1. ≥ 60 years	1. Cognitive decline	311	0	8 (2.57)	64.9 (6.3)
			2. Depression (PHQ-9 > 5)					60–86
			3. AD (1 week)					
Keeley et al. (2000)	Depression	Adults	1. Start of AD	1.Organic mental disorders	30	0	23 (76.67)	41.2 (12.9)
				3. Not English				
			2. Depression	3. Suicidal risk				NR
				4. Bipolar disorder				
Keeley et al. (2007)	MDD	Adults	1. TDM (DSM-IV, PHQ-9)	1. Pregnant or nursing	20	0	14 (70)	48.3 (8.6)
			2.AD	2. Bipolar disorder				NR
			3. English	3. Cognitive impairment				
			4. ≥ 18 years					
Kogut et al. (2016)	Depression	Adults	1. New AD	NR	1983	0	1502 (75.74)	NR
			2. Depression					
Lin et al. (1995)	Episode of depression	Mixed population	1. 18–65 years old	NR	164	NR	118 (71.95)	47 (NR)
			2. A new AD prescription					18–75
			3. Depression					
Lin et al. (2011)	MDD	Adults	1. MDD (ICD-9 codes: 296.2x or 296.3x)	1. Bipolar disorder	2111615	0	64678 (30,63)	NR
			2. AD.	2. Mood stabilizers and antipsychotics				
				3. Childish				
Liu et al. (2010)	MDD	Adults	1. Start of duloxetine	NR	6132	0	4539 (74.02)	45.6–47.2 (NR)
			2. MDD (ICD-9-CM: 296.2 or 296.3) 1 year before duloxetine					18–64
			3. 18–64 years old					
			4. Insured (≥12 months)					
Liu et al. (2011)	MDD	Adults	1. Start of SNRI or SSRI	1. >1 SNRI or SSRI.	44026	0	31366 (71.24)	NR
			2. MDD (ICD-9-CM: 296.2 or 296.3)					
			3. 18 to 64 years					
McLaughlin et al. (2007)	Depression	Adults	1. ≥ 18 years	1. Prior use of AD (previous 9 months)	3138	0	2219 (70.71)	46.18 (13.94)
			2. Depression (ICD-9-CM: 296.2, 296.3, 300.4 or 311)					NR
Merrick et al. (2012)	Depression	Adults	1. ≥ 18 years	1. Bipolar disorder	383	0	276 (72.06)	NR
			2. Depressive disorders (ICD-9-CM: 296.20-296-.25, 296.30-296.35, 298.0, 300.4 or 309.1, 311)					
			3. New AD prescription					
Milea et al. (2010)	DD	Adults	1. New episode	1. Combined treatment	134287	0	91485 (68.13)	NR
			2. New DD (ICD-9-CM: 296.2, 296.3, 300.4 or 311)					
			3. Monotherapy					
Nam-Ju and yeon-pyo (2020)	Depression	Adults	1. Depression (ICD-10: F32.x, F33.x or F34.1)	1. Bipolar disorder or schizophrenia	142336	NR	91800 (64.50)	NR
			2. ≥ 1 AD.					
Noh et al. (2022)	Depression	Pregnant	1. Women	1. AD not prescribed (30 days prior)	5207	0	5207 (100)	32.3 (4.8)
			2. 15–50 years					
			3. One or more live births					NR
			4. Depression (ICD-10: F32.x, F33.x, F34.1x or F41.2x)					
Olfson et al. (2006)	Depression	Adults	1. ≥ 18 years	NR	390	0	258 (66.15)	NR
			2. Depression (ICD-9-CM: 296.2, 296.3, 300.4 or 311)					
Shin et al. (2022)	Depression	Adults	1. ≥ 19 years	1. Previous depression	176745	0	115458 (65.32)	NR
			2. Depression (ICD-10: F32–34 or F43)					
Sirey et al. (2001)	MDD	Adults	1) MDD	1. Cognitive impairment	1242	NR	82 (6.6)	NR
			2) Seeking treatment	2. Alcohol or substance abuse (prior 1 month)				
				3. Another axis I disorder				
Stang et al. (2007)	Depression	Adults	1) 18–64 years	1. Benzodiazepines or AD (previous 6 months)	2991	NR	1898 (63.46)	40.84 (NR)
			2) Depression (ICD-9-CM: 296.2, 296.3, 300.4 or 311)					NR
			3) Bupropion					
ten Doesschate et al. (2009)	MDE	Adults	1. ≥ 2 MDE (last 5 years - DSM-IV)	1. Bipolar disorder or schizophrenia	172	81	NR	NR
			2. Current referral status	2. Organic brain damage, alcohol or substance abuse				
			3. HAM-D < 10	3. Anxiety disorder				
				4. Cognitive electroconvulsive therapy or psychotherapy				
Vlahiotis et al. (2011)	An episode or rMDD	Adults	1. New SSRI or SNRI	NR	16659	0	10885 (65.34)	NR
			2. ≥ 18 years					
			3. Single episode or rMDD.					
Woolley et al. (2010)	MDD	Mixed population	1. 18–75 years	1. Bipolar disorder, schizophrenia or dementia	403	NR	290 (71.96)	41 (NR)
			2. SSRIs	2. Electroconvulsive therapy				NR
			3. MDD (DSM-IV: 296.2x or 296.3x)	3. ≥ 1 AD.				
Wu et al. (2012)	MDD	Adults	1. 18 and 64 years old	1. Bipolar disorder	3083	0	2384 (77.33)	18–64
			2. MDD (ICD-9-CM: 296.2 or 296.3)					
Wu et al. (2013)	DD	Adults	1. DD (ICD-9-CM: 296.2, 296.3 or 300.4)	1. Bipolar disorder, schizophrenia or dementia	25744	NR	16244 (63.1)	43.6 (16.4)
				2. Antipsychotics or mood stabilizers				NR
				3. ≥ 1 types of antidepressants on the index date				
Wu and Davis-Ajami (2014)	Depression	Pregnant	1. Pregnant	1. AD (previous 6 months)	804	0	804 (100)	25.8 (6.2)
			2. ≥ 18 years					
			3. Single or multiparous live births					
			4. Depression (ICD-9-CM: 296.2, 296.3, 300.4 or 311)	2. Bipolar disorder or schizophrenia				NR
			5. Use of AD 280 days before calving					
			6. ≥ 2 AD prescriptions during pregnancy					
Yau et al. (2014)	MDD	Adults	1. ≥ 18 years	1. Another axis I disorder	189	0	71 (37.57)	46.1 (14.8)
			2. AD	2. Dementia or mental retardation				20–88
			3. MDD (ICD-10)	3. AD (previous 6 months)				
				4. Follow-up by psychiatry				
				5. History of overdose or suicide				
Yen et al. (2009)	DD	Adults	1. DD (DSM-IV)	1. Mental retardation	164	43	81 (49.39)	42.7 (12.9)
			2. CES-D ≥17	2. Substance use				17–75
				3. Psychotic disorders				

AD: antidepressant; Older adults: adults >60 years; CES-D; depression scale of the center for epidemiological studies; CS: clinically significant; DD: depressive disorder; ICD: international classification of diseases; MDE: major depressive episode; GDS: geriatric depression scale; HAM-D: hamilton scale for depression; SSRIs: Selective Serotonin Reuptake Inhibitors; SNRI: serotonin and norepinephrine reuptake inhibitors; NR: not report; PHQ-9: patient health questionnaire; MDD: major depressive disorder; rMDD: recurrent major depressive disorder.

**TABLE 3:** Main characteristics of the predictive factors and the measure of adherence.

	Predictive factors					Adherence				
Study	Measure moment (week)	Sociodemographic	Definition	Clinics	Definition	Initiation	Implementation	Discontinuation	Adherence measure	Adherence criteria
Akincigil et al. (2007)	16, 33	Sex		Medical comorbidity	Alcohol/substances; cancer; migraine; CVD/diabetes	No	Yes	No	MPR	≤ 75%
		Age	18–25; 25–39; 40–49; 50–66; ≥ 65	Psychiatric comorbidity	Anxiety					
Bhattacharjee et al. (2020)	16, 50	Age	65–74; ≥ 75	NA	NA	No	Yes	No	MPR	≤ 80%
		Sex								
		Ethnicity	White vs. others							
Chen et al. (2010)	39	Age	18–34; 35–49; 50–64; ≥ 65	Psychiatric comorbidity	Anxiety	No	Yes	No	MPR	≤ 80%
		Sex		Medical comorbidity	Alcohol/substances					
Cohen et al. (2004)	14	Age	Years	Severity of depression	MDE status	No	Yes	No	*Medication Event Monitoring System*	Continuous (% days of container opening/prescription days)
		Sex		Previous episodes	Previous MDE					
Demyttenaere et al. (2001)	24	Age	Years	NA	NA	No	No	Yes	Self-report	Continue with the medication
		Sex								
Donohue et al. (2004)	26	Age	NA	Diagnostic subtype	NI	No	Yes	No	Prescription record	≥ 60 days
Ereshefsky et al. (2010)	26	Age	18–34; 35–49; 50–64	Psychiatric comorbidity	Alcohol/substances	No	No	Yes	Prescription record	≥ 30 days
Gerlach et al. (2019)	52	Age	60–64; 65–74; 75–90	Psychiatric comorbidity	PTSD; Anxiety; Substances	No	Yes	No	BMQ	≤ 80%
		Sex	Male/female	Medical comorbidity	CCI					
		Ethnicity	White vs. African-American							
		Education	Some higher education							
		Civil status	Spouse/partner vs. Single/no partner							
Gerlach et al. (2017)	16	Sex		NA	NA	No	Yes	No	MPR	≤ 80%
		Ethnicity	White vs. African-American	NA	NA					
Goethe et al. (2007)	12	Sex		Psychiatric comorbidity	Anxiety	No	No	Yes	Self-report	Yes/no
		Age	18–75							
		Ethnicity	White/No white							
		Employment situation	Presence (yes/no)	NA	NA					
Holma et al. (2010)	26, 78, 260	Civil status	Living alone (yes/no)	NA	NA	No	Yes	No	Self-report	1. Regularly; 2. Something irregular, no; 3. Very irregularly; 4. Not at all
Holvast et al. (2019)	2, 42, 52	Age	Years	Psychiatric comorbidity	Presence (yes/no)	Yes	Yes	Yes	MPR	≤ 80%
		Sex	NA	Medical comorbidity	Number					
		Income	Socioeconomic level							
Hung et al. (2011)	16	Age	Continuous	Severity of depression	Chronic (yes/no)	No	No	Yes	Self-report	Continue with medication
		Sex		Medical comorbidity	Migraine					
		Education	Years	Psychiatric comorbidity	Anxiety					
		Employment situation	Unemployed or employed							
Kales et al. (2013)	16	Ethnicity	White; African-American	NA	NA	No	Yes	No	BMQ	Skip ≥ 2 daily doses
Kales et al. (2016)	16	Ethnicity	White; African-American	Medical comorbidity	CCI	No	Yes	No	MPR + BMQ	≤ 80%
		Civil status	Partner (yes/no)							
Keeley et al. (2000)	14	Age	Years	Medical comorbidity	Number	No	No	Yes	Self-report	Continue with medication
		Ethnicity	Hispanic (yes/no)							
Keeley et al. (2007)	12	Age	NA	Psychiatric comorbidity	Somatoform disorder	No	Yes	Yes	Self-report + Prescription record	Continuous (% days supplied/total days) × 100
		Sex		Medical comorbidity	NA					
		Ethnicity	NI							
		Employment situation	Presence (yes/no)							
		Education								
Kogut et al. (2016)	12	Age	18–34; ≥ 35	NA	NA	No	Yes	No	MPR	≤70%
		Sex		NA	NA					
Lin et al. (1995)	4, 16	Sex		Severity of depression	Dysthymia	No	Yes	No	Self-report	NI
					Number of episodes					
		Age	Years							
		Education	Years							
Lin et al. (2011)	52	Sex		Psychiatric comorbidity	Psychotic disorders	No	Yes	No	Prescription record	Continuous (% days supplied/365)
					Anxiety					
		Age	28–25; 26–49; 50–64; ≥ 65	NA	NA					
		Ethnicity	Non-Hispanic White; Non-Hispanic Black; Hispanic; Other							
		Income	< $20000; $20000-$40000; $40000-$60000; > $60000							
Liu et al. (2010)	52	Age	18–25; 26–35; 36–45; 46–55; 56–64	Perceived health perceived health	NA	No	Yes	Yes	MPR	≤ 80%
Liu et al. (2011)	52	Age	18–25; 26–35; 36–45; 46–55; 56–64	Medical comorbidity	Headaches and lower back	No	Yes	Yes	MPR	≤ 80%
		Sex		Psychiatric comorbidity	Fibromyalgia, hypersomnia, Alcohol/Substances					
McLaughlin et al. (2007)	39	Age	Years	NA	NA	No	Yes	No	Prescription record	≤ 70%
		Sex								
Merrick et al. (2012)	16	Age	49–59; 60–74; ≥ 75	Diagnostic subtype	Major depression (yes/no)	No	Yes	No	Prescription record	≤ 70%
		Sex		Medical comorbidity	CCI					
		Raza	White; not white							
Milea et al. (2010)	4, 42	Age	< 18; 18–39; 40–64; ≥ 65	NA	NA	No	No	Yes	Prescription record	≠ days dispensing and prescription
		Sex								
Nam-Ju and yeon-pyo (2020)	12; 26	Income	Class 1–5	NA	NA	No	Yes	No	MPR	≤ 80% (non-adherent)
Noh et al. (2022)	26	Age	Years	Psychiatric comorbidity	Psychotic, anxiety, stress, substance, eating, personality and sleep disorder	No	No	Yes	Prescription record	≥ 45 days
				Medical comorbidity	CVD/diabetes/epilepsy					
Olfson et al. (2006)	12	Age	18–44; 45–64; ≥ 65	NA	NA	No	No	Yes	Self-report	≥ 30 days
		Sex	NA							
		Ethnicity	White; black; Hispanic; other							
		Civil status	Married; not married; divorced or separated; widower							
		Employment situation	Unemployed (yes/no)							
Shin et al. (2022)	26	Age	19–34; 35–49; 50–64; ≥65	NA	NA	No	Yes	No	1. MPR	1. ≤ 80% (non-adherent)
		Sex							2. Duration	2. ≥ 39 days
Sirey et al. (2001)	12	Age	< 60, ≥ 60	Severity of depression	NI	No	Yes	No	Self-report + Prescription record	Likert scale 6 + concordance with pill count
Stang et al. (2007)	39	Sex		NA	NA	No	Yes	No	Prescription record	≤ 70%
		Age	Years							
ten Doesschate et al. (2009)	104	Sex		Medical comorbidity	Presence (yes/no)	No	Yes	No	MAQ	Score
		Age	Years	Previous episodes	Number					
		Civil status	Lives alone (yes/no)	Severity of depression	HAM-D					
		Employment situation	Presence (yes/no)							
		Education	Superior/other							
Vlahiotis et al. (2011)	26	Sex	NA	Medical comorbidity	CCI	No	No	Yes	Prescription record	Days supplied/days dispensed
		Age	18–25; 26–40; 41–55; 56–64	Psychiatric comorbidity	Anxiety, bipolar disorder and OCD					
Woolley et al. (2010)	NI	Sex		NA	NA	No	No	Yes	Self-report	Continue with medication
		Age	Years							
Wu et al. (2012)	52	Age	18–30; 31–40; 41–50; 51–60; 61–64	Psychiatric comorbidity	Anxiety	No	Yes	No	1. MPR	≤ 80%
		Ethnicity	Caucasian; Afro-American	Medical comorbidity	0, 1, 2, o ≥ 3				2. Duration	≤ 15 days
		Sex								
Wu et al. (2013)	4, 12, 26	Age	18–44, 45–64, ≥ 65	Psychiatric comorbidity	Anxiety, sleep disorder, alcohol/substances	No	No	Yes	Prescription record	≥ 30 days
		Sex		Medical comorbidity	CCI					
Wu and Davis-Ajami (2014)	26	Age	Years	Medical comorbidity	CCI	No	Yes	No	Prescription record	≤ 80%
		Ethnicity	White; not white							
Yau et al. (2014)	26	Age	Years	NA	NA	No	Yes	No	Prescription record	≤ 80%
		Sex								
Yen et al. (2009)	52	Sex		Diagnostic subtype	Major depression	No	Yes	No	Medication Adherence Behavior Scale	Score
		Age	Years							
		Education	Years							

BMQ: brief medication questionnaire; CCI: charlson comorbidity index; CVD: cardiovascular disease; MAQ: medication adherence questionnaire; MPR: medication possession ratio; NA: not applicable; NR: not report; OCD: obsessive-compulsive disorder; PTSD: post-traumatic stress disorder.

All included studies were published in English between the years 1995 and 2022. The countries where such studies were published were: United States (25 studies), South Korea (3 studies); Taiwan (3 studies), the Netherlands (2 studies), Belgium (1 study), Canada (1 study), China (1 study), Finland (1 study), France (1 study) and the United Kingdom (1 study).

In terms of design, 12 were prospective observational cohort studies (Demyttenaere et al., 2001; Cohen et al., 2004; Goethe et al., 2007; Keeley et al., 2007; ten Doesschate et al., 2009; Yen et al., 2009; Holma et al., 2010; Woolley et al., 2010; Hung et al., 2011; Kales et al., 2013; Kales et al., 2016; Gerlach et al., 2017) and the remaining 27 were retrospective observational cohort studies.

Of all the selected studies, the majority, 35 (89.7%), included one cohort, three studies (7.69%) included two cohorts (Stang et al., 2007; Kales et al., 2013; Shin et al., 2022) and one study included three cohorts (Liu et al., 2010). Multiple cohort studies were compared based on characteristics such as the dose or type of medication, ethnicity, and the healthcare insurance coverage (e.g., uninsured, partially or fully insured).

The studies were carried out in psychiatric settings (Cohen et al., 2004; ten Doesschate et al., 2009; Holma et al., 2010), primary care centers (Lin et al., 1995; Keeley et al., 2000; Keeley et al., 2007; Demyttenaere et al., 2001; Kales et al., 2013; Gerlach et al., 2017), hospitals (Woolley et al., 2010; Hung et al., 2011; Yau et al., 2014), veterans clinics (Kales et al., 2016; Gerlach et al., 2017; Gerlach et al., 2019), outpatient clinics (Sirey et al., 2001), while the rest were conducted with database records.

Of the studies selected for this review, 23.08% received industry funding (Demyttenaere et al., 2001; Cohen et al., 2004; Goethe et al., 2007; McLaughlin et al., 2007; Stang et al., 2007; Ereshefsky et al., 2010; Liu et al., 2010; Liu et al., 2011; Woolley et al., 2010), 64.10% did not receive funding from industry and the source of their funding is unknown in 12.82% of the studies.

Follow-up periods were variable, with the closest follow-up being 12 weeks after starting treatment (Sirey et al., 2001; Olfson et al., 2006; Goethe et al., 2007; Keeley et al., 2007; Woolley et al., 2010; Kogut et al., 2016) and the longest period was 260 weeks (Holma et al., 2010). The information regarding the characteristics of the studies can be seen below in Table 1.

Regarding the predictive factors, more specifically the sociodemographic ones, of the 39 studies selected for this SR, 34 analyzed the effect of age on adherence to treatment, 28 the effect of sex, 13 studies analyzed ethnicity, six studies explored the influence of educational level, five of marital status, and five studies of employment status.

Regarding clinical factors, of the 39 studies, 13 analyzed the relationship of psychiatric comorbidities on adherence to treatment, 16 medical comorbidities, five the severity of depression, two the relationship of previous episodes, three the subtype of diagnosis and one perceived health on adherence.

In relation to the phases of adherence, of all the selected studies, only one studied the adherence initiation phase (Holvast et al., 2019), 29 studied the implementation phase and 16 the discontinuation phase.

The selected studies used different tools to measure adherence in the implementation phase. By using the Medication Possession Ratio (MPR), nine studies established a threshold of 80%, one study a threshold of 75% and another one a threshold of 70%; two studies used the Brief Medication Questionnaire; one study used the Medication Adherence Behavior Scale; one the Medication Event Monitoring System - Pill Count; one the Medication Adherence Questionnaire (MAQ); three used self-reports developed *ad hoc* and eight used prescription records.

The information described above is shown in more detail in Table 3 below.

### 3.2 Risk of bias assessment

In general, the risk of bias was considered very high in 20 of the studies in this SR due to the lack of control over significant confounding variables such as age, sex, and the severity of depression. In the rest of the fully evaluated articles, the risk of bias was high in four studies, low in 13 studies, while only one study presented unclear risk of bias.

**FIGURE 2:**
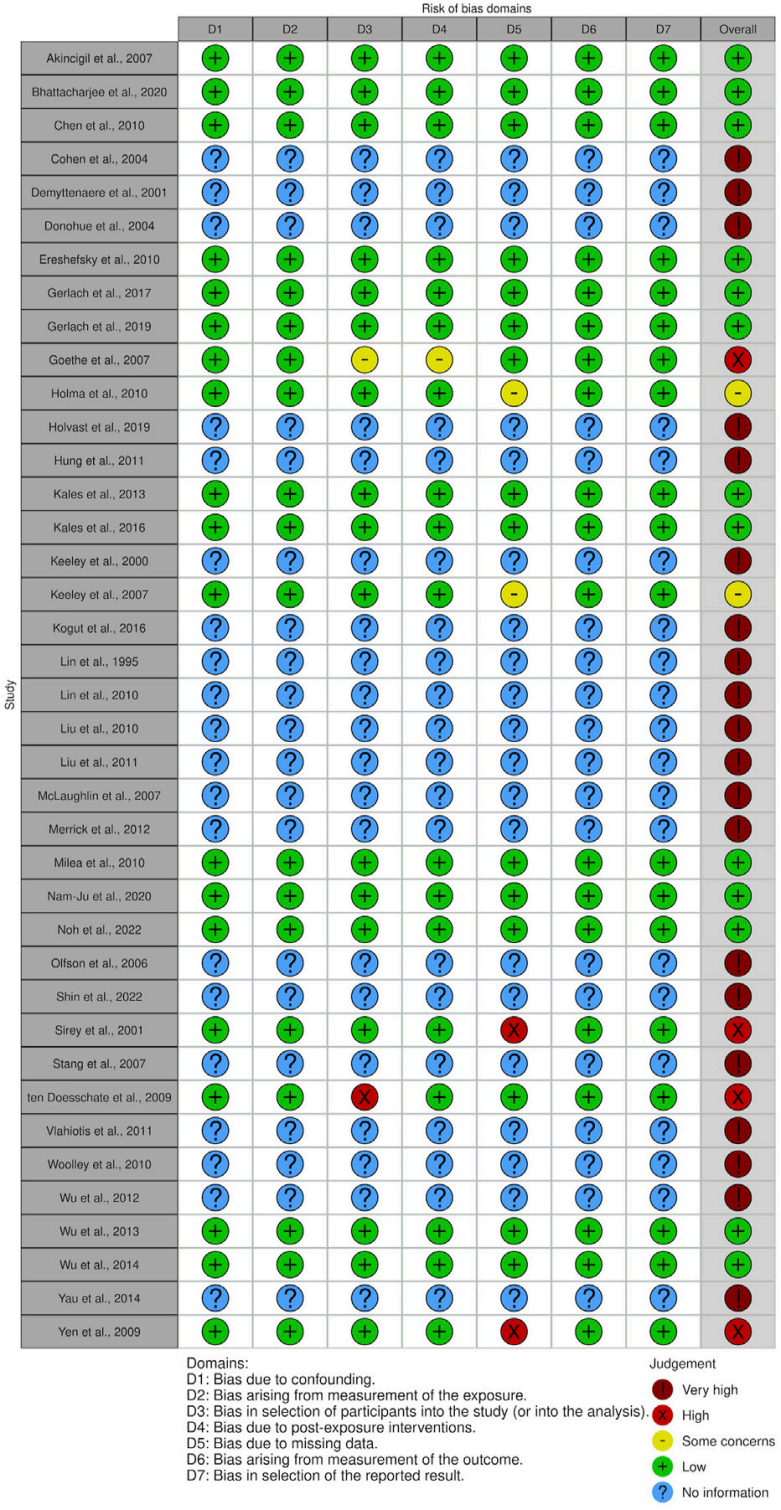
Risk of bias assessment of included studies.

**FIGURE 3:**
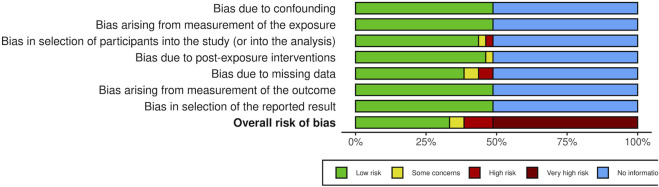
Risk of bias assessment across included studies.

Detailed judgments for each of the risk of bias domain criteria are shown in Figures 2, 3.

### 3.3 Evidence synthesis

The evidence tables included in Supplementary Tables S3–S6 show the main findings obtained in the included studies.

Of the total number of included studies, 24 could be included in the MA (Donohue et al., 2004; Olfson et al., 2006; Akincigil et al., 2007; Goethe et al., 2007; Stang et al., 2007; ten Doesschate et al., 2009; Yen et al., 2009; Chen et al., 2010; Ereshefsky et al., 2010; Liu et al., 2010; Liu et al., 2011; Milea et al., 2010; Woolley et al., 2010; Vlahiotis et al., 2011; Merrick et al., 2012; Wu et al., 2012; Wu et al., 2013; Kales et al., 2013; Kales et al., 2016; Yau et al., 2014; Kogut et al., 2016; Gerlach et al., 2019; Holvast et al., 2019; Noh et al., 2022). Tables 4, 5 show the results of the estimation of the global effect size for the outcome measures that could be meta-analyzed (see forest plots in Supplementary Figures S1–S13).

**TABLE 4:** Results of the meta-analyses. Implementation.

EXPOSURE FACTOR/*Variable*	Model	K	OR/HR*	95% CI	I^2^ (%)	Test for subgroup differences (%) (*p*-value)
AGE						
Subgroup: 25–50 years by follow-up (*ref.: 18–24* *years*)						
Total	Random	2	1.02	(0.69, 1.52)	67	NA
*39–52* *weeks, Univariate*	Random	2	1.02	(0.69, 1.52)	67	NA
Subgroup: 35–49 years by follow-up (*ref.: 18–34* *years*)						
Total (*ref.: 18–34* *years*)	Random	7	1.47	(1.40, 1.55)	1	0 (0.61)
12–16 weeks, *Univariate*	Random	2	1.51	(1.23, 1.85)	58	NA
33–39 weeks, *Univariate*	Random	2	1.35	(1.11, 1.63)	0	NA
39–52 weeks, *Preferably multivariate*	Random	3	1.49	(1.38, 1.62)	15	NA
Subgroup: 50–65 years by follow-up (*ref.: 18–34* *years*)						
Total (*ref.: 18–34* *years*)	Random	4	1.73	(1.29, 2.32)	79	0 (0.72)
12–16 weeks, *Univariate*	Random	2	1.85	(1.05, 3.26)	93	NA
33–39 weeks, *Univariate*	Random	2	1.65	(1.31, 2.09)	4	NA
39–52 weeks, *Univariate*	Random	6	2.03	(1.91, 2.15)	0	NA
Subgroup: >65 years by follow-up (*ref.: 18–34* *years*)						
Total (*ref.: 18–34* *years*)	Random	4	1.80	(1.29, 2.53)	46	75 (0.05)
12–16 weeks, *Univariate*	Random	2	2.23	(1.61, 3.10)	13	NA
33–39 weeks, *Univariate*	Random	2	1.32	(0.88, 1.97)	0	NA
SEX						
Subgroup: sex by follow-up (*ref.: male*)						
Total (*ref.: male*)	Random	13	1.07	(1.03, 1.11)	0	0 (0.52)
12–16 weeks, *Preferably multivariate*	Random	4	1.10	(1.01, 1.20)	0	NA
26–39 weeks, *Univariate*	Random	5	1.10	(0.98, 1.24)	47	NA
52 weeks, *Preferably multivariate*	Random	4	1.05	(1.00, 1.10)	0	NA
ETHNICITY						
Subgroup: ethnicity (African American) by follow-up (*ref.: white*)						
Total	Random	5	2.19	(1.63, 2.94)	42	44.8 (0.18)
16 weeks, *Multivariate*	Random	3	2.67	(1.86, 3.83)	0	NA
52 weeks, *Multivariate*	Random	2	1.85	(1.25, 2.74)	37	NA
PSYCHIATRIC COMORBIDITY						
Subgroup: anxiety by follow-up (*ref.: no*)						
Total	Random	6	1.14	(0.97, 1.34)	64	84 (0.002)
12–16 weeks, *Univariate*	Random	2	1.02	(0.90, 1.15)	0	NA
33–39 weeks, *Univariate*	Random	2	1.04	(0.87, 1.24)	0	NA
52 weeks, *Preferably multivariate*	Random	2	1.50	(1.25, 1.81)	0	NA

Note: HR: hazard ratio; NA: not applicable OR: odds ratio; Random: random effect; ref.: reference.

**TABLE 5:** Results of the meta-analyses. Discontinuation.

EXPOSURE FACTOR/*Variable*	Model	K	OR/HR*	95% CI	I^2^ (%)	Test for subgroup differences (%) (*p*-value)
AGE						
*Continuous*						
Total	Random	2	0.98	(0.97, 0.99)	0	NA
12 weeks, *Multivariate*	Random	2	0.98	(0.97, 0.99)	0	NA
Subgroup: 25–40 years by follow-up (*ref.: 18–24* *years*)						
Total	Random	2	0.81	(0.72, 0.93)	55	NA
26–52 weeks, *Univariate*	Random	2	0.81	(0.72, 0.93)	55	NA
Subgroup: 56–64 years by follow-up (*ref.: 18–24* *years*)						
Total	Random	2	0.55	(0.44, 0.68)	83	NA
26–52 weeks, *Univariate*	Random	2	0.55	(0.44, 0.68)	83	NA
Subgroup: 40–65 years by follow-up (*ref.: 18–39* *years*)						
Total	Random	2	0.73	(0.64, 0.82)	73	NA
52 weeks, *Univariate*	Random	2	0.73	(0.64, 0.82)	73	NA
SEX						
Subgroup: sex by follow-up (*ref.: male*)						
Total	Random	2	1.00	(0.98, 1.02)	0	NA
4 weeks, *Multivariate*	Random	2	1.00	(0.98, 1.02)	0	NA
PSYCHIATRIC COMORBIDITY						
Subgroup by psychiatric comorbidity (*26* *weeks*) (*ref.: no*)						
Total	Random	3	0.99*	(0.87, 1.11)	93	91.1 (<0.00001)
Alcohol-related disorders, *Multivariate*	Random	2	1.16*	(1.08, 1.23)	0	NA
Sleep disorder, *Univariate*	Random	2	0.85*	(0.76, 0.95)	88	NA
Substance-related disorder, *Preferably univariate*	Random	2	0.98	(0.87, 1.11)	0	NA

Note: HR: hazard ratio; NA: not applicable; OR: odds ratio; Random: random effect; ref.: reference.

*HR.

#### 3.3.1 Adherence rates

In relation to the initiation rates, 36.04% of the patients who were prescribed an AD treatment did not start it (Holvast et al., 2019).

Regarding the implementation phase, only 14% of the patients complied with the pharmacological treatment for up to 3 months, a similar percentage of the patients complied between months three and six (13%). In addition, a slight increase to 29% was observed between months six and nine and this increased to 57% between months nine and twelve, with this being the moment of greatest compliance. Once 1 year of treatment had been completed, the percentage of patients began to decrease to 31%, returning to a similar rate to the initial rates after 1 year (16%).

Regarding the discontinuation phase, 31% of the patients who were prescribed an AD treatment completed their treatment between the first three and 6 months of treatment and this rose to 52% of the patients at 40–52 weeks.

#### 3.3.2 Predictors of the initiation phase of adherence

The results relating to the predictive factors of adherence during the initiation phase (Holvast et al., 2019), are described below.

When considering the different AD treatments together, no predictive factor (physical comorbidities, chronic drug use, age, sex, and socioeconomic status) was associated with non-initiation. However, specifically for the SSRIs, it was observed that not starting pharmacological therapy was associated with a higher socioeconomic level (OR = 1.13; 95% CI: 1.01, 1.27). Regarding other types of ADs (N06AF -monoamine oxidase inhibitors- and N06AX—other ADs), being a woman was associated with the risk of non-initiation (OR = 7.89; 95% CI: 1.50, 41.68), however, the increase in the number of medications for chronic use decreased this risk (OR = 0.65; 95% CI: 0.46–0.90).

#### 3.3.3 Predictors of the implementation phase of adherence

The results obtained relating to the predictive factors of adherence during the implementation phase are described below.

##### 3.3.3.1 Age

Five studies (Akincigil et al., 2007; Chen et al., 2010; Liu et al., 2010; 2011; Wu et al., 2012) provided synthesizable data on the predictor variable age through an MA.

Between the first 12–16 weeks, patients older than 65 years of age (OR = 2.23; 95% CI: 1.61, 3.10; I^2^ = 13%; k = 2), patients between the ages of 35–49 (OR_35-49_ = 1.51; 95% CI: 1.23, 1.85; I^2^ = 58%; k = 2) and between 50 and 65 (OR_50-65_ = 1.85; 95% CI: 1.05, 3.26; I^2^ = 93%; k = 2) had better adherence rates when compared to younger ones (18–34 years).

Between 33 and 39 weeks, patients older than 65 years did not present better adherence rates (vs. 18–34 years) (OR = 1.32; 95% CI: 0.88, 1.97; I^2^ = 0%; k = 2). However, patients aged 35–49 and 50–65 did maintain better adherence rates than younger patients (OR_35-49_ = 1.35; 95% CI: 1.11, 1.63; I^2^ = 0%; k = 2; OR_50- 65_ = 1.65; 95% CI: 1.31, 2.09; I^2^ = 4%; k = 2).

Between 39 and 52 weeks, middle-aged patients (50–65 years of age) when compared to younger ones (18–35 years), continued to present better adherence rates (OR = 2.03; 95% CI: 1.91, 2.15; I^2^ = 0%; k = 6). However, this effect disappeared in patients aged between 25 and 50 years (OR = 1.02; 95% CI: 0.69, 1.52; I^2^ = 67%; k = 2).

From week 39 to 52, patients between 35 and 49 years, compared to those between 18 and 34, showed a better treatment adherence rate (OR = 1.49; 95% CI: 1.38, 1.62; I^2^ = 15%; k = 3).

Additionally, data were available in 17 studies assessing the implementation phase, but could not be synthesized through an MA. Four studies (McLaughlin et al., 2007; Stang et al., 2007; Yen et al., 2009; Yau et al., 2014) that evaluated age (continuous) as a predictor found that the treatment adherence rate was higher among older individuals. Among the studies comparing different age groups, one study indicated a higher likelihood of treatment adherence among individuals between the ages of 35–65 compared to younger individuals (Shin et al., 2022). Three other studies also provided similar data, indicating that elderly patients have better adherence rates than patients under 60 years (Sirey et al., 2001), 45 years (Merrick et al., 2012) or 35 years (Kogut et al., 2016) of age. In the remaining nine studies (Lin et al., 1995; Lin et al., 2011; Cohen et al., 2004; Keeley et al., 2007; ten Doesschate et al., 2009; Wu and Davis-Ajami (2014); Gerlach et al., 2017; Holvast et al., 2019; Bhattacharjee et al., 2020), no significant differences of adherence during the implementation phase were found among different age groups.

##### 3.3.3.2 Sex

The MA incorporated the findings from 11 studies (Donohue et al., 2004; Akincigil et al., 2007; Stang et al., 2007; Yen et al., 2009; Chen et al., 2010; Liu et al., 2011; Merrick et al., 2012; Yau et al., 2014; Kogut et al., 2016; Gerlach et al., 2019; Holvast et al., 2019).

Women adhered better to treatment than men between weeks 12 and 16 (OR = 1.10; IC95%: 1.01, 1.20; I^2^ = 0%; k = 4), although as the weeks progressed (26–39 and 52 weeks), this effect subsided (OR_26-39_ = 1.10; 95% CI: 0.98, 1.24; I^2^ = 47%; k = 5; OR_52_ = 1.05; 95% CI: 1.00, 1.10; I^2^ = 0%; k = 4).

Eleven additional studies could not be included in the MA. One of them (Shin et al., 2022) reported that female individuals presented a better treatment adherence rate than male individuals. The rest of the studies (Lin et al., 1995; Lin et al., 2011; Cohen et al., 2004; Keeley et al., 2007; McLaughlin et al., 2007; ten Doesschate et al., 2009; Wu et al., 2012; Kales et al., 2013; Gerlach et al., 2017; Bhattacharjee et al., 2020) did not find significant differences in adherence rates by sex.

##### 3.3.3.3 Ethnicity

The MA incorporated the findings from five studies (Merrick et al., 2012; Wu et al., 2012; Kales et al., 2013; Kales et al., 2016; Gerlach et al., 2019).

White patients had higher treatment adherence rates than African-Americans at both 16 and 52 weeks (OR_16_ = 2.67; 95% CI: 1.86, 3.83; I^2^ = 0%; k = 3; OR_52_ = 1.85; 95% CI:1.25, 2.74; I^2^ = 37%; k = 2).

Additionally, data from five studies (Keeley et al., 2007; Lin et al., 2011; Wu and Davis-Ajami (2014); Gerlach et al., 2017; Bhattacharjee et al., 2020) could not be synthesized through an MA. Only one study (Keeley et al., 2007) did not find differences in adherence rates between Caucasian, African-Americans and Hispanic patients. Among the remaining studies, two of them reported a higher likelihood of treatment adherence in the implementation phase among white/Caucasian individuals compared to African-American or non-white/non-Caucasian patients (Wu et al., 2012; Wu and Davis-Ajami, 2014). In one study (Bhattacharjee et al., 2020), white patients showed better adherence compared to non-white race and Hispanic patients. The remaining study (Lin et al., 2011) provided similar results, finding that Hispanic patients have lower levels of adherence compared to Caucasian or other ethnic patients. However, this study found no differences between Hispanics and African-Americans.

##### 3.3.3.4 Education

Five studies evaluated the impact of education in the implementation phase of adherence (Lin et al., 1995; Keeley et al., 2007; ten Doesschate et al., 2009; Yen et al., 2009; Gerlach et al., 2019).

Two of the studies reported sufficient data on the influence of education on the implementation phase of adherence between week 54 and 104 to be included in an MA (ten Doesschate et al., 2009; Gerlach et al., 2019). However, the analysis showed very high heterogeneity rates (I^2^ = 76%), and as such the pooled data are not presented.

All studies (Lin et al., 1995; Keeley et al., 2007; ten Doesschate et al., 2009; Yen et al., 2009; Gerlach et al., 2019) reported a non-significant effect of years of education on the implementation phase of adherence.

##### 3.3.3.5 Civil status

Four studies (ten Doesschate et al., 2009; Holma et al., 2010; Kales et al., 2016; Gerlach et al., 2019) analyzed the effect of civil status on adherence. Three of them (Holma et al., 2010; Kales et al., 2016; Gerlach et al., 2019) found a significant result. Individuals with spouse, partner or not living alone presented higher rates of adherence than individuals without spouse, partner or living alone. Due to disparities in follow-up, the results presented could not be effectively synthesized using MA.

Please refer to Supplementary Table S3 in the Supplementary Material to access the data from individual studies.

##### 3.3.3.6 Income

Four studies (Akincigil et al., 2007; Holvast et al., 2019; Nam-Ju and Yeon-Pyo, 2020) examined the influence of income on adherence during the implementation phase. However, an MA could not be conducted due to the wide variability in categorizing income levels. Despite this limitation, three of them (Akincigil et al., 2007; Holvast et al., 2019; Nam-Ju and Yeon-Pyo, 2020) consistently found that adherence was lower among individuals with lower incomes compared to those with higher incomes. The remaining study (Lin et al., 2011) reported a non-significant result.

##### 3.3.3.7 Medical comorbidities

MA could not be performed. Eight studies explored the impact of medical comorbidities on adherence (Akincigil et al., 2007; Keeley et al., 2007; Liu et al., 2011; Merrick et al., 2012; Wu et al., 2012; Kales et al., 2016; Gerlach et al., 2019; Bhattacharjee et al., 2020).

Three studies (Merrick et al., 2012; Kales et al., 2016; Gerlach et al., 2019) analyzed the impact of medical comorbidities using the Charlson Comorbidity Index (CCI) on adherence. The CCI is a medical tool for assessing both the number and severity of comorbid diseases, which helps to predict mortality (Charlson et al., 1987). Of the three studies, two (Kales et al., 2016; Gerlach et al., 2019) found that individuals with a CCI score greater than zero had a higher rate of adherence during the implementation phase in contrast to individuals with other CCI scores. However, the remaining study (Merrick et al., 2012) did not find significant differences in adherence between individuals with a CCI score of two and those with a CCI score between 0 and 1. Additionally, one study used the Elixhauser Comorbidity Index (Wu et al., 2012). The said study found higher levels of adherence in patients who scored 2 or more on the Elixhauser Comorbidity Index compared to those who scored lower. However, patients who scored 1 did not differ from those who scored 0.

Two studies analyzed the impact of comorbid chronic pain conditions (such as low back pain, migraines, fibromyalgia, or headaches) on treatment adherence and found significant differences. One study (Liu et al., 2011) indicated that individuals with headaches, low back pain, or fibromyalgia had a lower likelihood of treatment adherence compared to those without these conditions. In the other study (Akincigil et al., 2007), patients with headaches or migraines were less likely to be adherent at 16 weeks, although this difference was not observed at the 33-week follow-up.

One study (Bhattacharjee et al., 2020) reported that people with Parkinson’s disease had a slightly higher probability of treatment adherence compared to people without Parkinson’s, Additionally, individuals with cardiovascular disease and diabetes showed a lower probability of adherence compared to those without these health conditions at 16 weeks, but this effect was not observed at the 33-week follow-up (Akincigil et al., 2007).

##### 3.3.3.8 Psychiatric comorbidities

The MA incorporated the findings from four studies with regard to anxiety comorbidity. Presenting this type of disorder simultaneously with depression, between weeks 12–16 and 33–39, did not influence the rates of adherence to pharmacological treatment for depression (OR_12-16_ = 1.02; 95% CI: 0.90, 1.15; I^2^ = 0%; k = 2; OR_33-39_ = 1.04; 95% CI: 0.87, 1.24; I^2^ = 0%; k = 2). However, after week 52, suffering from anxiety at the same time as depression increased adherence rates (OR = 1.50; 95% CI: 1.25, 1.81; I^2^ = 0%; k = 2).

Additional data related to psychiatric comorbidities from five studies could not be included in the MA. Three studies (Keeley et al., 2007; Lin et al., 2011; Gerlach et al., 2019) revealed that the presence of several psychiatric comorbidities (i.e., anxiety disorder, somatoform complaints, substance use or post-traumatic stress disorders) did not significantly impact the implementation process. Furthermore, one study (Chen et al., 2010) reported that patients with a comorbid substance use disorder had a lower probability of adherence at 12 weeks, but not at 39 weeks of follow-up. The last study (Liu et al., 2011) found results consistent with the ones mentioned earlier. The finding was that patients with alcohol related-disorders and the use or abuse of substances had worse adherence rates compared to those who do not suffer from them. Conversely, patients with hypersomnia had a higher likelihood of continuing to adhere to treatment. On the contrary, patients with anxiety or comorbid fibromyalgia had worse implementation rates.

##### 3.3.3.9 Diagnostic subtype previous episodes and severity

Threes studies (Cohen et al., 2004; Donohue et al., 2004; Yen et al., 2009) investigated the impact of diagnostic subtype on treatment adherence and did not observe any significant differences.

A single study (Cohen et al., 2004) investigated the impact of previous episodes on adherence and did not observe any significant differences.

MA could not be performed. Four studies (Lin et al., 1995; Sirey et al., 2001; Cohen et al., 2004; Merrick et al., 2012) investigated the impact of severity on treatment implementation and did not observe any significant differences.

#### 3.3.4 Predictors of the discontinuation phase of adherence

The results obtained relating to the predictors of adherence during the discontinuation phase (non-persistence) are described below.

##### 3.3.4.1 Age

The MA incorporated the findings from six studies (Goethe et al., 2007; Liu et al., 2010; 2011; Milea et al., 2010; Woolley et al., 2010; Vlahiotis et al., 2011).

The increase in age generated a slight decrease in discontinuation rates at 12 weeks (OR = 0.98; 95% CI: 0.97, 0.99; I^2^ = 0%; k = 2).

Between weeks 26–52, patients aged 25–40 (OR = 0.81; 95% CI: 0.72, 0.93; I^2^ = 55%; k = 2) and 56–64 (OR = 0.55; 95% CI: 0.44, 0.68; I^2^ = 83%; k = 2) presented lower rates of discontinuation of AD treatment compared to those aged 18–24.

At week 52, only patients aged 40–64 (vs. 18–39) maintained lower discontinuation rates (OR = 0.73; 95% CI: 0.64, 0.82; I^2^ = 73%; k = 2) compared to those aged 18–39.

Data from another eight studies could not be synthesized using MA. In five studies, no significant differences were found among different age groups (Keeley et al., 2000; 2007; Demyttenaere et al., 2001; Olfson et al., 2006; Noh et al., 2020). However, two studies (Ereshefsky et al., 2010; Wu et al., 2013) reported that there was a lower likelihood of treatment discontinuation among individuals older than 35 and 45 years compared to younger individuals. A similar result was found in another study that evaluated age as a continuous variable (Hung et al., 2011), where a greater age independently predicted a lower risk of early discontinuation. In contrast, one study (Hung et al., 2011) found the opposite effect, where older people were at a higher risk of discontinuation.

Please refer to Supplementary Table S5 in the Supplementary Material to access the data from individual studies.

##### 3.3.4.2 Sex

The MA investigating the influence of sex on adherence during the discontinuation phase included findings from two studies (Olfson et al., 2006; Milea et al., 2010). Sex did not affect the discontinuation rates at 4 weeks (OR = 1.00; 95% CI: 0.98, 1.02; I^2^ = 0%; k = 2).

Another seven studies (Demyttenaere et al., 2001; Goethe et al., 2007; Keeley et al., 2007; Hung et al., 2011; Vlahiotis et al., 2011; Wu et al., 2013; Holvast et al., 2019) were not included in the MA. Among them, two studies (Goethe et al., 2007; Vlahiotis et al., 2011) consistently reported that men had a significantly higher risk of discontinuation than women. Milea et al. (2010) reported a similar result, although this was observed as only a trend. In contrast, one study (Wu et al., 2013) reported that men presented a significantly lower risk than women. The remaining studies found no significant impact of gender on discontinuation.

##### 3.3.4.3 Ethnicity

MA could not be performed. Three studies examined the impact of ethnicity on discontinuation (Keeley et al., 2000; Keeley et al., 2007; Olfson et al., 2006). Two of them did not observe any significant differences (Keeley et al., 2000;Keeley et al., 2007), while another study found that Hispanic patients had a higher rate of treatment discontinuation than non-Hispanic patients (Olfson et al., 2006).

##### 3.3.4.4 Education

Two of the three studies (Olfson et al., 2006; Woolley et al., 2010) provided sufficient data regarding the impact of educational level on discontinuation within 12 weeks to conduct an MA. However, the analysis revealed a high level of heterogeneity (I^2^ = 83), and as such pooled data are not presented.

One study reported a higher rate of treatment discontinuation among patients with less than 12 years of formal education. However, the remaining two studies (Keeley et al., 2007; Woolley et al., 2010) did not observe significant differences.

##### 3.3.4.5 Civil status

MA could not be performed. The civil status of the patients did not influence the discontinuation rates at 4 weeks (Olfson et al., 2006).

##### 3.3.4.6 Income

MA could not be performed. Two studies (Olfson et al., 2006; Holvast et al., 2019) investigated the impact of income level on discontinuation. Only Olfson et al. (2006) observed that individuals with a low income had a significantly higher rate of treatment discontinuation compared to those with a high income.

##### 3.3.4.7 Medical comorbidities

MA could not be performed. Six studies (Keeley et al., 2000; Keeley et al., 2007; ten Doesschate et al., 2009; Vlahiotis et al., 2011; Wu et al., 2013; Wu and Davis-Ajami (2014)) explored the impact of medical comorbidities on discontinuation and did not observe significant differences.

Three studies suggested that the presence of various medical comorbidities could actually lead to a decreased risk of discontinuation of AD treatment. For instance, one study (Noh et al., 2022) reported that a reduced likelihood of AD discontinuation was found in women with a higher obstetric comorbidity index or the presence of cardiovascular disease. Similarly, another investigation highlighted the impact of somatic comorbidities, including hypertension, lipid metabolic disorder, and diabetes, which were associated with a lower occurrence of treatment discontinuation (Milea et al., 2010). Furthermore, in one study (Hung et al., 2011), patients with migraines were less inclined to discontinue treatment when compared to those without migraine conditions.

##### 3.3.4.8 Psychiatric comorbidities

The MA included data from three studies (Ereshefsky et al., 2010; Wu et al., 2013; Noh et al., 2022) concerning psychiatric comorbidities. Overall, the presence of a psychiatric comorbidity did not significantly affect adherence rates (HR = 0.99; 95% CI 0.87, 1.13; I^2^ = 93%; k = 3).

However, when examining specific comorbidities, it was found that patients with alcohol-related disorders presented worse adherence rates to AD treatment at 26 weeks (HR = 1.16; 95% CI: 1.08, 1.23; I^2^ = 0%; k = 2) (Ereshefsky et al., 2010; Wu et al., 2013). Conversely, the presence of sleep disorders did not influence adherence rates at 26 weeks (HR = 0.85; 95% CI: 0.76, 0.95; I^2^ = 88%; k = 2) (Wu et al., 2013; Noh et al., 2022), nor did substance abuse-related disorders (HR = 0.98; 95% CI: 0.87, 1.11; I^2^ = 0%; k = 2) (Wu et al., 2013; Noh et al., 2022).

Additional data relating to psychiatric comorbidities from nine studies could not be synthesized using MA. One study (Liu et al., 2011) reported similar findings to the previous ones, suggesting that patients with alcohol related-disorders and the use or abuse of substances had worse adherence rates compared to those without these conditions at 52 weeks. However, patients with hypersomnia were more likely to continue complying with treatment. Another study (Holvast et al., 2019) found that the presence of psychological comorbidity was not associated with discontinuation. However, sensitivity analysis for different types of ADs revealed an association between the psychological comorbidity and discontinuation of SSRIs.

Concerning anxiety comorbidity, there is some variation in the findings. In one study (Wu et al., 2013), patients with anxiety comorbidity were less likely to discontinue AD treatment at 26 weeks. Conversely, another study (Vlahiotis et al., 2011) suggested that anxiety disorders often led to increased discontinuation. However, two other studies (Goethe et al., 2007; Wu et al., 2012) did not find this association at 12 and 52 weeks.

Different studies reported that comorbidities such as panic/agoraphobia or post-traumatic stress disorder (Hung et al., 2011), or sleep disorder and anxiety/stress related disorder (Noh et al., 2022), were associated with reduced treatment discontinuation rates. However, the presence of a psychosomatic comorbidity was associated with an increased discontinuation rate (Milea et al., 2010).

Finally, two studies (Keeley et al., 2007; Noh et al., 2020) found that the presence of somatoform complaints, mood disorders, eating disorders or personality disorders did not significantly affect the AD discontinuation process.

##### 3.3.4.9 Previous episodes and severity

Two studies (ten Doesschate et al., 2009; Wu and Davis-Ajami, 2014) investigated the impact of previous episodes on discontinuation and did not observe any significant differences.

MA could not be performed. Two studies (ten Doesschate et al., 2009; Hung et al., 2011) explored the relation between depression severity and discontinuation. A single study (Hung et al., 2011) found that patients with chronic depression were less likely to discontinue treatment. Conversely, another study (ten Doesschate et al., 2009) did not identify any significant differences.

Finally, with the data reported in the included studies, it was not possible to synthesize the relationship between cognitive impairment, and perceived health or health-related quality of life with adherence (for more information on the results obtained in the included studies see Supplementary Table S6).

## 4 Discussion

The main objective of this SR was to evaluate the possible sociodemographic and clinical predictive factors that influence adherence to AD treatment in adult patients diagnosed with a depressive disorder.

The data obtained in this SR show worrying rates of adherence to pharmacological treatment in the three phases, initiation, implementation and discontinuation (Vrijens et al., 2012). Specifically, non-adherence rates in the first months of therapy exceed 80%, which places this problem in a more unfavorable scenario than those reported in previous studies, which reported values close to 50% (Sansone and Sansone, 2012). These high rates of non-adherence may be influenced by factors such as the side effects of medication, especially given that this occurs in the early weeks of AD treatment. This underscores the need for a professional approach concerning the experience of the disease and the treatment (feelings, ideas, function and expectations) to adequately manage the condition and improve therapeutic adherence. This is particularly important in scenarios where pharmacological therapy is the only viable option for the patient (Samalin et al., 2018; González de León et al., 2022).

In relation to the predictive factors, advanced age, was found to be a predictor of good adherence in both the implementation phase and in the discontinuation phase, which is consistent with the literature (Rivero-Santana et al., 2013; Holbrook et al., 2021). However, in the present SR, this effect was maintained over time in middle-aged people (35–65 years), while it was less evident in older people (>65 years). In the latter population group, the use of patient reminders or alerts could play an important role in reducing involuntary lack of adherence (Hamine et al., 2015; González de León et al., 2021). Additionally, it is important to consider the role of patient’s beliefs and preferences about medication at the start of treatment, as well as patient preferences about treatment, as they may be correlated with therapeutic efficacy and adherence, especially in younger patients (Horne et al., 2013; Kong et al., 2021).

In the present SR, it was observed that being female was associated with better adherence rates during the first weeks of treatment, but correlated with the risk of SSRIs non-initiation. However, as treatment time progresses, this association became less conclusive. Previous studies similarly reported a better adherence rate between female patients nevertheless, this finding could not be consistently confirmed due to many studies not yielding statistically significant results (Rivero-Santana et al., 2013).

On the other hand, white patients showed better levels of adherence compared to Afro-American or Hispanic patients, consistent with some previous literature (Rivero-Santana et al., 2013) that mainly pointed to the age and ethnicity of the patients as the most consistent factors influencing non-compliance with treatment. This finding contrasts with the results of the SR of Holbrook et al. (2021), where they did not consider ethnicity as a predictive factor. This controversial relationship may be mediated by confounders such as economic resources, educational level or healthcare access, as in other outcomes in depression (Finegan et al., 2018). Therefore, future studies designed to corroborate these results are needed.

Although having a low educational level has often been considered a potential risk factor for poor adherence, as people with less education may have more difficulty understanding treatment regimens, medical recommendations, or the nature of their disease; the educational level of the patients did not influence treatment adherence rates. This finding is consistent with previous studies (Burra et al., 2007; Rivero-Santana et al., 2013; Roca et al., 2013).

Another possible association, in line with previous research on chronic conditions, was found between marital or cohabitation status and medication adherence. Studies conducted on other chronic diseases have found a relationship between marital status and adherence, with a greater adherence in those people who were in a relationship (Trivedi et al., 2008; Wu et al., 2012).

Marital or cohabitation status may also be associated with medication adherence. Research on various chronic conditions has suggested that individuals in relationships tend to present better adherence (Trivedi et al., 2008; Wu et al., 2012).

Previous studies suggest that socioeconomically disadvantaged individuals, characterized by factors such as low income, unemployment, financial struggles, lack of homeownership, or limited formal education, have poorer prognoses regardless of the type of treatment they receive and the severity of depression (Buckman et al., 2022). In the present SR, low income appears to have a negative impact on both the initiation of SSRI treatment and adherence levels to AD therapy, and possibly on discontinuation rates, which could be influencing the poor progression of the disease.

Regarding psychiatric comorbidities, the findings in the present SR showed varied results. During the first weeks, the presence of anxiety disorders did not seem to influence adherence. However, over time, the absence of anxiety disorders was associated with better adherence rates. Consistent with previous research on chronic conditions (Grodensky et al., 2012), it appears that patients with comorbid depression and alcohol abuse disorder may present reduced adherence to treatment. Nevertheless, no significant results were found for sleep disorders and substance abuse-related disorders. These results highlight a potentially important gap in the evidence about the effect of psychiatric comorbidities on medication adherence.

On the other hand, patients with a higher medical comorbidity index score showed better adherence during the implementation phase. However, contrary to expectations, studies examining the role of comorbid chronic pain found lower adherence rates among patients with these conditions (Akincigil et al., 2007; Liu et al., 2011). Both of these studies reported a similar difference in the adherence ratio between patients with or without chronic pain of around 4%, although this was relatively small, it is significant. Non-adherence to prescribed analgesic medication in chronic pain is quite common, influenced by factors such as polymedication and concerns about pain medication, which are commonly associated with non-adherence in this condition (Timmerman et al., 2019). These aspects might also affect adherence to antidepressants. This finding emphasizes the need for further studies to draw more robust conclusions. Furthermore, it is necessary to understand how these findings translate into real clinical practice situations.

For individuals dealing with comorbid conditions, simplifying the medication regimen may prove beneficial. As seen in prior literature (Rivero-Santana et al., 2013), medical comorbidities have been shown to have significant associations with both positive and negative adherence outcomes in the studies examined here. Patients coping with multiple health conditions may develop a more profound understanding of medication management. However, when combined with other factors like limited education, or incomplete or unclear physician instructions, this can lead to a complex treatment regimen that complicates adherence. It is also important to address patients’ myths and beliefs with scientific information and explanations (Marasine and Sanki, 2021). This combined approach could help improve adherence in patients with comorbidities and contribute to better treatment outcomes.

The evidence in the present SR suggests that the severity of depression by itself does not significantly predict adherence, which is consistent with previous SR (Rivero-Santana et al., 2013). However, older patients experiencing severe and persistent depressive symptoms are more inclined to tend to perceive medication as a necessary treatment for their condition. Conversely, in younger patients with severe initial depression, the dropout rate from pharmacotherapy tends to be higher (Aikens et al., 2008). Data from databases usually lack essential information, such as disease severity and prior episode history, which is required to understand the disease. The loss of information derived from incomplete coding during the diagnosis process and its subsequent updating complicates the analysis of possible relationships between these factors and treatment adherence (Donohue et al., 2004). Hence, additional scientific evidence is needed to shed light on what is happening with the more purely clinical characteristics of these patients.

The findings here suggest that depression severity alone might not significantly predict adherence, which is consistent with previous systematic reviews (Rivero-Santana et al., 2013). Nevertheless, older individuals experiencing severe and persistent depressive symptoms are more inclined to view medication as a necessary treatment for their condition. Conversely, in younger individuals with severe initial depression, the dropout rate from pharmacotherapy tends to be higher (Aikens et al., 2008).

The study of all the potential predictive factors influencing the decision-making about starting (or not), maintaining (or not), and discontinuing (or not) the treatment is necessary to enhance the existing theoretical models and develop more precise and adjusted interventions for different subgroups of the population. The identification of these predictors of adherence holds significant value for primary care and mental health professionals in their everyday clinical practice. It enables them to identify patients who may be at a higher risk of non-adherence, allowing for the implementation of targeted interventions for these individuals. By doing so, it becomes possible to enhance clinical outcomes in the recovery process and optimize the utilization of public health resources efficiently. This proactive approach can ultimately lead to better patient outcomes and a more effective allocation of healthcare resources.

### 4.1 Strengths and limitations

This SR has a series of strengths, namely, 1) it is the most extensive work to date in relation to the number of participants, which in addition to incorporating MA, 2) used a transparent and rigorous methodology according to the SR and MA standards, and 3) each of the steps is explained in detail, as well as providing all the necessary data to be able to replicate this SR.

With regard to the weaknesses of this study, the following should be mentioned: 1) despite conducting an exhaustive bibliographic search in the main databases of indexed journals, there may be studies not included in these databases that have therefore been left out of this SR, 2) only studies published in English and/or Spanish were taken into account, 3) a large number of the studies presented a high overall risk of bias, which limits the certainty of the evidence, 4) there was heterogeneity between the selected studies, especially in how and when adherence is assessed, and in the definition and categorization of the predictors, which, in some cases, has meant that it has not been possible to obtain an estimate of the effect of some of the predictive factors and, 5) despite ongoing consensus efforts, the considerable variability in defining adherence and its phases has posed a challenge to comparing studies.

Other limitations, mainly concern the low number of studies per predictor factor, are 6) the absence of a meta-regression analysis, 7) the lack of sensitivity analysis and the adherence measurement method in included studies. Adherence is a multifactorial phenomenon, and as such, it should ideally be evaluated from various perspectives. Relying solely on a single measurement method, whether objective or subjective, through the use of validated scales, might prove insufficient. In the future, studies should incorporate the gold standard—electronic monitoring—(Hess et al., 2006) and, when the reference standard is not used, two evaluation methods should be applied: one using objective measures and the other subjective measures of adherence (Sajatovic et al., 2010).

Finally, despite the efforts, the profile obtained, due to its restriction to unmodified predictors of adherence, is limited in its usefulness in clinical practice for effectively identifying a well-defined non-adherence patient profile.

### 4.2 Conclusion

According to the results obtained here, middle-aged, elderly and Caucasian participants have higher rates of adherence, although time determines whether these rates are maintained in older patients. Despite finding data that support age and ethnicity as predictors of pharmacological adherence, further studies of a higher methodological quality that can obtain more data, but, above all, that explore other possible factors that may influence adherence are recommended.

## Data Availability

The original contributions presented in the study are included in the article/Supplementary Material, further inquiries can be directed to the corresponding author.
