# Supplementary material for: Sociodemographic and clinical predictors of adherence to antidepressants in depressive disorders: a systematic review with a meta-analysis
Source: Front Pharmacol. 2024 Jan 22;15:1327155. doi: 10.3389/fphar.2024.1327155 (PMC10839896; doi:10.3389/fphar.2024.1327155)

Supplementary Material

Supplementary Table 1. Search Strategies

| Medline |
| --- |
| 1. Depression/ 2. Depressive Disorder/ 3. major/ 4. Mood Disorders/ 5. (depress* or mood? or affective disorder* or affective symptom* or depress* disorder* or 6. 1 or 2 or 3 or 4 or 5 7. Bipolar Disorders/ 8. Psychotic Disorders/ 9. (bipolar or bipolar disorder*).ti,ab. 10. (mania or manic*).ti,ab. 11. (Schizoaffective or schizo-affective).ti,ab. 12. 7 or 8 or 9 or 10 or 11 13. 6 not 12 14. Patient Compliance/ 15. Medication Adherence/ 16. Treatment Refusal/ 17. (adhere$ or non adherence or nonadherence or non-adherence or complian$ or non 18. 14 or 15 or 16 or 17 19. Antidepressive Agents/ 20. exp Neurotransmitter Uptake Inhibitors/ 21. exp Monoamine Oxidase Inhibitors/ 22. (Agomelatine or Alnespirone or Amoxapine or Amersergide or Amfebutamone or Amiflamine or Amineptine or Amitriptylin* or Amitriptylinoxide or Amoxapine or Aripiprazole or Atomoxetine or Tomoxetine or Befloxatone or Benactyzine or Binospirone or Brofaromine or Bupropion or Butriptylin* or Cianopramine or Cilobamine or Cimoxatone or Citalopram or Chlorimipramin* or Clomipramin* or Chlomipramin* or Clorimipramine or Clorgyline or Clovoxamine or Dapoxetine or Deanol or Dibenzepin or Demexiptilin* or Deprenyl or Desipramine or Desvenlafaxine or Dibenzepin or Dimetacrin* or Dosulepin* or Dothiepin or Doxepin* or Duloxetine or DVS 233 or Enilospirone or Eptapirone or Escitalopram or Esketamine or Etoperidone or Femoxetine or Fenelzine or Fluotracen or Fluoxetine or Fluparoxan or Furazolidone or Fluvoxamine).ti,ab. 23. (Harmaline or Harmine or Hyperforin or Hypericum or John* Wort or Idazoxan or 24. (Opipramol or Oxaflozane or Paroxetine or Phenelzine or Pheniprazine or Pipofezin* or 25. ((serotonin or norepinephrine or noradrenaline or nor epinephrine or nor adrenaline or 26. (psychotropic* or antidepress* or anti depress* o noradrenerg* or antiadrenergic or anti 27. ((serotonin or monoamine oxidase or MAO) adj2 inhibit*).ti,ab. 28. 19 or 20 or 21 or 22 or 23 or 24 or 25 or 26 or 27 29. 13 and 18 and 28 30. limit 29 to humans 31. limit 30 to (english or spanish) 32. Epidemiologic studies/ 33. exp case control studies/ 34. exp cohort studies/ 35. Case control.tw. 36. (cohort adj (study or studies)).tw. 37. Cohort analy$.tw. 38. (Follow up adj (study or studies)).tw. 39. (observational adj (study or studies)).tw. 40. Longitudinal.tw. 41. Retrospective.tw. 42. Cross sectional.tw. 43. Cross-sectional studies/ 44. 32 or 33 or 34 or 35 or 36 or 37 or 38 or 39 or 40 or 41 or 42 or 43 45. 31 and 44 |
| Embase |
| 1. No. 2. Query 3. Results 4. #31 AND #44 5. #32 OR #33 OR #34 OR #35 OR #36 OR #37 OR #38 OR #39 OR #40 OR #41 OR #42 OR #43 6. 'cross-sectional':de 7. 'cross-sectional study':de 8. retrospective:de 9. longitudinal:de 10. (observational NEAR/1 (study OR studies)):ti,ab,de 11. follow:ti,ab,de AND ((up NEAR/1 (study OR studies)):ti,ab,de) 12. 'cohort analysis':ti,ab,de 13. (cohort NEAR/1 (study OR studies)):ti,ab,de 14. 'case control':de 15. 'cohort analysis'/exp 16. 'case control study'/exp 17. 'epidemiology'/de 18. #12 AND #18 AND #28 AND [humans]/lim AND ([english]/lim OR [spanish]/lim) 19. #12 AND #18 AND #28 AND [humans]/lim 20. #12 AND #18 AND #28 21. #19 OR #20 OR #21 OR #22 OR #23 OR #24 OR #25 OR #26 OR #27 22. ((serotonin OR 'monoamine oxidase' OR mao) NEAR/2 inhibit*):ti,ab 23. (psychotropic*:ti,ab OR antidepress*:ti,ab OR 'anti depress*':ti,ab) AND o:ti,ab AND noradrenerg*:ti,ab OR antiadrenergic:ti,ab OR 'anti adrenergic':ti,ab OR ssri*:ti,ab OR snri*:ti,ab OR maoi*:ti,ab OR tricyclic*:ti,ab OR nari:ti,ab OR naris:ti,ab OR ndir*:ti,ab OR sari:ti,ab OR saris:ti,ab OR nassa*:ti,ab 24. ((serotonin OR norepinephrine OR noradrenaline OR 'nor epinephrine' OR 'nor adrenaline' OR neurotransmitt* OR dopamine*) NEAR/3 (uptake OR reuptake OR 're-uptake')):ti,ab 25. opipramol:ti,ab OR oxaflozane:ti,ab OR paroxetine:ti,ab OR phenelzine:ti,ab OR pheniprazine:ti,ab OR pipofezin*:ti,ab OR pirandamine:ti,ab OR piribedil:ti,ab OR pirlindole:ti,ab OR pivagabine:ti,ab OR pizotyline:ti,ab OR propizepine:ti,ab OR protriptylin*:ti,ab OR pertofrane:ti,ab OR quinupramine:ti,ab OR quipazine:ti,ab OR reboxetine:ti,ab OR ritanserin:ti,ab OR rolipram:ti,ab OR scopolamine:ti,ab OR selegiline:ti,ab OR sertraline:ti,ab OR setiptiline:ti,ab OR teciptiline:ti,ab OR tandospirone:ti,ab OR tetrindole:ti,ab OR thiazesim:ti,ab OR thozalinone:ti,ab OR tianeptin*:ti,ab OR toloxatone:ti,ab OR tranylcypromine:ti,ab OR trazodone:ti,ab OR trimipramine:ti,ab OR '5 hydroxytryptophan':ti,ab OR '5 ht':ti,ab OR tryptophan:ti,ab OR hydroxytryptophan:ti,ab OR venlafaxine:ti,ab OR viloxazine:ti,ab OR vilazodone:ti,ab OR viqualine:ti,ab OR vortioxetine:ti,ab OR zalospirone:ti,ab OR zimeldine:ti,ab 26. (harmaline:ti,ab OR harmine:ti,ab OR hyperforin:ti,ab OR hypericum:ti,ab OR john*:ti,ab) AND wort:ti,ab OR idazoxan:ti,ab OR imipramin*:ti,ab OR iprindole:ti,ab OR iproniazid*:ti,ab OR ipsapirone:ti,ab OR imipraminoxide:ti,ab OR isocarboxazid*:ti,ab OR lesopitron:ti,ab OR levomilnacipran:ti,ab OR lithium:ti,ab OR lofepramin*:ti,ab OR 'lu aa21004':ti,ab OR vortioxetine:ti,ab OR 'lu aa24530':ti,ab OR 'ly2216684':ti,ab OR maprotiline:ti,ab OR medifoxamine:ti,ab OR melitracen:ti,ab OR metapramine:ti,ab OR methylphenidate:ti,ab OR mianserin:ti,ab OR milnacipran:ti,ab OR minaprine:ti,ab OR mirtazapine:ti,ab OR moclobemide:ti,ab OR nefazodone:ti,ab OR nialamide:ti,ab OR nitroxazepine:ti,ab OR nomifensine:ti,ab OR norfenfluramine:ti,ab OR nortriptyline:ti,ab OR noxiptilin*:ti,ab 27. agomelatine:ti,ab OR alnespirone:ti,ab OR amersergide:ti,ab OR amfebutamone:ti,ab OR amiflamine:ti,ab OR amineptine:ti,ab OR amitriptylin*:ti,ab OR amitriptylinoxide:ti,ab OR amoxapine:ti,ab OR aripiprazole:ti,ab OR atomoxetine:ti,ab OR tomoxetine:ti,ab OR befloxatone:ti,ab OR benactyzine:ti,ab OR binospirone:ti,ab OR brofaromine:ti,ab OR bupropion:ti,ab OR butriptylin*:ti,ab OR cianopramine:ti,ab OR cilobamine:ti,ab OR cimoxatone:ti,ab OR citalopram:ti,ab OR chlorimipramin*:ti,ab OR clomipramin*:ti,ab OR chlomipramin*:ti,ab OR clorimipramine:ti,ab OR clorgyline:ti,ab OR clovoxamine:ti,ab OR dapoxetine:ti,ab OR deanol:ti,ab OR demexiptilin*:ti,ab OR deprenyl:ti,ab OR desipramine:ti,ab OR desvenlafaxine:ti,ab OR dibenzepin:ti,ab OR dimetacrin*:ti,ab OR dosulepin*:ti,ab OR dothiepin:ti,ab OR doxepin*:ti,ab OR duloxetine:ti,ab OR 'dvs 233':ti,ab OR enilospirone:ti,ab OR eptapirone:ti,ab OR escitalopram:ti,ab OR esketamine:ti,ab OR etoperidone:ti,ab OR femoxetine:ti,ab OR fenelzine:ti,ab OR fluotracen:ti,ab OR fluoxetine:ti,ab OR fluparoxan:ti,ab OR furazolidone:ti,ab OR fluvoxamine:ti,ab 28. 'monoamine oxidase inhibitor'/exp 29. 'neurotransmitter uptake inhibitor'/exp 30. 'antidepressant agent'/exp 31. #13 OR #14 OR #15 OR #16 OR #17 32. 'management adherence':ti,ab 33. adhere*:ti,ab,de OR 'non adherence':ti,ab,de OR nonadherence:ti,ab,de OR 'non-adherence':ti,ab,de OR complian*:ti,ab,de OR 'non complian*':ti,ab,de OR 'non-complian*':ti,ab,de OR persistence:ti,ab,de OR discontinus:ti,ab,de 34. 'treatment refusal' 35. 'medication compliance' 36. 'patient compliance' 37. #5 NOT #11 38. #6 OR #7 OR #8 OR #9 OR #10 39. schizoaffective:ti,ab OR 'schizo-affective':ti,ab 40. mania:ti,ab OR manic*:ti,ab 41. bipolar:ti,ab OR 'bipolar disorder*':ti,ab 42. 'psychosis' 43. 'bipolar disorder' 44. #1 OR #2 OR #3 OR #4 45. depress*:ti,ab OR mood?:ti,ab OR 'affective disorder*':ti,ab OR 'affective symptom*':ti,ab OR 'depress* disorder*':ti,ab OR dysphoria:ti,ab OR 'endogenous depression':ti,ab OR 'involutional depression':ti,ab OR 'major depression':ti,ab OR 'masked depression':ti,ab OR melancholia:ti,ab OR 'mood disorder':ti,ab OR 'organic depression':ti,ab OR 'recurrent brief depression':ti,ab OR 'anaclitic depression':ti,ab OR 'recurrent depression':ti,ab OR 'treatment resistant depression':ti,ab OR 'dysthymic disorder':ti,ab OR 'reactive depression':ti,ab OR 'agitated depression':ti,ab OR 'atypical depression':ti,ab OR sadness:ti,ab 46. 'mood disorder'/exp 47. 'major depression'/exp 48. 'depression'/exp |
| WOS |
| 1. (TI=((depress* or mood? or affective disorder* or affective symptom* or depress* disorder* or dysphoria or endogenous depression or involutional depression or major depression or masked depression or melancholia or mood disorder or organic depression or recurrent brief depression or anaclitic depression or recurrent depression or treatment resistant depression or dysthymic disorder or reactive depression or agitated depression or atypical depression or sadness))) OR AB=((depress* or mood? or affective disorder* or affective symptom* or depress* disorder* or dysphoria or endogenous depression or involutional depression or major depression or masked depression or melancholia or mood disorder or organic depression or recurrent brief depression or anaclitic depression or recurrent depression or treatment resistant depression or dysthymic disorder or reactive depression or agitated depression or atypical depression or sadness)) 2. (TI=((bipolar or bipolar disorder*))) OR AB=((bipolar or bipolar disorder*)) 3. (TI=((mania or manic*))) OR AB=((mania or manic*)) 4. (TI=((Schizoaffective or schizo-affective))) OR AB=((Schizoaffective or schizo-affective)) 5. #2 OR #3 OR #4 6. #1 not #5 7. (TI=((adhere$ or non adherence or nonadherence or non-adherence or complian$ or non complian$ or non-complian$ or persistence or discontinu$))) OR AB=((adhere$ or non adherence or nonadherence or non-adherence or complian$ or non complian$ or non-complian$ or persistence or discontinu$)) 8. (TI=((Agomelatine or Alnespirone or Amoxapine or Amersergide or Amfebutamone or Amiflamine or Amineptine or Amitriptylin* or Amitriptylinoxide or Amoxapine or Aripiprazole or Atomoxetine or Tomoxetine or Befloxatone or Benactyzine or Binospirone or Brofaromine or Bupropion or Butriptylin* or Cianopramine or Cilobamine or Cimoxatone or Citalopram or Chlorimipramin* or Clomipramin* or Chlomipramin* or Clorimipramine or Clorgyline or Clovoxamine or Dapoxetine or Deanol or Dibenzepin or Demexiptilin* or Deprenyl or Desipramine or Desvenlafaxine or Dibenzepin or Dimetacrin* or Dosulepin* or Dothiepin or Doxepin* or Duloxetine or DVS 233 or Enilospirone or Eptapirone or Escitalopram or Esketamine or Etoperidone or Femoxetine or Fenelzine or Fluotracen or Fluoxetine or Fluparoxan or Furazolidone or Fluvoxamine))) OR AB=((Agomelatine or Alnespirone or Amoxapine or Amersergide or Amfebutamone or Amiflamine or Amineptine or Amitriptylin* or Amitriptylinoxide or Amoxapine or Aripiprazole or Atomoxetine or Tomoxetine or Befloxatone or Benactyzine or Binospirone or Brofaromine or Bupropion or Butriptylin* or Cianopramine or Cilobamine or Cimoxatone or Citalopram or Chlorimipramin* or Clomipramin* or Chlomipramin* or Clorimipramine or Clorgyline or Clovoxamine or Dapoxetine or Deanol or Dibenzepin or Demexiptilin* or Deprenyl or Desipramine or Desvenlafaxine or Dibenzepin or Dimetacrin* or Dosulepin* or Dothiepin or Doxepin* or Duloxetine or DVS 233 or Enilospirone or Eptapirone or Escitalopram or Esketamine or Etoperidone or Femoxetine or Fenelzine or Fluotracen or Fluoxetine or Fluparoxan or Furazolidone or Fluvoxamine)) 9. (TI=((Harmaline or Harmine or Hyperforin or Hypericum or John* Wort or Idazoxan or Imipramin* or Iprindole or Iproniazid* or Ipsapirone or Imipraminoxide or Isocarboxazid* or Lesopitron or Levomilnacipran or Lithium or Lofepramin* or Lu AA21004 or Vortioxetine or Lu AA24530 or LY2216684 or Maprotiline or Medifoxamine or Melitracen or Metapramine or Methylphenidate or Mianserin or Milnacipran or Minaprine or Mirtazapine or Moclobemide or Nefazodone or Nialamide or Nitroxazepine or Nomifensine or Norfenfluramine or Nortriptyline or Noxiptilin*))) OR AB=((Harmaline or Harmine or Hyperforin or Hypericum or John* Wort or Idazoxan or Imipramin* or Iprindole or Iproniazid* or Ipsapirone or Imipraminoxide or Isocarboxazid* or Lesopitron or Levomilnacipran or Lithium or Lofepramin* or Lu AA21004 or Vortioxetine or Lu AA24530 or LY2216684 or Maprotiline or Medifoxamine or Melitracen or Metapramine or Methylphenidate or Mianserin or Milnacipran or Minaprine or Mirtazapine or Moclobemide or Nefazodone or Nialamide or Nitroxazepine or Nomifensine or Norfenfluramine or Nortriptyline or Noxiptilin*)) 10. (TI=((Opipramol or Oxaflozane or Paroxetine or Phenelzine or Pheniprazine or Pipofezin* or Pirandamine or Piribedil or Pirlindole or Pivagabine or Pizotyline or Propizepine or Protriptylin* or Pertofrane or Quinupramine or Quipazine or Reboxetine or Ritanserin or Rolipram or Scopolamine or Selegiline or Sertraline or Setiptiline or Teciptiline or Tandospirone or Tetrindole or Thiazesim or Thozalinone or Tianeptin* or Toloxatone or Tranylcypromine or Trazodone or Trimipramine or 5 Hydroxytryptophan or 5 HT or Tryptophan or Hydroxytryptophan or Venlafaxine or Viloxazine or Vilazodone or Viqualine or Vortioxetine or Zalospirone or Zimeldine))) OR AB=((Opipramol or Oxaflozane or Paroxetine or Phenelzine or Pheniprazine or Pipofezin* or Pirandamine or Piribedil or Pirlindole or Pivagabine or Pizotyline or Propizepine or Protriptylin* or Pertofrane or Quinupramine or Quipazine or Reboxetine or Ritanserin or Rolipram or Scopolamine or Selegiline or Sertraline or Setiptiline or Teciptiline or Tandospirone or Tetrindole or Thiazesim or Thozalinone or Tianeptin* or Toloxatone or Tranylcypromine or Trazodone or Trimipramine or 5 Hydroxytryptophan or 5 HT or Tryptophan or Hydroxytryptophan or Venlafaxine or Viloxazine or Vilazodone or Viqualine or Vortioxetine or Zalospirone or Zimeldine)) 11. TS= ((serotonin or norepinephrine or noradrenaline or nor epinephrine or nor adrenaline or neurotransmitt* or dopamine*) N3 (uptake or reuptake or "re-uptake")) 12. (TI=((psychotropic* or antidepress* or anti depress* o noradrenerg* or antiadrenergic or anti adrenergic or SSRI* or SNRI* or MAOI* or tricyclic* or "NARI" or "NARIs" or NDIR* or "SARI" or "SARIs" or NaSSA*))) OR AB=((psychotropic* or antidepress* or anti depress* o noradrenerg* or antiadrenergic or anti adrenergic or SSRI* or SNRI* or MAOI* or tricyclic* or "NARI" or "NARIs" or NDIR* or "SARI" or "SARIs" or NaSSA*)) 13. TS=((serotonin or monoamine oxidase or MAO) N2 inhibit*) 14. #8 OR #9 OR #10 OR #11 OR #12 OR #13 15. #6 AND #7 AND #14 and English or Spanish (Languages) 16. #6 AND #7 AND #14 and English or Spanish (Languages) 17. TS= (epidemilogic or longitudinal or retrospective or cross sectional or observational or cohort or case control) 18. #16 AND #17 |
| PsycINFO |
| 1. S32 not S33 2. S32 not S33 3. S30 OR S31 4. S30 OR S31 5. S13 AND S18 AND S28 6. S13 AND S18 AND S28 7. S13 AND S18 AND S28 8. S19 OR S20 OR S21 OR S22 OR S23 OR S24 OR S25 OR S26 OR S27 9. TI ( ((serotonin or monoamine oxidase or MAO) N2 inhibit*) ) OR AB ( ((serotonin or monoamine oxidase or MAO) N2 inhibit*) ) 10. TI ( (psychotropic* or antidepress* or anti depress* o noradrenerg* or antiadrenergic or anti adrenergic or SSRI* or SNRI* or MAOI* or tricyclic* or "NARI" or "NARIs" or NDIR* or "SARI" or "SARIs" or NaSSA*) ) OR AB ( (psychotropic* or antidepress* or anti depress* o noradrenerg* or antiadrenergic or anti adrenergic or SSRI* or SNRI* or MAOI* or tricyclic* or "NARI" or "NARIs" or NDIR* or "SARI" or "SARIs" or NaSSA*) ) 11. TI ( ((serotonin or norepinephrine or noradrenaline or nor epinephrine or nor adrenaline or neurotransmitt* or dopamine*) N3 (uptake or reuptake or "re-uptake")) ) OR AB ( ((serotonin or norepinephrine or noradrenaline or nor epinephrine or nor adrenaline or neurotransmitt* or dopamine*) N3 (uptake or reuptake or "re-uptake")) ) 12. TI ( (Opipramol or Oxaflozane or Paroxetine or Phenelzine or Pheniprazine or Pipofezin* or Pirandamine or Piribedil or Pirlindole or Pivagabine or Pizotyline or Propizepine or Protriptylin* or Pertofrane or Quinupramine or Quipazine or Reboxetine or Ritanserin or Rolipram or Scopolamine or Selegiline or Sertraline or Setiptiline or Teciptiline or Tandospirone or Tetrindole or Thiazesim or Thozalinone or Tianeptin* or Toloxatone or Tranylcypromine or Trazodone or Trimipramine or 5 Hydroxytryptophan or 5 HT or Tryptophan or Hydroxytryptophan or Venlafaxine or Viloxazine or Vilazodone or Viqualine or Vortioxetine or Zalospirone or Zimeldine) ) OR 13. TI ( (Harmaline or Harmine or Hyperforin or Hypericum or John* Wort or Idazoxan or Imipramin* or Iprindole or Iproniazid* or Ipsapirone or Imipraminoxide or Isocarboxazid* or Lesopitron or Levomilnacipran or Lithium or Lofepramin* or Lu AA21004 or Vortioxetine or Lu AA24530 or LY2216684 or Maprotiline or Medifoxamine or Melitracen or Metapramine or Methylphenidate or Mianserin or Milnacipran or Minaprine or Mirtazapine or Moclobemide or Nefazodone or Nialamide or Nitroxazepine or Nomifensine or Norfenfluramine or Nortriptyline or Noxiptilin*) ) OR AB ( (Harmaline or Harmine or Hyperforin or Hypericum or John* Wort or Idazoxan or 14. TI ( (Agomelatine or Alnespirone or Amoxapine or Amersergide or Amfebutamone or Amiflamine or Amineptine or Amitriptylin* or Amitriptylinoxide or Amoxapine or Aripiprazole or Atomoxetine or Tomoxetine or Befloxatone or Benactyzine or Binospirone or Brofaromine or Bupropion or Butriptylin* or Cianopramine or Cilobamine or Cimoxatone or Citalopram or Chlorimipramin* or Clomipramin* or Chlomipramin* or Clorimipramine or Clorgyline or Clovoxamine or Dapoxetine or Deanol or Dibenzepin or Demexiptilin* or Deprenyl or Desipramine or Desvenlafaxine or Dibenzepin or Dimetacrin* or Dosulepin* or Dothiepin or Doxepin* or Duloxetine or "DVS 233" or Enilospirone or Eptapirone or Escitalopram or Esketamine or Etoperidone or Femoxetine or Fenelzine or Fluotracen or Fluoxetine or Fluparoxan or Furazolidone or Fluvoxamine) ) OR AB ( (Agomelatine or Alnespirone or Amoxapine or Amersergide or Amfebutamone or Amiflamine or Amineptine or Amitriptylin* or Amitriptylinoxide or Amoxapine or Aripiprazole or Atomoxetine or Tomoxetine or Befloxatone or Benactyzine or Binospirone or Brofaromine or Bupropion or Butriptylin* or Cianopramine or Cilobamine or Cimoxatone or Citalopram or Chlorimipramin* or Clomipramin* or Chlomipramin* or Clorimipramine or Clorgyline or Clovoxamine or Dapoxetine or Deanol or Dibenzepin or Demexiptilin* or Deprenyl or Desipramine or Desvenlafaxine or Dibenzepin or Dimetacrin* or Dosulepin* or Dothiepin or Doxepin* 15. DE "Monoamine Oxidase Inhibitors" OR DE "Iproniazid" OR DE "Isocarboxazid" OR DE "Moclobemide" OR DE "Nialamide" OR DE "Pargyline" OR DE "Phenelzine" OR DE "Tranylcypromine" 16. DE "Neurotransmitter Uptake Inhibitors" OR DE "Atomoxetine" OR DE "Serotonin Norepinephrine Reuptake Inhibitors" OR DE "Serotonin Reuptake Inhibitors" 17. MM "Antidepressant Drugs" 18. S14 OR S15 OR S16 OR S17 19. DE (adhere* or "non adherence" or "nonadherence" or "non-adherence" or complian* or "non complian*" or "non-complian*" or concordance or persistence or acceptance) 20. MA treatment refusal 21. MA medication adherence 22. MA patient compliance 23. S6 NOT S12 24. S7 OR S8 OR S9 OR S10 OR S11 25. TI ( (Schizoaffective or schizo-affective) ) OR AB ( (Schizoaffective or schizo-affective) ) 26. TI ( (mania or manic*) ) OR AB ( (mania or manic*) ) 27. TI ( (bipolar or bipolar disorder*) ) OR AB ( (bipolar or bipolar disorder*) ) 28. MA psychotic disorders 29. MA bipolar disorder 30. S1 OR S2 OR S3 OR S4 OR S5 31. TI ( (depress* or mood? or affective disorder* or affective symptom* or depress* disorder* or dysphoria or endogenous depression or involutional depression or major depression or masked depression or melancholia or mood disorder or organic depression or recurrent brief depression or anaclitic depression or recurrent depression or treatment resistant depression or dysthymic disorder or reactive depression or agitated depression or atypical depression or sadness) ) OR AB ( (depress* or mood? or affective disorder* or affective symptom* or depress* disorder* or dysphoria or endogenous depression or involutional depression or major depression or masked depression or melancholia or mood disorder or organic depression or recurrent brief depression or anaclitic depression or recurrent depression or treatment resistant depression or dysthymic disorder or reactive depression or agitated depression or atypical depression or sadness) ) 32. MA mood disorders 33. MA depressive disorder, major 34. MA depressive disorder 35. MA depression |

Supplementary material 2. Excluded studies and reason for exclusion

A. SR update

| Abstract (n = 7) |
| --- |
| 1. Etchepare, F., Sanglier, T., André, M., Verdoux, H., Tournier, M. (2013). Comparison of the antidepressant treatment patterns in younger and older adults using the french health insurance claims database. *Pharmacoepidemiology and drug safety*,*22(S1)*, 496–497. <https://doi.org/10.1002/pds.3512> 2. Gerlach, L.B., Kavanagh, J., Chiang, C., Kim, H., Kales, H. C. (2013).With a little help from my friends?: The role of social support in adherence to antidepressant medication. *American Journal of Geriatric Psychiatr,21*(S3), S70-S71. https://doi.org/10.1016/j.jagp.2012.12.091 3. Lauzier, S., Kadachi, H., Moisan, J., Fleury, M., Lesage, A., Vanasse, A., Courteau, J., & Grégoire, J. P. (2014). Neighborhood material and social deprivation and adherence to antidepressant treatment in depression. *Pharmacoepidemiology and Drug Safety,23*(S1), *35-36.* https://doi.org/ 10.1002/pds.3701 4. Lenderking, W. R., Samp, J., Hanlon, J., Hsieh, R. Akhras, K. S., Revicki, D. A. (2013). Factors associated with poor adherence in patients initiating medication for major depressive disorder: Interim results from a prospective, longitudinal study. *Value Health, 16*(7), A550. https://doi.org/10.1016/j.jval.2013.08.1417 5. Noh, Y., Choe, S.A., Shin, J.Y. (2020). Discontinuation and reinitiation of antidepressants during pregnancy in South Korea, 2013-2017. *Pharmacoepidemiology and Drug Safety, 29*(S3), 540-541. <https://doi.org/10.1002/pds.5114> 6. Voshaar,R.O., Wouters, H., Holvast, F., Hek, K., Schellevis, F., Burger, H., & Verhaak,P. (2018). Non-adherence to antidepressants among older patients with depression: A longitudinal cohort study in primary care. *European Geriatric Medicine,9*(S1), 1-367. https://doi.org/10.1007/s41999-018-0097 7. Wu, J., Sykes, L., Keiser, S., & Davis-Ajami, M. L. (2012). PMH34 Persistence in Use of Antidepressants in Pregnant Women Enrolled in Medicaid in the United States. *Value in Health,15*(7), A340. https://doi.org/10.1016/j.jval.2012.08.824 |
| Study Design (n = 7) |
| 1. Baeza-Velasco, C., Olié, E., Béziat, S., Guillaume, S., & Courtet, P. (2019). Determinants of suboptimal medication adherence in patients with a major depressive episode. *Depression and anxiety*, *36*(3), 244–251. https://doi.org/10.1002/da.22852 |
| 1. De Las Cuevas, C., Motuca, M., Baptista, T., Villasante-Tezanos, G. A., Lazary, J., Pogany, L., & De Leon, J. (2021). Poor Adherence to Oral Psychiatric Medication in Adults with Depression: Psychological Reactance May Have Specific Effects in Depression. *Neuropsychopharmacologia Hungarica : a Magyar Pszichofarmakologiai Egyesulet lapja = official journal of the Hungarian Association of Psychopharmacology*, *23*(4), 374–387. |
| 1. Eshtehardi, S. S., Taylor, A. A., Chen, T. A., de Dios, M. A., Correa-Fernández, V., Kendzor, D. E., Businelle, M. S., & Reitzel, L. R. (2021). Sociodemographic Determinants of Nonadherence to Depression and Anxiety Medication among Individuals Experiencing Homelessness. *International journal of environmental research and public health*, *18*(15), 7958. <https://doi.org/10.3390/ijerph18157958> 2. Gallagher, S. P., Insel, K., Badger, T. A., & Reed, P. (2018). Antidepressant adherence in United States active duty Army Soldiers: A small descriptive study. *Archives of psychiatric nursing*, *32*(6), 793–801. <https://doi.org/10.1016/j.apnu.2018.06.002> 3. Jang, S., Cho, H., Kang, C., & Jang, S. (2020). Antidepressant adherence and its predictors in immigrants with depression: A population-based study. *Medicine*, *99*(51), e23308. <https://doi.org/10.1097/MD.0000000000023308> 4. López-Torres, J., Párraga, I., Del Campo, J. M., Villena, A., & ADSCAMFYC Group (2013). Follow up of patients who start treatment with antidepressants: treatment satisfaction, treatment compliance, efficacy and safety. *BMC psychiatry*, *13*, 65. <https://doi.org/10.1186/1471-244X-13-65> 5. Nwokeji, E. D., Bohman, T. M., Wallisch, L., Stoner, D., Christensen, K., Spence, R. R., Reed, B. C., & Ostermeyer, B. (2012). Evaluating patient adherence to antidepressant therapy among uninsured working adults diagnosed with major depression: results of the Texas Demonstration to Maintain Independence and Employment study. *Administration and policy in mental health*, *39*(5), 374–382. https://doi.org/10.1007/s10488-011-0354-z |
| Exposure Factor (n = 9) |
| 1. Bao, Y., Ryan, A. M., Shao, H., Pincus, H. A., & Donohue, J. M. (2013). Generic initiation and antidepressant therapy adherence under Medicare Part D. The American journal of managed care, 19(12), 989–998. 2. Bosman, J., Ter Horst, P. G., Smit, J. P., Dijkstra, J. R., Beekhuis, H. R., Slingersland, R. J., & Hospes, W. (2014). Adherence of antidepressants during pregnancy: MEMS compared with three other methods. *Therapeutic advances in psychopharmacology*, *4*(2), 61–69. <https://doi.org/10.1177/2045125313511486> 3. Chakraborty, S. (2021). Antidepressant compliance in depression during the COVID pandemic: Identifying the potential poor compliers in an industrial hospital. *Ann Indian Psychiatry, 5*(2), 120-125. https://doi.org/ 0.4103/aip.aip_30_21 4. Gauthier, G., Guérin, A., Zhdanava, M., Jacobson, W., Nomikos, G., Merikle, E., François, C., & Perez, V. (2017). Treatment patterns, healthcare resource utilization, and costs following first-line antidepressant treatment in major depressive disorder: a retrospective US claims database analysis. *BMC psychiatry*, *17*(1), 222. <https://doi.org/10.1186/s12888-017-1385-0> 5. Keyloun, K. R., Hansen, R. N., Hepp, Z., Gillard, P., Thase, M. E., & Devine, E. B. (2017). Adherence and Persistence Across Antidepressant Therapeutic Classes: A Retrospective Claims Analysis Among Insured US Patients with Major Depressive Disorder (MDD). *CNS drugs*, *31*(5), 421–432. <https://doi.org/10.1007/s40263-017-0417-0> 6. Novick, D., Montgomery, W., Moneta, V., Peng, X., Brugnoli, R., & Haro, J. M. (2015). Antidepressant medication treatment patterns in Asian patients with major depressive disorder. *Patient preference and adherence*, *9*, 421–428. <https://doi.org/10.2147/PPA.S68432> 7. Rajper,A. B., Dars,J. A., Iqbal,F., Rasool,G., Lal,C., Hyder,M., Afridi,M. I., Minhas,F. A., Abbas,K. (2022). Factors leading to Non-Compliance to Antidepressants Among Patients with Major Depressive Disorder: A Cohort Study from Pakistan. *Pakistan Journal of Medical & Health Sciences, 16*(11), 441-443. https://doi.org/10.53350/pjmhs22161441 8. Sicras-Mainar, A., Mauriño, J., Cordero, L., Blanca-Tamayo, M., & Navarro-Artieda, R. (2012). Costes y factores asociados a las respuestas óptima y subóptima al tratamiento del trastorno depresivo mayor en atención primaria [Costs and associated factors with optimal and suboptimal responses to the treatment of major depressive disorder]. *Atencion primaria*, *44*(11), 667–675. <https://doi.org/10.1016/j.aprim.2012.04.007> 9. Wagner, C. J., Dintsios, C. M., Metzger, F. G., L'Hoest, H., Marschall, U., Stollenwerk, B., & Stock, S. (2018). Longterm persistence and nonrecurrence of depression treatment in Germany: a four-year retrospective follow-up using linked claims data. *International journal of methods in psychiatric research*, *27*(2), e1607. <https://doi.org/10.1002/mpr.1607> |
| Population (n = 22) |
| 1. Assayag, J., Forget, A., Kettani, F. Z., Beauchesne, M. F., Moisan, J., & Blais, L. (2013). The impact of the type of insurance plan on adherence and persistence with antidepressants: a matched cohort study. *Canadian journal of psychiatry. Revue canadienne de psychiatrie*, *58*(4), 233–239. <https://doi.org/10.1177/070674371305800409> 2. Bérard, A., Sheehy, O., Zhao, J. P., Chambers, C., Roth, M., Bozzo, P., Johnson, D., Kao, K., Lavigne, S., Wolfe, L., Quinn, D., Dieter, K., & MotherToBaby Collaborative Research Committee (2019). Impact of antidepressant use, discontinuation, and dosage modification on maternal depression during pregnancy. *European neuropsychopharmacology : the journal of the European College of Neuropsychopharmacology*, *29*(7), 803–812. <https://doi.org/10.1016/j.euroneuro.2019.06.007> 3. Bet, P. M., Penninx, B. W., van Laer, S. D., Hoogendijk, W. J., & Hugtenburg, J. G. (2015). Current and remitted depression and anxiety disorders as risk factors for medication nonadherence. *The Journal of clinical psychiatry*, *76*(9), e1114–e1121. <https://doi.org/10.4088/JCP.14m09001> 4. Burton, C., Anderson, N., Wilde, K., & Simpson, C. R. (2012). Factors associated with duration of new antidepressant treatment: analysis of a large primary care database. *The British journal of general practice : the journal of the Royal College of General Practitioners*, *62*(595), e104–e112. <https://doi.org/10.3399/bjgp12X625166> 5. De las Cuevas, C., Peñate, W., & Sanz, E. J. (2014). Risk factors for non-adherence to antidepressant treatment in patients with mood disorders. *European journal of clinical pharmacology*, *70*(1), 89–98. <https://doi.org/10.1007/s00228-013-1582-9> 6. Degli Esposti, L., Piccinni, C., Sangiorgi, D., Fagiolini, A., & Buda, S. (2015). Patterns of antidepressant use in Italy: therapy duration, adherence and switching. *Clinical drug investigation*, *35*(11), 735–742. <https://doi.org/10.1007/s40261-015-0332-4> 7. Etchepare, F., Sanglier, T., André, M., Verdoux, H., & Tournier, M. (2014). Antidepressant treatment patterns in younger and older adults from the general population in a real-life setting. *International journal of geriatric psychiatry*, *29*(9), 928–935. <https://doi.org/10.1002/gps.4081> 8. Falcaro, M., Ben-Shlomo, Y., King, M., Freemantle, N., & Walters, K. (2019). Factors associated with discontinuation of antidepressant treatment after a single prescription among patients aged 55 or over: evidence from English primary care. Social psychiatry and psychiatric epidemiology, 54(12), 1545–1553. https://doi.org/10.1007/s00127-019-01678-x 9. Freccero, C., Sundquist, K., Sundquist, J., & Ji, J. (2016). Primary adherence to antidepressant prescriptions in primary health care: a population-based study in Sweden. *Scandinavian journal of primary health care*, *34*(1), 83–88. <https://doi.org/10.3109/02813432.2015.1132884> 10. Germack, H. D., Combellick, J., Cooper, M., Koller, K., & McMichael, B. (2022). Antidepressants Are the Most Commonly Discontinued Psychotherapeutic Medications in Pregnancy. *Women's health issues : official publication of the Jacobs Institute of Women's Health*, *32*(3), 241–250. <https://doi.org/10.1016/j.whi.2021.10.004> 11. Hafferty, J. D., Wigmore, E. M., Howard, D. M., Adams, M. J., Clarke, T. K., Campbell, A. I., MacIntyre, D. J., Nicodemus, K. K., Lawrie, S. M., Porteous, D. J., & McIntosh, A. M. (2019). Pharmaco-epidemiology of antidepressant exposure in a UK cohort record-linkage study. *Journal of psychopharmacology (Oxford, England)*, *33*(4), 482–493. <https://doi.org/10.1177/0269881119827888> 12. Kap, E., Konrad, M., & Kostev, K. (2019). Persistence with selective serotonin (norepinephrine) reuptake inhibitors in Germany-A retrospective database analysis. *Journal of affective disorders*, *247*, 156–160. <https://doi.org/10.1016/j.jad.2019.01.016> 13. Krivoy, A., Balicer, R. D., Feldman, B., Hoshen, M., Zalsman, G., Weizman, A., & Shoval, G. (2015). The impact of age and gender on adherence to antidepressants: a 4-year population-based cohort study. *Psychopharmacology*, *232*(18), 3385–3390. <https://doi.org/10.1007/s00213-015-3988-9> 14. Párraga Martínez, I., López-Torres Hidalgo, J., del Campo del Campo, J. M., Villena Ferrer, A., Morena Rayo, S., Escobar Rabadán, F., & en representación del Grupo ADSCAMFYC (2014). Seguimiento de la adherencia al tratamiento antidepresivo en pacientes que inician su consumo [Adherence to patients antidepressant treatment and the factors associated of non-compiance]. *Atencion primaria*, *46*(7), 357–366. <https://doi.org/10.1016/j.aprim.2013.11.003> 15. Poluzzi, E., Piccinni, C., Sangiorgi, E., Clo, M., Tarricone, I., Menchetti, M., & De Ponti, F. (2013). Trend in SSRI-SNRI antidepressants prescription over a 6-year period and predictors of poor adherence. *European journal of clinical pharmacology*, *69*(12), 2095–2101. <https://doi.org/10.1007/s00228-013-1567-8> 16. Roca, A., Imaz, M. L., Torres, A., Plaza, A., Subirà, S., Valdés, M., Martin-Santos, R., & Garcia-Esteve, L. (2013). Unplanned pregnancy and discontinuation of SSRIs in pregnant women with previously treated affective disorder. *Journal of affective disorders*, *150*(3), 807–813. <https://doi.org/10.1016/j.jad.2013.02.040> 17. Serna, M. C., Real, J., Cruz, I., Galván, L., & Martin, E. (2015). Monitoring patients on chronic treatment with antidepressants between 2003 and 2011: analysis of factors associated with compliance. *BMC public health*, *15*, 1184. <https://doi.org/10.1186/s12889-015-2493-8> 18. Singer, A. G., LaBine, L., Katz, A., Yogendran, M., & Lix, L. (2022). Primary medication nonadherence in a large primary care population: Observational study from Manitoba. *Canadian family physician Medecin de famille canadien*, *68*(7), 520–527. <https://doi.org/10.46747/cfp.6807520> 19. Slabbert, F. N., Harvey, B. H., Brink, C. B., & Lubbe, M. S. (2015). The impact of HIV/AIDS on compliance with antidepressant treatment in major depressive disorder: A prospective study in a South African private healthcare cohort. *AIDS research and therapy*, *12*, 9. <https://doi.org/10.1186/s12981-015-0050-2> 20. Sundell, K. A., Waern, M., Petzold, M., & Gissler, M. (2013). Socio-economic determinants of early discontinuation of anti-depressant treatment in young adults. *European journal of public health*, *23*(3), 433–440. <https://doi.org/10.1093/eurpub/ckr137> 21. Trifirò, G., Tillati, S., Spina, E., Ferrajolo, C., Alacqua, M., Aguglia, E., Rizzi, L., Caputi, A. P., Cricelli, C., & Samani, F. (2013). A nationwide prospective study on prescribing pattern of antidepressant drugs in Italian primary care. *European journal of clinical pharmacology*, *69*(2), 227–236. https://doi.org/10.1007/s00228-012-1319-1 22. Wikman, A., Skalkidou, A., Wikström, A. K., Lampa, E., Kramer, M. S., Yong, E. L., Skoglund, C., Epperson, N., & Sundström-Poromaa, I. (2020). Factors associated with re-initiation of antidepressant treatment following discontinuation during pregnancy: a register-based cohort study. *Archives of women's mental health*, *23*(5), 709–717. https://doi.org/10.1007/s00737-020-01050-y |

B. Previous SR

| Study Design (n = 17) |
| --- |
| 1. Aikens, J. E., Nease, D. E., Jr, Nau, D. P., Klinkman, M. S., & Schwenk, T. L. (2005). Adherence to maintenance-phase antidepressant medication as a function of patient beliefs about medication. *Annals of family medicine*, *3*(1), 23–30. <https://doi.org/10.1370/afm.238> 2. Ayalon, L., Areán, P. A., & Alvidrez, J. (2005). Adherence to antidepressant medications in black and Latino elderly patients. *The American journal of geriatric psychiatry : official journal of the American Association for Geriatric Psychiatry*, *13*(7), 572–580. <https://doi.org/10.1176/appi.ajgp.13.7.572> 3. Bull, S. A., Hu, X. H., Hunkeler, E. M., Lee, J. Y., Ming, E. E., Markson, L. E., & Fireman, B. (2002). Discontinuation of use and switching of antidepressants: influence of patient-physician communication. *JAMA, 288*(11), 1403–1409. <https://doi.org/10.1001/jama.288.11.1403> 4. Burra, T. A., Chen, E., McIntyre, R. S., Grace, S. L., Blackmore, E. R., & Stewart, D. E. (2007). Predictors of self-reported antidepressant adherence. *Behavioral medicine (Washington, D.C.), 32*(4), 127–134. <https://doi.org/10.3200/BMED.32.4.127-134> 5. Crown, W. H., Treglia, M., Meneades, L., & White, A. (2001). Long-term costs of treatment for depression: impact of drug selection and guideline adherence. *Value in health: the journal of the International Society for Pharmacoeconomics and Outcomes Research, 4*(4), 295–307. <https://doi.org/10.1046/j.1524-4733.2001.44084.x> 6. Demyttenaere K. (2001). Compliance and acceptance in antidepressant treatment. *International journal of psychiatry in clinical practice*, *5*(1), 29–35. <https://doi.org/10.1080/13651500152048423> 7. Granger, A. L., Fehnel, S. E., Hogue, S. L., Bennett, L., & Edin, H. M. (2006). An assessment of patient preference and adherence to treatment with Wellbutrin SR: a web-based survey. *Journal of affective disorders*, *90*(2-3), 217–221. <https://doi.org/10.1016/j.jad.2005.08.018> 8. Madsen, J. W., McQuaid, J. R., & Craighead, W. E. (2009). Working with reactant patients: are we prescribing nonadherence?. *Depression and anxiety*, *26*(2), 129–134. <https://doi.org/10.1002/da.20523> 9. Maidment, R., Livingston, G., & Katona, C. (2002). Just keep taking the tablets: adherence to antidepressant treatment in older people in primary care. *International journal of geriatric psychiatry*, *17*(8), 752–757. <https://doi.org/10.1002/gps.688> 10. Oller-Canet, S., Fernández-San Martín, M. I., García-Lecina, R., Castro Rodríguez, J. I., Font-Canal, T., Lacasta-Tintorer, D., Martín-López, L. M., & Flamarich-Zampalo, D. (2011). ¿Toman los pacientes deprimidosel tratamiento prescrito? Estudiodescriptivo sobre el cumplimientodel tratamiento antidepresivo [Do depressed patients comply with treatments prescribed?: a cross-sectional study of adherence to the antidepressant treatment]. *Actas espanolas de psiquiatria*, *39*(5), 288–293. 11. Pfeiffer, P. N., Ganoczy, D., Zivin, K., & Valenstein, M. (2011). Benzodiazepines and adequacy of initial antidepressant treatment for depression. *Journal of clinical psychopharmacology*, *31*(3), 360–364. <https://doi.org/10.1097/JCP.0b013e318217b4c4> 12. Roca, M., Armengol, S., Salvador-Carulla, L., Monzón, S., Salvà, J., & Gili, M. (2011). Adherence to medication in depressive patients. *Journal of clinical psychopharmacology, 31*(4), 541–543. <https://doi.org/10.1097/JCP.0b013e3182223af9> 13. Russell, J., & Kazantzis, N. (2008). Medication beliefs and adherence to antidepressants in primary care. *The New Zealand medical journal*, *121*(1286), 14–20. 14. Sanglier, T., Saragoussi, D., Milea, D., Auray, J. P., Valuck, R. J., & Tournier, M. (2011). Comparing antidepressant treatment patterns in older and younger adults: a claims database analysis. *Journal of the American Geriatrics Society*, *59*(7), 1197–1205. https://doi.org/10.1111/j.1532-5415.2011.03457.x 15. Sher, I., McGinn, L., Sirey, J. A., & Meyers, B. (2005). Effects of caregivers' perceived stigma and causal beliefs on patients' adherence to antidepressant treatment. *Psychiatric services (Washington, D.C.)*, *56*(5), 564–569. <https://doi.org/10.1176/appi.ps.56.5.564> 16. Voils, C. I., Steffens, D. C., Flint, E. P., & Bosworth, H. B. (2005). Social support and locus of control as predictors of adherence to antidepressant medication in an elderly population. *The American journal of geriatric psychiatry : official journal of the American Association for Geriatric Psychiatry*, *13*(2), 157–165. <https://doi.org/10.1176/appi.ajgp.13.2.157> 17. Yeh, M. Y., Sung, S. C., Yorker, B. C., Sun, C. C., & Kuo, Y. L. (2008). Predictors of adherence to an antidepressant medication regimen among patients diagnosed with depression in Taiwan. *Issues in mental health nursing*, *29*(7), 701–717. <https://doi.org/10.1080/01612840802129038> |
| Exposure Factor (n = 1) |
| 1. White, T. J., Vanderplas, A., Ory, C., Dezii, C. M., & Chang, E. (2003). Economic impact of patient adherence with antidepressant therapy within a managed care organization. *Disease Management & Health Outcomes*, *11*, 817-822. https://doi.org/10.2165/00115677-200311120-00006 |

Supplementary Table 3. Main findings obtained in the included studies. Sociodemographic Factors – Implementation

| Predictor factor | Author, year | Follow-up (weeks) | N adherence patients | Univariate and other statistics | Multivariate statistics | Group | Cohort | Model adjustment | Reference |
| --- | --- | --- | --- | --- | --- | --- | --- | --- | --- |
| Implementation | | | | | | | | | |
| Age | Akincigil, 2007 | 16 | 2186 | NR | (OR: 1.22; 95%CI: 0.98-1.53) | 25-39 y | NA | NR | 18-25 y |
|  |  | 16 | 2186 | NR | (OR: 1.71; 95%CI: 1.36-2.15) | 40-49 y | NA | NR | 18-25 y |
|  |  | 16 | 2186 | NR | (OR: 2.48; 95%CI: 1.94-3.15) | 50-66 y | NA | NR | 18-25 y |
|  |  | 16 | 2186 | NR | (OR: 1.96; 95%CI: 1.34-2.85) | ≥65 y | NA | NR | 18-25 y |
|  |  | 33 | 922 | NR | (OR: 0.82; 95%CI: 0.57-1.18) | 25-39 y | NA | NR | 18-25 y |
|  |  | 33 | 922 | NR | (OR: 1.22; 95%CI: 0.85-1.75) | 40-49 y | NA | NR | 18-25 y |
|  |  | 33 | 922 | NR | (OR: 1.41; 95%CI: 0.96-2.02) | 50-66 y | NA | NR | 18-25 y |
|  |  | 33 | 922 | NR | (OR: 1.20; 95%CI: 0.71-2.04) | ≥ 65 y | NA | NR | 18-25 y |
|  | Bhattacharjee, 2020 | 16 | 4644 | (X^2^: 2.61; p= 0.11) | (OR: 1.14; 99%CI: 0.93-1.40; p= 0.11) | ≥ 75 y | NA | NR | 65-74 y |
|  |  | 50 | 3584 | (X^2^: 3.85; p= 0.05) | (OR: 1.16; 99%CI: 0.95-1.42; p= 0.05) | ≥ 75 y | NA | NR | 65-74 y |
|  | Chen, 2010 | 12 | 4102 | (OR: 1.38; 95%CI: 1.19-1.60) | NA | 35-49 y | Acute | NR | 18-34 y |
|  |  | 12 | 4102 | (OR: 1.39; 95%CI: 1.15-1.68) | NA | 56-64 y | Acute | NR | 18-34 y |
|  |  | 12 | 4102 | (OR: 2.77; 95%CI: 1.67-4.58) | NA | ≥65 y | Acute | NR | 18-34 y |
|  |  | 39 | 1921 | (OR: 1.40; 95%CI: 1.12-1.74) | NA | 35-49 y | Continuation | NR | 18-34 y |
|  |  | 39 | 1921 | (OR: 1.81; 95%CI: 1.36-2.39) | NA | 56-64 y | Continuation | NR | 18-34 y |
|  |  | 39 | 1921 | (OR: 1.50; 95%CI: 0.81-2.80) | NA | ≥65 y | Continuation | NR | 18-34 y |
|  | Cohen, 2004 | 14 | NR | (r: 0.18; p= 0.18) | NA | Age | NA | NA | Continuous |
|  | Gerlach, 2019 | 52 | 190 | NR | (OR: 1.35; 95% CI: 0.78-2.31; p= 0.28) | NA | NA | NR | 60-64 y |
|  |  | 52 | 190 | NR | (OR: 0.93; 95% CI: 0.42-2.08; p= 0.87) | NA | NA | NR | 60-64 y |
|  | Holvast, 2019 | 52 | 878 | (OR: 0.99; 95% CI: 0.96-1.01; p= NS) | NA | NA | NA | NA | Continuous |
|  | Keeley, 2007 | 12 | 12 | (p= 0.70) | NA | Age | NA | NA | NA |
|  | Kogut, 2016 | 4 | 119 | (p= 0.22) | NA | NA | NA | NA | NR |
|  |  | 12 | 1983 | NR | (OR: 1.57; 95%CI: 1.29-1.91; p <0.001) | NA | NA | Plan and regimen type, diabetes mellitus, office and psychiatric visits | 18-34 y |
|  |  | 12 | 87 | (p= 0.28) | NA | NA | NA | NA | NR |
|  | Lin, 2011 | 52 | 2111615 | (Beta: 7.38; SE: 7.38) | NA | 26-49 y | NA | NA | 18-25 y |
|  |  | 52 | 2111615 | (Beta: 14.22; SE: 8.29) | NA | 56-64 y | NA | NA | 18-25 y |
|  |  | 52 | 2111615 | (Beta: 15.09; SE: 10.52) | NA | ≥ 65 y | NA | NA | 18-25 y |
|  | Liu, 2010 | 52 | NR | (OR: 1.23; 95%CI: 0.93-1.61) | NA | 26-35 y | NA | NA | 18-25 y |
|  |  | 52 | NR | (OR: 1.63; 95%CI: 1.28-2.08) | NA | 36-45 y | NA | NA | 18-25 y |
|  |  | 52 | NR | (OR: 1.88; 95%CI: 1.48-2.39) | NA | 46-55 y | NA | NA | 18-25 y |
|  |  | 52 | NR | (OR: 2.20; 95%CI: 1.71-2.81) | NA | 56-64 y | NA | NA | 18-25 y |
|  | Liu, 2011 | 52 | 12689 | NR | (OR: 1.47; 95%CI: 1.38-1.57; p <0.05) | 36-45 y | NA | Antidepressant, gender, age, health plan type, residence, comorbid diseases, and prior medications | 18-35 y |
|  |  | 52 | 12689 | NR | (OR: 1.65; 95%CI: 1.55-1.75; p <0.05) | 46-55 y | NA |  | 18-35 y |
|  |  | 52 | 12689 | NR | (OR: 2.06; 95%CI: 1.93-2.21; p <0.05) | 56-64 y | NA |  | 18-35 y |
|  | Shin, 2020 | 12 | NR | (RR: 1.02; 95%CI: 1.01-1.03; p <0.01) | (RR: 0.92; 95%CI: 0.91-0.93; p <0.01) | 35-49 y | NA | Policy, gender, age, and type of index medication | 19-34 y |
|  |  | 12 | NR | (RR: 1.08; 95%CI: 1.06-1.09; p <0.01) | (RR: 0.84; 95%CI: 0.83-0.85; p <0.01) | 56-64 y | NA |  | 19-34 y |
|  |  | 12 | NR | (RR: 1.12; 95%CI: 1.11-1.14; p <0.01) | (RR: 0.77; 95%CI: 0.75-0.77; p <0.01) | ≥65 y | NA |  | 19-34 y |
|  |  | 26 | NR | (RR: 0.90; 95%CI: 0.87-0.93; p <0.01) | (RR: 1.09; 95%CI: 1.04-1.13; p <0.01) | 35-49 y | NA |  | 19-34 y |
|  |  | 26 | NR | (RR: 0.81; 95%CI: 0.78-0.85; p <0.01) | (RR: 1.15; 95%CI: 1.10-1.21; p <0.01) | 56-64 y | NA |  | 19-34 y |
|  |  | 26 | NR | (RR: 1.00; 95%CI: 0.98-1.05) | (RR: 0.86; 95%CI: 0.83-0.89; p <0.01) | ≥65 y | NA |  | 19-34 y |
|  | McLaughlin, 2007 | 39 | 747 | (OR: 1.01; Wald CI: 1.008-1.012; p <0.05) | NA | NA | NA | NA | Continuous |
|  | Merrick, 2012 | 16 | 383 | NR | (OR: 2.40; 95%CI: 1.20-4.80; p <0.05) | 60-74 y | NA | Conc-Top 2 model: sex, age, white, primary diagnosis of major depression in episode, CCI, behavioural health comorbidity, initial regimen includes SSRI, psychiatric prescriber, and state  Conc-Top 2 model: sex, age, white, primary diagnosis of major depression in episode, CCI, behavioural health comorbidity, initial regimen includes SSRI, psychiatric prescriber, and state | ≤45 y |
|  |  | 16 | 383 | NR | (OR: 2.70; 95%CI: 1.40-5.40; p <0.01) | ≥75 y | NA |  | ≤45 y |
|  |  | 16 | 383 | NR | (OR: 1.80; 95%CI: 0.80-3.70) | 45-59 y | NA |  | ≤45 y |
|  |  | 16 | 383 | NR | (OR: 2.40; 95%CI: 1.20-4.80; p <0.05) | 60-74 y | NA |  | ≤45 y |
|  |  | 16 | 383 | NR | (OR: 1.70; 95%CI: 0.80-3.70) | 60-74 y | NA | Conc-Herfindahl model: sex, age, white, primary diagnosis of major depression in episode, CCI, behavioural health comorbidity, initial regimen includes SSRI, psychiatric prescriber, and state | ≤45 y |
|  |  | 16 | 383 | NR | (OR: 2.40; 95%CI: 1.20-4.70; p <0.05) | ≥75 y | NA |  | ≤45 y |
|  |  | 16 | 383 | NR | (OR: 2.70; 95%CI: 1.40-5.40; p <0.01) | 45-59 y | NA |  | ≤45 y |
|  | Sirey, 2001 | 12 | 96 | (OR: 2.91; 95%CI: 1.03-8.24; p= 0.04) | NA | ≥60 y | NA | NR | <60 y |
|  | Stang, 2007 | 39 | 826 | (OR: 1.03; 95%CI: 1.02-1.03; p <0.05) | NA | NA | Both | NR | Continuous |
|  | Ten Doesschate, 2009 | 104 | 91 | (OR: 1.00; 95%CI: 0.96-1.05; p= 0.85) | NA | NA | NA | NA | Continuous |
|  | Wu, 2012 | 52 | NR | (OR: 1.39; 95%CI: 1.15-1.67) | NA | 31-40 y | NA | NA | 18-30 y |
|  |  | 52 | NR | (OR: 1.73; 95%CI: 1.40-2.14) | NA | 41-50 y | NA | NA | 18-30 y |
|  |  | 52 | NR | (OR: 1.90; 95%CI: 1.45-2.49) | NA | 51-60 y | NA | NA | 18-30 y |
|  |  | 52 | NR | (OR: 1.91; 95%CI: 1.05-3.46) | NA | 60-64 y | NA | NA | 18-30 y |
|  |  | 52 | NR | (HR: 0.95; 95%CI: 0.84-1.07) | NA | 31-40 y | NA | NA | 18-30 y |
|  |  | 52 | NR | (HR: 0.97; 95%CI: 0.85-1.11) | NA | 41-50 y | NA | NA | 18-30 y |
|  |  | 52 | NR | (HR: 0.61; 95%CI: 0.51-0.74; p <0.001) | NA | 51-60 y | NA | NA | 18-30 y |
|  |  | 52 | NR | (HR: 0.93; 95%CI: 0.64-1.34) | NA | 60-64 y | NA | NA | 18-30 y |
|  | Wu, 2014 | 26 | 442 | (HR: 1.01; 95%CI: 0.99-1.03; p= 0.37) | NA | Age | NA | NA | Continuous |
|  | Yau, 2014 | 26 | 189 | (OR: 0.97; 95%CI: 0.95-0.99; p= 0.01) | NA | Age | NA | NA | Continuous |
|  | Yen, 2009 | 52 | 51 | (OR: 1.13; 95%CI: 1.01-1.22; p <0.05) | NA | NA | NA | NA | Continuous |
| Sex | Akincigil, 2007 | 16 | 2186 | NR | (OR: 0.91; 95%CI: 0.79-1.03) | Male | NA | NR | Female |
|  |  | 33 | 922 | NR | (OR: 0.98; 95%CI: 0.80-1.19) | Male | NA | NR | Female |
|  | Bhattacharjee, 2020 | 16 | 4644 | (X^2^: 0.21; p= 0.65) | (OR: 1.03; 99%CI: 0.86-1.23; p= 0.65) | Female | NA | NR | Male |
|  |  | 50 | 3584 | (X^2^: 0.61; p= 0.44) | (OR: 1.05; 99%CI: 0.89-1.25; p= 0.435) | Female | NA | NR | Male |
|  | Chen, 2010 | 12 | 4102 | (OR: 0.94; 95%CI: 0.82-1.08) | NA | Male | Acute | NR | Female |
|  |  | 39 | 1921 | (OR: 0.79; 95%CI: 0.65-0.97) | NA | Male | Continuation | NR | Female |
|  | Cohen, 2004 | 14 | NR | (r: -0.655) | NA | Sex | NA | NA | NR |
|  | Donohue, 2004 | 26 | 6753 | (OR: 1.09; 95%CI: 1.00-1.19; p <0.05) | NA | Female | NA | NA | Male |
|  | Gerlach, 2019 | 52 | 190 | NR | (OR: 0.62; 95% CI: 0.18-2.17; p= 0.45) | NA | NA | NR | Male |
|  | Holvast, 2019 | 52 | 878 | (OR: 0.91; 95% CI: 0.62-1.33; p= NS) | NA | Female | NA | NA | Man |
|  | Kales, 2013 | 16 |  | NR | (OR: 1.64; 95%CI: 0.67-4.02; p= 0.28) | NA | AA female | Age, marital status, and education | AA male |
|  |  | 16 |  | NR | (OR: 2.55; 95%CI: 1.06-6.13; p= 0.037) | NA | AA male |  | White male |
|  |  | 16 |  | NR | (OR: 3.69; 95%CI: 1.57-8.67; p= 0.003) | NA | AA female |  | White female |
|  | Keeley, 2007 | 12 | 12 | (p= 0.50) | NA | Female | NA | NA | Male |
|  | Kogut, 2016 | 12 | 1983 | NR | (OR: 1.18; 95%CI: 0.94-1.48; p= 0.49) | NA | NA | Age, plan and regimen type, diabetes mellitus, office and psychiatric visits | Male |
|  | Lin, 1995 | 4 | 119 | (p= 0.58) | NA | NA | NA | NA | NR |
|  |  | 12 | 87 | (p= 0.53) | NA | NA | NA | NA | NR |
|  | Lin, 2010 | 52 | 2111615 | (Beta: 9.13; SE: 4.80) | NA | Male | NA | NA | Female |
|  | Liu, 2011 | 52 | 12689 | NR | (OR: 1.05; 95%CI: 1.00-1.10) | Female | NA | Antidepressant, gender, age, health plan type, residence, comorbid diseases, and prior medications | Male |
|  | McLaughlin, 2007 | 39 | 747 | (OR: 0.94; Wald CI: 0.87-1.02) | NA | Female | NA | Male | Male |
|  | Merrick, 2012 | 16 | 383 | NR | (OR: 1.40; 95%CI: 0.80-2.40) | Female | NA | Conc-Top 2 model: sex, age, white, primary diagnosis of major depression in episode, CCI, behavioural health comorbidity, initial regimen includes SSRI, psychiatric prescriber, and state | Male |
|  |  | 16 | 383 | NR | (OR: 1.40; 95%CI: 0.80-2.40) | Female | NA | Conc-Herfindahl model: sex, age, white, primarydiagnosis of major depression in episode, CCI, behavioural health comorbidity, initial regimen includes SSRI, psychiatric prescriber, and state | Male |
|  | Shin, 2020 | 12 | NR | (RR: 1.01; 95%CI: 1.00-1.02; p <0.05) | (RR: 0.95; 95%CI: 0.93-0.99; p <0.01) | Male | NA | Policy, gender, age, and type of index medication | Female |
|  |  | 26 | NR | (RR: 0.93; 95%CI: 0.91-0.96; p <0.01) | (RR: 0.96; 95%CI: 0.93-0.99; p <0.05) | Male | NA |  | Female |
|  | Stang, 2007 | 39 | 826 | (OR: 1.01; 95%CI: 0.841-1.212) | NA | Female | Both | NR | Male |
|  | Ten Doesschate, 2009 | 104 | 91 | (OR: 0.47; 95%CI: 0.16-1.41; p= 0.18) | (OR: 0.43; 95%CI: 0.11-1.66; p= 0.22) | NA | NA | Sex, personality, education level, no of previous episodes, severity of residual symptoms, severity of last episode, DAS-A and treatment factors | Male |
|  | Wu, 2012 | 52 | NR | (OR: 1.09; 95%CI: 0.91-1.31) | NA | Female | NA | NA | Male |
|  |  | 52 | NR | (HR: 1.21; 95%CI: 1.07-1.37; p <0.001) | NA | Female | NA | NA | Male |
|  | Yau, 2014 | 26 | 189 | (OR: 2.26; 95%CI: 1.09-4.69; p= 0.03) | NA | Female | NA | NA | Male |
|  | Yen, 2009 | 52 | 51 | (OR: 1.53; 95%CI: 0.24-9.93; p <0.05) | NA | NA | NA | NA | Male |
| Ethnicity | Bhattacharjee, 2020 | 16 | 4644 | (X^2^: 7.99; p= 0.01) | (OR: 1.30; 99%CI: 1.02-1.66; p= 0.005) | Others | NA | NR | White |
|  |  | 50 | 3584 | (X^2^: 18.79; p <0.001) | (OR: 1.49; 99%CI: 1.18-1.89; p <0.001) | Others | NA | NR | White |
|  | Gerlach, 2017 | 16 | 305 | (OR: 0.69; 95% CI: 0.48-0.99; p= 0.04) | NA | NA | NA | NR | White |
|  | Gerlach, 2019 | 52 | 190 | NR | (OR: 2.69; 95% CI: 1.30-5.57; p= 0.008) | NA | NA | NR | White |
|  | Kales, 2013 | 16 | 115 | NR | (OR: 2.47; 95%CI: 1.32-4.62; p= 0.005) | NA | AA | Age, marital status, and education | White |
|  | Kales, 2016 | 16 | 91 | NA | (OR: 3.20; 95%CI: 1.71-6.01; p <0.001) | NA | NA | Age and education | White |
|  | Keeley, 2007 | 12 | 12 | (X^2^: 1.80; DF: 2; p= 0.40) | NA | NH | NA | NA | NH |
|  |  | 52 | 2111615 | (Beta: 12.53; SE: 6.06; p <0.05) | NA | NH-white | NA | NA | Hispanic |
|  |  | 52 | 2111615 | (Beta: 2.90; SE: 8.49) | NA | NH-black | NA | NA | Hispanic |
|  |  | 52 | 2111615 | (Beta: 28.27; SE: 9.67; p <0.01) | NA | Other | NA | NA | Hispanic |
|  | Merrick, 2012 | 16 | 383 | NR | (OR: 2.40; 95%CI: 1.30-4.30; p <0.01) | White | NA | Conc-Top 2 model: sex, age, white, primary diagnosis of major depression in episode, CCI, behavioural health comorbidity, initial regimen includes SSRI, psychiatric prescriber, and state | NR |
|  |  | 16 | 383 | NR | (OR: 2.40; 95%CI: 1.30-4.30; p <0.01) | White | NA | Conc-Herfindahl model: sex, age, white, primary diagnosis of major depression in episode, CCI, behavioural health comorbidity, initial regimen includes SSRI, psychiatric prescriber, and state | NR |
|  | Wu, 2012 | 52 | NR | (OR: 0.60; 95%CI: 0.51-0.72) | NA | AA | NA | NA | Caucasian |
|  |  | 52 | NR | (HR: 1.47; 95%CI: 1.30-1.65) | NA | AA | NA | NA | Caucasian |
|  | Wu, 2014 | 26 | 442 | (HR: 1.36; 95%CI: 1.10-1.57; p= 0.03) | NA | Non-white | NA | NA | White |
| Income | Akincigil, 2007 | 16 | 2186 | NR | (OR: 1.22; 95%CI: 1.05-1.42) | $50K-$70K | NA | NR | <$50K |
|  |  | 16 | 2186 | NR | (OR: 1.30; 95%CI: 1.11-1.53) | > $70K | NA | NR | <$50K |
|  |  | 33 | 922 | NR | (OR: 1.25; 95%CI: 1.00-1.55) | $50K-$70K | NA | NR | <$50K |
|  |  | 33 | 922 | NR | (OR: 1.22; 95%CI: 0.95-1.54) | > $70K | NA | NR | <$50K |
|  | Holvast, 2019 | 52 | 878 | (OR: 1.01; 95% CI: 0.97-1.04; p= NS) | NA | High | NA | NA | Low |
|  | Kogut, 2016 | 52 | 2111615 | (Beta: -6.12; SE: 5.25) | NA | $20K-$40K | NA | NA | < $20K |
|  |  | 52 | 2111615 | (Beta: -6.04; SE: 7.31) | NA | $40K-$60K | NA | NA | < $20K |
|  |  | 52 | 2111615 | (Beta: -11.69; SE: 9.98) | NA | > $60K | NA | NA | < $20K |
|  | Nam-Ju, 2020 | 12 | 32224 | NR | (RR: 0.95; 95%CI: 0.92-0.99; p <0.05) | IC 4 | ES | Model 1: IC | IC 5 |
|  |  | 12 | 32224 | NR | (RR: 0.90; 95%CI: 0.86-0.93; p <0.05) | IC 3 | ES |  |  |
|  |  | 12 | 32224 | NR | (RR: 0.86; 95%CI: 0.83-0.89; p <0.05) | IC 2 | ES |  |  |
|  |  | 12 | 32224 | NR | (RR: 0.85; 95%CI: 0.82-0.88; p <0.05) | IC 1 | ES |  |  |
|  |  | 12 | 32224 | NR | (RR: 0.95; 95%CI: 0.91-0.98; p <0.05) | IC 4 | ES | Model 2: IC, demographic characteristics | IC 5 |
|  |  | 12 | 32224 | NR | (RR: 0.90; 95%CI: 0.87-0.94; p <0.05) | IC 3 | ES |  |  |
|  |  | 12 | 32224 | NR | (RR: 0.88; 95%CI: 0.84-0.91; p <0.05) | IC 2 | ES |  |  |
|  |  | 12 | 32224 | NR | (RR: 0.86; 95%CI: 0.83-0.89; p <0.05) | IC 1 | ES |  |  |
|  |  | 12 | 32224 | NR | (RR: 0.95; 95%CI: 0.91-0.98; p <0.05) | IC 4 | ES | Model 3: IC, demographic characteristics, clinical characteristics | IC 5 |
|  |  | 12 | 32224 | NR | (RR: 0.90; 95%CI: 0.87-0.94; p <0.05) | IC 3 | ES |  |  |
|  |  | 12 | 32224 | NR | (RR: 0.88; 95%CI: 0.84-0.92; p <0.05) | IC 2 | ES |  |  |
|  |  | 12 | 32224 | NR | (RR: 0.86; 95%CI: 0.83-0.89; p <0.05) | IC 1 | ES |  |  |
|  |  | 12 | 32224 | NR | (RR: 0.95; 95%CI: 0.91-0.99; p <0.05) | IC 4 | ES | Model 4: IC, demographic characteristics, clinical characteristics, medical use characteristics | IC 5 |
|  |  | 12 | 32224 | NR | (RR: 0.92; 95%CI: 0.88-0.95; p <0.05) | IC 3 | ES |  |  |
|  |  | 12 | 32224 | NR | (RR: 0.88; 95%CI: 0.85-0.92; p <0.05) | IC 2 | ES |  |  |
|  |  | 12 | 32224 | NR | (RR: 0.87; 95%CI: 0.83-0.90; p <0.05) | IC 1 | ES |  |  |
|  |  | 26 | 21535 | NR | (RR: 0.92; 95%CI: 0.87-0.96; p <0.05) | IC 4 | ES | Model 1: IC | IC 5 |
|  |  | 26 | 21535 | NR | (RR: 0.86; 95%CI: 0.82-0.90; p <0.05) | IC 3 | ES |  |  |
|  |  | 26 | 21535 | NR | (RR: 0.84; 95%CI: 0.79-0.88; p <0.05) | IC 2 | ES |  |  |
|  |  | 26 | 21535 | NR | (RR: 0.82; 95%CI: 0.78-0.86; p <0.05) | IC 1 | ES |  |  |
|  |  | 26 | 21535 | NR | (RR: 0.92; 95%CI: 0.87-0.96; p <0.05) | IC 4 | ES | Model 2: IC, demographic characteristics | IC 5 |
|  |  | 26 | 21535 | NR | (RR: 0.86; 95%CI: 0.82-0.91; p <0.05) | IC 3 | ES |  |  |
|  |  | 26 | 21535 | NR | (RR: 0.86; 95%CI: 0.82-0.91; p <0.05) | IC 2 | ES |  |  |
|  |  | 26 | 21535 | NR | (RR: 0.85; 95%CI: 0.80-0.88; p <0.05) | IC 1 | ES |  |  |
|  |  | 26 | 21535 | NR | (RR: 0.92; 95%CI: 0.87-0.96; p <0.05) | IC 4 | ES | Model 3: IC, demographic characteristics, clinical characteristics | IC 5 |
|  |  | 26 | 21535 | NR | (RR: 0.86; 95%CI: 0.83-0.91; p <0.05) | IC 3 | ES |  |  |
|  |  | 26 | 21535 | NR | (RR: 0.86; 95%CI: 0.82-0.91; p <0.05) | IC 2 | ES |  |  |
|  |  | 26 | 21535 | NR | (RR: 0.85; 95%CI: 0.80-0.89; p <0.05) | IC 1 | ES |  |  |
|  |  | 26 | 21535 | NR | (RR: 0.89; 95%CI: 0.85-0.94; p <0.05) | IC 4 | ES | Model 4: IC, demographic characteristics, clinical characteristics, medical use characteristics | IC 5 |
|  |  | 26 | 21535 | NR | (RR: 0.87; 95%CI: 0.83-0.92; p <0.05) | IC 3 | ES |  |  |
|  |  | 26 | 21535 | NR | (RR: 0.87; 95%CI: 0.82-0.92; p <0.05) | IC 2 | ES |  |  |
|  |  | 26 | 21535 | NR | (RR: 0.86; 95%CI: 0.81-0.91; p <0.05) | IC 1 | ES |  |  |
|  |  | 12 | 32224 | NR | (RR: 0.89; 95%CI: 0.84-0.94; p <0.05) | IC 4 | ES | Model 1: IC | IC 5 |
|  |  | 12 | 32224 | NR | (RR: 0.84; 95%CI: 0.80-0.88; p <0.05) | IC 3 | ES |  |  |
|  |  | 12 | 32224 | NR | (RR: 0.82; 95%CI: 0.77-0.86; p <0.05) | IC 2 | ES |  |  |
|  |  | 12 | 32224 | NR | (RR: 0.90; 95%CI: 0.85-0.95; p <0.05) | IC 1 | ES |  |  |
|  |  | 12 | 32224 | NR | (RR: 0.94; 95%CI: 0.89-0.98; p <0.05) | IC 4 | ES | Model 2: IC, demographic characteristics (gender, onset age, area, type of insurance, type of employee insurance) | IC 5 |
|  |  | 12 | 32224 | NR | (RR: 0.90; 95%CI: 0.86-0.95; p <0.05) | IC 3 | ES |  |  |
|  |  | 12 | 32224 | NR | (RR: 0.89; 95%CI: 0.84-0.94; p <0.05) | IC 2 | ES |  |  |
|  |  | 12 | 32224 | NR | (RR: 0.95; 95%CI: 0.91-1.01) | IC 1 | ES |  |  |
|  |  | 12 | 32224 | NR | (RR: 0.94; 95%CI: 0.89-0.98; p <0.05) | IC 4 | ES | Model 3: IC, demographic characteristics, clinical characteristics | IC 5 |
|  |  | 12 | 32224 | NR | (RR: 0.90; 95%CI: 0.85-0.95; p <0.05) | IC 3 | ES |  |  |
|  |  | 12 | 32224 | NR | (RR: 0.89; 95%CI: 0.84-0.94; p <0.05) | IC 2 | ES |  |  |
|  |  | 12 | 32224 | NR | (RR: 0.95; 95%CI: 0.91-1.01) | IC 1 | ES |  |  |
|  |  | 12 | 32224 | NR | (RR: 0.94; 95%CI: 0.89-0.99; p <0.05) | IC 4 | ES | Model 4: IC, demographic characteristics, clinical characteristics, medical use characteristics | IC 5 |
|  |  | 12 | 32224 | NR | (RR: 0.89; 95%CI: 0.84-0.94; p <0.05) | IC 3 | ES |  |  |
|  |  | 12 | 32224 | NR | (RR: 0.87; 95%CI: 0.82-0.92; p <0.05) | IC 2 | ES |  |  |
|  |  | 12 | 32224 | NR | (RR: 0.91; 95%CI: 0.86-0.96; p <0.05) | IC 1 | ES |  |  |
|  |  | 26 | 21535 | NR | (RR: 0.88; 95%CI: 0.83-0.94; p <0.05) | IC 4 | ES | Model 1: IC | IC 5 |
|  |  | 26 | 21535 | NR | (RR: 0.82; 95%CI: 0.76-0.88; p <0.05) | IC 3 | ES |  |  |
|  |  | 26 | 21535 | NR | (RR: 0.80; 95%CI: 0.75-0.86; p <0.05) | IC 2 | ES |  |  |
|  |  | 26 | 21535 | NR | (RR: 0.87; 95%CI: 0.82-0.93; p <0.05) | IC 1 | ES |  |  |
|  |  | 26 | 21535 | NR | (RR: 0.96; 95%CI: 0.90-1.02) | IC 4 | ES | Model 2: IC, demographic characteristics | IC 5 |
|  |  | 26 | 21535 | NR | (RR: 0.91; 95%CI: 0.85-0.97; p <0.05) | IC 3 | ES |  |  |
|  |  | 26 | 21535 | NR | (RR: 0.92; 95%CI: 0.85-0.97; p <0.05) | IC 2 | ES |  |  |
|  |  | 26 | 21535 | NR | (RR: 0.96; 95%CI: 0.90-1.02) | IC 1 | ES |  |  |
|  |  | 26 | 21535 | NR | (RR: 0.96; 95%CI: 0.90-1.02) | IC 4 | ES | Model 3: IC, demographic characteristics, clinical characteristics | IC 5 |
|  |  | 26 | 21535 | NR | (RR: 0.91; 95%CI: 0.84-0.97; p <0.05) | IC 3 | ES |  |  |
|  |  | 26 | 21535 | NR | (RR: 0.92; 95%CI: 0.85-0.97; p <0.05) | IC 2 | ES |  |  |
|  |  | 26 | 21535 | NR | (RR: 0.96; 95%CI: 0.90-1.02) | IC 1 | ES |  |  |
|  |  | 26 | 21535 | NR | (RR: 0.96; 95%CI: 0.88-1.02) | IC 4 | ES | Model 4: IC, demographic characteristics, clinical characteristics, medical use characteristics | IC 5 |
|  |  | 26 | 21535 | NR | (RR: 0.91; 95%CI: 0.84-0.98; p <0.05) | IC 3 | ES |  |  |
|  |  | 26 | 21535 | NR | (RR: 0.91; 95%CI: 0.84-0.98; p <0.05) | IC 2 | ES |  |  |
|  |  | 26 | 21535 | NR | (RR: 0.90; 95%CI: 0.83-0.97; p <0.05) | IC 1 | ES |  |  |
| Education | Gerlach, 2019 | 52 | 190 | NR | (OR: 0.91; 95% CI: 0.58-1.42; p= 0.67) | NA | NA | NR | High school or below |
|  | Keeley, 2007 | 12 | 12 | (p= 0.80) | NA | NA | NA | NA | NA |
|  | Lin, 1995 | 4 | 119 | (p= 0.98) | NA | NA | NA | NA | NR |
|  |  | 12 | 87 | (p= 0.32) | NA | NA | NA | NA | NR |
|  | Ten Doesschate, 2009 | 104 | 91 | (OR: 3.00; 95%CI: 0.92-9.77; p= 0.07) | (OR: 4.75; 95%CI: 1.05-21.43; p= 0.04) | NA | NA | Sex, personality, education level, no of previous episodes, severity of residual symptoms, severity of last episode, DAS-A score and treatment factors | Other education level |
|  | Yen, 2009 | 52 | 51 | (OR: 1.09; 95%CI: 0.87-1.36; p <0.05) | NA | NA | NA | NA | Low |
| Civil status | Gerlach, 2019 | 52 | 190 | NR | (OR: 1.84; 95% CI: 1.16-2.92; p= 0.009) | NA | NA | NR | With spouse/partner |
|  | Holma, 2010 | 260 | 79 | NR | (OR: 3.13; 95% CI: 1.10-9.09; p= 0.032) | NA | NA | Sex, and age | Not living alone |
|  | Kales, 2016 | 16 | 91 | NA | (OR: 2.02; 95%CI: 1.20-3.38; p= 0.008) | NA | NA | Age and education | Partner or spouse |
|  | Ten Doesschate, 2009 | 104 | 91 | (OR: 1.04; 95%CI: 0.41-2.62; p= 0.94) | NA | NA | NA | NA | Not alone |
| Employment | Keeley, 2007 | 12 | 12 | (p= 0.80) | NA | Part-time | NA | NA | Full-time |
|  | Ten Doesschate, 2009 | 104 | 91 | (OR: 0.66; 95%CI: 0.27-1.61; p= 0.36) | NA | NA | NA | NA | No presence |
| AA: Afro-American; CCI: Charlson Comorbid Index score; DF: degrees of freedom; ES: employee subscribers; IC: income class; HR: hazard ratio; NR: not report; NA: not apply; OR: odds ratio | | | | | | | | | |

Supplementary Table 4. Main findings obtained in the included studies. Clinical factors – Implementation

| Predictor factor | Author, year | Follow-up (weeks) | N adherence patients | Univariate and other statistics | Multivariate statistics | Group | Cohort | Model adjustment | Reference |
| --- | --- | --- | --- | --- | --- | --- | --- | --- | --- |
| Implementation | | | | | | | | | |
| PC | Akincigil, 2007 | 16 | 2186 | (OR: 0.99; 95%CI: 0.86-1.14) | NA | AD | NA | NA | No |
|  |  | 33 | 922 | (OR: 1.02; 95%CI: 0.83-1.24) | NA | AD | NA | NA | No |
|  | Chen, 2010 | 12 | 4102 | (OR: 0.62; 95%CI: 0.45-0.86; p <0.05) | NA | SUD | Acute | NA | No |
|  |  | 12 | 4102 | (OR: 1.11; 95%CI: 0.87-1.41) | NA | AD | Acute | NA | No |
|  |  | 39 | 1921 | (OR: 0.75; 95%CI: 0.45-1.24) | NA | SUD | Continuation | NA | No |
|  |  | 39 | 1921 | (OR: 1.08; 95%CI: 0.77-1.50) | NA | AD | Continuation | NA | No |
|  | Gerlach, 2019 | 52 | 79 | NR | (OR: 0.62; 95%CI: 0.35-1.10; p= 0.10) | PTSD | NA | NR | No |
|  |  | 52 | 28 | NR | (OR: 1.27; 95%CI: 0.70-2.31; p= 0.44) | SUD | NA | NR | NI |
|  |  | 52 | 41 | NR | (OR: 1.18; 95%CI: 0.68-2.05; p= 0.57) | AD | NA | NR | No |
|  | Keeley, 2007 | 12 | 12 | (p= 0.80) | NA | Somatoform comorbidity | NA | NA | No |
|  | Lin, 2010 | 52 | 2111615 | (Beta: -6.50; SE: 7.24) | NA | Psychotic disorders | NA | NA | No |
|  |  | 52 | 2111615 | (Beta: -1.43; SE: 4.38) | NA | AD | NA | NA | No |
|  | Liu, 2011 | 52 | 12689 | NR | (OR: 0.90; 95%CI: 0.83-0.95) | Fibromyalgia | NA | Antidepressant, gender, age, health plan type, residence, comorbid diseases, and prior medications | No |
|  |  | 52 | 12689 | NR | (OR: 1.23; 95%CI: 1.11-1.36) | SD | NA |  | No |
|  |  | 52 | 12689 | NR | (OR: 0.75; 95%CI: 0.66-0.85) | AUD | NA |  | No |
|  |  | 52 | 12689 | NR | (OR: 0.66; 95%CI: 0.56-0.78) | SUD | NA |  | No |
|  | Wu, 2012 | 52 | NI | (OR: 1.55; 95%CI: 1.27-1.90; p <0.001) | NA | AD | NA | NA | No |
| MC | Akincigil, 2007 | 16 | 2186 | (OR: 1.05; 95%CI: 0.89-1.25) | NA | Cancer | NA | NA | No |
|  |  | 33 | 922 | (OR: 0.92; 95%CI: 0.73-1.15) | NA | Cancer | NA | NA | No |
|  |  | 16 | 2186 | (OR: 0.82; 95%CI: 0.67-0.99; p <0.05) | NA | Headache/migraine | NA | NA | No |
|  |  | 33 | 922 | (OR: 0.79; 95%CI: 0.59-1.04; p <0.05) | NA | Headache/migraine | NA | NA | No |
|  |  | 16 | 2186 | (OR: 0.98; 95%CI: 0.82-1.16) | NA | CVD/diabetes (= 1) | NA | NA | No |
|  |  | 16 | 2186 | (OR: 0.65; 95%CI: 0.49-0.86; p <0.05) | NA | CVD/diabetes (≥ 2) | NA | NA | No |
|  |  | 33 | 922 | (OR: 1.10; 95%CI: 0.89-1.38; p <0.05) | NA | CVD/diabetes (= 1) | NA | NA | No |
|  |  | 33 | 922 | (OR: 0.91; 95%CI: 0.62-1.34; p <0.05) | NA | CVD/diabetes (≥ 2) | NA | NA | No |
|  | Bhattacharjee, 2020 | 16 | 293 | NA | (OR: 1.39; 99% CI: 0.92-2.10; p= 0.041) | PD | NA | NR | No |
|  |  | 50 | 232 | NA | (OR: 1.36; 99% CI: 0.93-1.99; p= 0.039) | PD | NA | NR | No |
|  | Gerlach, 2019 | 52 | 79 | NR | (OR: 1.30; 95%CI: 1.13-1.49; p= 0.001) | CCI (≥ 1) | NA | NR | NR |
|  | Kales, 2016 | 16 | 91 | NA | (OR: 1.31; IC 95%: 1.11-1.54; p= 0.002) | NA | NA | Age, education and illness burden variables | CCI (0) |
|  | Keeley, 2007 | 12 | 12 | (p= 0.60) | NA | Chronic Comorbidities | NA | NA | No |
|  | Liu, 2011 | 52 | 12689 | NR | (OR: 0.89; 95%CI: 0.84-0.95) | Headaches | NA | Antidepressant, gender, age, health plan type, residence, comorbid diseases, and prior medications | No |
|  |  | 52 | 12689 | NR | (OR: 0.94; 95%CI: 0.89-0.99) | Low back pain | NA |  | No |
|  | Merrick, 2012 | 16 | 383 | NR | (OR: 1.4; 95%CI: 0.8-1.8) | CCI (= 2) | NA | Conc-Top 2 model: sex, age, white, primary diagnosis of MD episode, CCI score, behavioural health comorbidity, initial regimen includes SSRI, psychiatric prescriber, and state | CCI (0-1) |
|  |  | 16 | 383 | NR | (OR: 1.4; 95%CI: 0.8-1.8) | CCI (= 2) | NA | Conc-Herfindahl model: sex, age, white, primary diagnosis of major depression in episode, CCI score, behavioural health comorbidity, initial regimen includes SSRI, psychiatric prescriber, and state | CCI (0-1) |
|  | Wu, 2012 | 52 | NI | (OR: 1.02; 95%CI: 0.83-1.25) | NA | MC EI (= 1) | NA | NA | No |
|  |  | 52 | NI | (OR: 1.30; 95%CI: 1.03-1.63; p <0.05) | NA | MC EI (= 2) | NA | NA | No |
|  |  | 52 | NI | (OR: 1.34; 95%CI: 1.06-1.69; p <0.05) | NA | MC EI (≥ 3) | NA | NA | No |
|  |  | 52 | NI | (HR: 1.10; 95%CI: 0.94-1.28) | NA | MC EI (≥ 3) | NA | NA | No |
| Severity | Cohen, 2004 | 14 | NR | (Other: -0.146; p= 0.28) | NA | NA | NA | NA | Continuous |
|  | Lin, 1995 | 4 | 119 | (p= 0.15) | NA | NA | NA | NA | NR |
|  |  | 12 | 87 | (p= 0.33) | NA | NA | NA | NA | NR |
|  | Merrick, 2012 | 16 | 383 | NR | (OR: 1.2; 95%CI: 0.8-1.8) | Primary diagnosis of MD ep. | NA | Conc-Top 2 model: sex, age, white, primary diagnosis of MD episode, CCI score, behavioural health comorbidity, initial regimen includes SSRI, psychiatric prescriber, and state | No |
|  |  | 16 | 383 | NR | (OR: 1.2; 95%CI: 0.8-1.8) | Primary diagnosis of MD ep. | NA |  | No |
|  | Sirey, 2001 | 12 | 96 | NA | (OR: 1.22; IC 95%: 0.99-1.48; p= 0.05) | Severity of illness | NA | Age | Continuous |
| Subtype | Cohen, 2004 | 14 | NR | (Other: 0.865; p= 0.31) | NA | NA | NA | NA | Continuous |
|  | Yen, 2009 | 52 | 51 | (OR: 1242; 95%CI: 0.17-9.34; p <0.05) | NA | NA | NA | NA | Continuous |
|  | Donohue,2004 | 26 | 11306 | (OR: 1.01; 95%CI: 0.92-1.10) | NA | MDD | NA | NA | Other |
| PE | Cohen, 2004 | 14 | NR | (Other: -0.164; p= 0.31) | NA | NA | NA | NA | No |
| AUD: alcohol use/abuse disorder; CCI: Charlson Comorbid Index score; DF: degrees of freedom; EI: Elixhauser Index score; HR: hazard ratio; PC: psychiatric comorbidities; MC: medical comorbidities; MDD: Major Depression Disorder; NR: not report; NA: not apply; OR: odds ratio; PE: previous episodes; SE: standard error; SD: sleep disorder; SUD: substance use/abuse disorder | | | | | | | | | |

Supplementary Table 5. Main findings obtained in the included studies. Sociodemographic factors – Discontinuation/Initiation

| Predictor factor | Author, year | Follow-up (weeks) | N adherence patients | Univariate and other statistics | Multivariate statistics | Group | Model adjustment | Reference |
| --- | --- | --- | --- | --- | --- | --- | --- | --- |
| Discontinuation | | | | | | | | |
| Age | Demyttenaere, 2001 | 24 | 144 | (p= 0.39) | NA | Age | NA | Continuous |
|  | Ereshefsky, 2010 | 26 | 8609 | NR | (HR: 0.86; 95%CI: 0.84-0.88; p <0.05) | 35-49 y | Antidepressant, age, physician’s specialty, healthcare plan, payer, residence, abuse, and drugs prescribed | 18-34 y |
|  |  | 26 | 8609 | NR | (HR: 0.92; 95%CI: 0.90-0.95; p <0.05) | 56-64 y |  | 18-34 y |
|  | Goethe, 2007 | 12 | 91 | NR | (OR: 0.98; 95%CI: 0.96-1.00; p= 0.02) | NA | NA | Continuous |
|  | Holvast, 2019 | 42 | 219 | (HR: 1.04; 95%CI: 1.02-1.06; p <0.001) | NA | NA | NA | Continuous |
|  | Hung, 2011 | 16 | NR | (HR: 0.97; 95%CI: 0.94-0.997; p= 0.03) | NA | NR | NR | NR |
|  | Keeley, 2000 | 12 | NR | (p= 0.28) | NA | NA | NA | NA |
|  | Keeley, 2007 | 12 | NR | (p= 0.8) | NA | NA | NA | NA |
|  | Liu, 2010 | 52 | NR | (OR: 0.88; 95%CI: 0.76-1.01) | NA | 26-35 y | NA | 18-25 y |
|  |  | 52 | NR | (OR: 0.73; 95%CI: 0.64-0.83; p <0.05) | NA | 36-45 y | NA | 18-25 y |
|  |  | 52 | NR | (OR: 0.67; 95%CI: 0.59-0.75; p <0.05) | NA | 46-55 y | NA | 18-25 y |
|  |  | 52 | NR | (OR: 0.61; 95%CI: 0.54-0.70; p <0.05) | NA | 56-64 y | NA | 18-25 y |
|  | Liu, 2011 | 52 | 12689 | NR | (HR: 0.85; 95%CI: 0.82-0.87; p <0.05) | 36-45 y | Antidepressant, Female gender, Age (y), Health plan type, Geographic region of residence, Comorbid diseases, and Prior medications | 18-35 y |
|  |  | 52 | 12689 | NR | (HR: 0.80; 95%CI: 0.78-0.82; p <0.05) | 46-55 y |  | 18-35 y |
|  |  | 52 | 12689 | NR | (HR: 0.72; 95%CI: 0.70-0.74; p <0.05) | 56-64 y |  | 18-35 y |
|  | Milea, 2010 | 4 | NR | NR | (OR: 0.89; 95%CI: 0.85-0.92; p <0.05) | <18 y | NA | 18-39 y |
|  |  | 4 | NR | NR | (OR: 0.85; 95%CI: 0.83-0.87; p <0.05) | 40-64 y | NA | 18-39 y |
|  |  | 4 | NR | NR | (OR: 0.78; 95%CI: 0.62-0.99; p <0.05) | ≥65 y | NA | 18-39 y |
|  |  | 52 | NR | NR | (OR: 1.02; 95%CI: 0.99-1.05) | <18 y | NA | 18-39 y |
|  |  | 52 | NR | NR | (OR: 0.76; 95%CI: 0.75-0.77; p <0.05) | 40-64 y | NA | 18-39 y |
|  |  | 52 | NR | NR | (OR: 0.92; 95%CI: 0.76-1.12) | ≥65 y | NA | 18-39 y |
|  | Noh, 2022 | 26 | 4657 | (HR: 0.97; 95%CI: 0.97-0.98; p <0.05) | NA | NA | NA | NR |
|  | Olfson, 2006 | 4 | 216 | (X^2^: 0.60; DF: 1; p= 0.45) | (OR: 0.85; 95%CI: 0.56-1.30) | 18-44 y | Age, sex, ethnicity, and pretreatment mental health status. | >65 y |
|  |  | 4 | 166 | (X^2^: 0.40; DF: 1; p= 0.51) | (OR: 0.94; 95%CI: 0.58-1.52) | 45-64 y |  | >65 y |
|  |  | 4 | 85 | (X^2^: 0.10; DF: 1; p= 0.79) | NA | ≥65 y | NA | >65 y |
|  | Vlahiotis, 2011 | 26 | 9093 | (OR: 0.77; 95%CI: 0.70-0.85; p <0.001) | NA | 26-40 y | NA | 18-25 y |
|  |  | 26 | 9093 | (OR: 0.59; 95%CI: 0.54-0.65; p <0.001) | NA | 41-55 y | NA | 18-25 y |
|  |  | 26 | 9093 | (OR: 0.49; 95%CI: 0.43-0.55; p <0.001) | NA | 56-64 y | NA | 18-25 y |
|  | Woolley, 2010 | 12 | NR | NR | (OR: 0.98; 95%CI: 0.96-1.00) | NA | Age, sex, education, and experiencing extremely bothersome | Continuous |
|  | Wu, 2013 | 26 | 4480 | (HR: 0.88; 95% CI: 0.85-0.91; p <0.05) | NA | 45-64 y | NA | 18-44 y |
|  |  | 26 | 4480 | (HR: 0.79; 95% CI: 0.75-0.83; p <0.05) | NA | ≥65 y | NA | 18-44 y |
| Sex | Demyttenaere, 2001 | 24 | 144 | (p= 0.56) | NA | Sex | NA | NR |
|  | Goethe, 2007 | 12 | 91 | (OR: 1.68; 95%CI: 1.00-2.81; p= 0.048) | (OR: 1.49; 95%CI: 1.05-2.10; p= 0.03) | Male | NA | Female |
|  | Holvast, 2019 | 42 | 219 | (HR: 0.94; 95%CI: 0.63-1.40) | NA | Female | NA | Male |
|  | Keeley, 2007 | 12 | NR | (p= 1.00) | NA | Female | NA | Male |
|  | Milea, 2010 | 4 | NR | NR | (OR: 1.00; 95%CI: 0.98-1.02) | Female | NA | Male |
|  |  | 52 | NR | NR | (OR: 0.95; 95%CI: 0.94-0.97; p <0.05) | Female | NA | Male |
|  | Olfson, 2006 | 4 | 347 | (X^2^: 0.30; DF: 1; p= 0.59) | (OR: 1.13; 95%CI: 0.77-1.66) | Female | Age, sex, ethnicity, and pretreatment mental health status. | Male |
|  | Vlahiotis, 2011 | 26 | 9093 | (OR: 1.07; 95%CI: 1.00-1.14; p= 0.038) | NA | Male | NA | Female |
|  | Wu, 2013 | 26 | 4480 | (HR: 0.88; 95% CI: 0.86-0.91; p <0.05) | NA | Male | NA | Female |
| Ethnicity | Keeley, 2000 | 12 | NR | (p= 0.80) | NA | NA | NA | NA |
|  | Keeley, 2007 | 12 | NR | (p= 0.30) | NA | NH-white | NA | NH-AA and Hispanic |
|  | Olfson, 2006 | 4 | 366) | (X^2^: 4.10; DF: 1; p <0.05) | NA | White | Age, sex, ethnicity, and pretreatment mental health status. | White |
|  |  | 4 | 29 | (X^2^: 0.20; DF: 1; p= 0.63) | (OR: 0.76; 95%CI: 0.38-1.54) | Black |  | White |
|  |  | 4 | 63 | (X^2^: 4.30; DF: 1; p <0.04) | (OR: 0.58; 95%CI: 0.36-0.94) | Hispanic |  | White |
|  |  | 4 | 9 | (X^2^: 0.40; DF: 1; p= 0.55) | (OR: 0.65; 95%CI: 0.20-2.14) | American Indian, Alaska native, and Asian or Pacific Islander |  | White |
| Income | Holvast, 2019 | 42 | 219 | (HR: 1.00; 95%CI: 0.96-1.05) | NA | High | NA | Low |
|  | Olfson, 2006 | 4 | 163 | (X^2^: 7.70; DF: 1; p= 0.006) | (OR: 0.64; 95%CI: 0.41-0.99) | Low income | Age, sex, ethnicity, and pretreatment mental health status. | High income |
|  |  | 4 | 155 | (X^2^: 1.30; DF: 1; p= 0.25) | (OR: 0.98; 95%CI: 0.62-1.56) | Medium income |  | High income |
|  |  | 4 | 149 | (X^2^: 1.90; DF: 1; p= 0.18) | NA | High income | NA | High income |
| Employment | Goethe, 2007 | 12 | 91 | (RR: 0.67; 95%CI: 0.48-0.94; p= 0.01) | NA | NA | NA | Unemployed |
|  | Keeley, 2007 | 12 | NR | (p= 0.09) | NA | Part-time | NA | Full-time |
|  | Olfson, 2006 | 4 | 208 | (X^2^: 0.10; DF: 1; p= 0.77) | NA | Unemployed | NA | Employed |
|  |  | 4 | 259 | NR | (OR: 0.90; 95%CI: 0.61-1.32) | Employed | NA | Employed |
| Education | Keeley, 2007 | 12 | NR | (p= 0.50) | NA | NA | NA | NA |
|  | Olfson, 2006 | 4 | 126 | (X^2^: 6.50; DF: 1; p <0.02) | (OR: 0.53; 95%CI: 0.35-0.79) | <12 y | Age, sex, ethnicity, and pretreatment mental health status. | >12 y |
|  |  | 4 | 137 | (X^2^: 1.10; DF: 1; p= 0.29) | (OR: 0.64; 95%CI: 0.42-0.92) | 12 y |  | >12 y |
|  |  | 4 | 204 | (X^2^: 8.90; DF: 1; p= 0.003) | NA | >12 y | NA | >12 y |
|  | Woolley, 2010 | 12 | NR | NR | (OR: 1.26; 95%CI: 0.72-2.20) | < High school graduate and/or received general educational degree | Age, sex, education, and experiencing extremely bothersome | > High school graduate and/or received general educational degree |
| Civil status | Olfson, 2006 | 4 | 263 | (X^2^: 3.20; DF: 1; p= 0.08) | NA | Married | NA | Married |
|  |  | 4 | 73 | (X^2^: 1.00; DF: 1; p= 0.33) | (OR: 0.71; 95%CI: 0.43-1.18) | Never married | Age, sex, ethnicity, and pretreatment mental health status. | Married |
|  |  | 4 | 79 | (X^2^: 1.84; DF: 1; p= 0.18) | (OR: 0.66; 95%CI: 0.42-1.04) | Divorced or separated |  | Married |
|  |  | 4 | 52 | (X^2^: 0.10; DF: 1; p= 0.73) | (OR: 0.94; 95%CI: 0.51-1.72) | Widowed |  | Married |
| Initiation | | | | | | | | |
| Age | Holvast, 2019 | 2 | 545 | (OR: 1.00; 95% CI: 0.95-1.06) | NA | NA | NA | Continuous |
| Sex | Holvast, 2019 | 2 | 545 | (OR: 1.63; 95% CI: 0.69-3.86) | NA | Female | NA | Male |
| Income | Holvast, 2019 | 2 | 545 | (OR: 1.04; 95% CI: 0.96-1.13) | NA | High | NA | Low |
| AA: Afro-American; CCI: Charlson Comorbid Index score; DF: degrees of freedom; ES: employee subscribers; IC: income class; HR: hazard ratio; NH: Non-Hispanic; NR: not report; NA: not apply; OR: odds ratio | | | | | | | | |

Supplementary Table 6. Main findings obtained in the included studies. Clinical factors – Discontinuation/Initiation/Suboptimal implementation

| Predictor factor | Author, year | Follow-up (weeks) | N adherence patients | Univariate and other statistics | Multivariate statistics | Group | Model adjustment | Reference |
| --- | --- | --- | --- | --- | --- | --- | --- | --- |
| Discontinuation | | | | | | | | |
| PC | Ereshefsky, 2010 | 26 | 8609 | NR | (HR: 1.13; 95%CI: 1.08-1.17) | SUD | Type of antidepressant, age, physician’s specialty, healthcare plan, payer, residence, abuse, and drugs prescribed | No |
|  |  | 26 | 8609 | NR | (HR: 1.14; 95%CI: 1.06-1.22) | AUD |  | No |
|  | Goethe, 2007 | 12 | 406 | NR | (OR: 1.67; 95%CI: 0.99-2.81; p= 0.053) | Anxiety symptoms | NR | No |
|  | Holvast, 2019 | 42 | 219 | (HR: 1.08; 95%CI: 0.94-1.25) | NA | No. chronic somatic diseases | NA | Continuous |
|  |  | 42 | 219 | (HR: 1.59; 95%CI: 0.91-2.79) | NA | PC | NA | Continuous |
|  | Hung, 2011 | 24 | 85 | (OR: 0.40; 95%CI: 0.16-0.97; p= 0.04) | NA | Panic/agoraphobia | NA | No |
|  |  | 24 | 85 | (X^2^: 4.46; DF: 1; p= 0.04) | NA | Panic/agoraphobia | NA | No |
|  |  | 24 | 85 | (X^2^: 4.25; DF: 1; p= 0.04) | NA | PTSD | NA | No |
|  | Kelley, 2007 | 12 | NR | (p= 1.00) | NA | Somatoform comorbidity | NA | NA |
|  | Liu, 2011 | 52 | 12689 | NR | (HR: 0.97; 95%CI: 0.94-0.99) | AD | Antidepressant, gender, age, health plan type, residence, comorbid diseases, and prior medications | No |
|  |  | 52 | 12689 | NR | (HR: 0.91; 95%CI: 0.86-0.95) | SD |  | No |
|  |  | 52 | 12689 | NR | (HR: 1.17; 95%CI: 1.11-1.24) | AUD |  | No |
|  |  | 52 | 12689 | NR | (HR: 1.22; 95%CI: 1.14-1.31) | SUD |  | No |
|  | Milea, 2010 | 4 | NR | (OR: 1.06; 95%CI: 1.04-1.08; p <0.05) | NA | Psychosomatic comorbidity | NA | No |
|  |  | 52 | NR | (OR: 1.02; 95%CI: 1.00-1.03) | NA | Psychosomatic comorbidity | NA | No |
|  | Noh, 2022 | 26 | 30 | (HR: 1.03; 95%CI: 0.92-1.16) | (OR: 1.04; 95%CI: 0.92-1.17) | Mood disorder | Maternal age at delivery, monotherapy, TCA, SSRI, SNRI, others AD, combination therapy, medical aid recipients, residence, nulliparity, multifetal gestation, obstetric comorbidity index, depression relapse, PC, MC, outpatient visits, hospitalization due to psychiatric disorder, duration of ADs, prescribed by psychiatric specialists. | No |
|  |  | 26 | 269 | (HR: 0.95; 95%CI: 0.90-1.01) | (OR: 0.92; 95%CI: 0.87-0.98) | AD and stress-related disorder |  | No |
|  |  | 26 | 27 | (HR: 0.97; 95%CI: 0.84-1.11) | (OR: 1.17; 95%CI: 1.01-1.35) | SUD |  | No |
|  |  | 26 | 8 | (HR: 1.15; 95%CI: 0.94-1.40) | (OR: 1.15; 95%CI: 0.87-1.53) | Eating disorder |  | No |
|  |  | 26 | 10 | (HR: 0.92; 95%CI: 0.73-1.17) | (OR: 0.93; 95%CI: 0.73-1.18) | Personality disorder |  | No |
|  |  | 26 | 244 | (HR: 0.80; 95%CI: 0.75-0.85; p <0.05) | (OR: 0.83; 95%CI: 0.78-0.89) | SD |  | No |
|  |  | 26 | 38 | (HR: 0.69; 95%CI: 0.58-0.81; p <0.05) | (OR: 0.80; 95%CI: 0.68-0.95) | Schizophrenia-like disorder |  | No |
|  |  | 26 | 77 | (HR: 0.80; 95%CI: 0.73-0.88; p <0.05) | (OR: 0.91; 95%CI: 0.82-1.00) | Affective psychotic disorder |  | No |
|  | Vlahiotis, 2011 | 26 | 1303 | (OR: 1.09; 95%CI: 1.00-1.19; p= 0.001) | (OR: 1.10; 95%CI: 1.01-1.20) | AD | Other factors associated with health care costs in patients with MDD | No |
|  |  | 26 | 57 | (OR: 0.85; 95%CI: 0.58-1.25; p= 0.785) | NA | OCD | NA | No |
|  |  | 26 | 69 | (OR: 1.77; 95%CI: 1.29-2.42; p< 0.001) | (OR: 1.80; 95%CI: 1.33-2.45) | Bipolar disorder | NA | No |
|  | Wu, 2012 | 52 | NR | (HR: 1.04; 95%CI: 0.92-1.18) | NA | AD | NA | No |
|  | Wu, 2013 | 26 | 4480 | (HR: 0.88; 95%CI: 0.85-0.92; p <0.05) | NA | AD | NA | No |
|  |  | 26 | 4480 | (HR: 0.90; 95%CI: 0.86-0.93; p <0.05) | NA | SD | NA | No |
|  |  | 26 | 4480 | (HR: 1.21; 95%CI: 1.06-1.38; p <0.05) | NA | AUD | NA | No |
|  |  | 26 | 4480 | (HR: 1.02; 95%CI: 0.79-1.31) | NA | SUD | NA | No |
| MC | Hung, 2011 | 24 | 85 | (X^2^: 4.61; DF: 1; p= 0.03) | NA | Migraine | NA | No |
|  | Kelley, 2007 | 14 | 10 | (p= 0.32) | NA | Chronic comorbidities | NA | NA |
|  |  | 12 | NR | (p= 0.30) | NA | Chronic comorbidities | NA | NA |
|  | Liu, 2011 | 52 | 12689 | NR | (HR: 1.05; 95%CI: 1.02-1.09) | Headaches | Antidepressant, gender, age, health plan type, residence, comorbid diseases, and prior medications | No |
|  |  | 52 | 12689 | NR | (HR: 1.06; 95%CI: 1.04-1.09) | Low back pain |  | No |
|  | Milea, 2010 | 4 | NR | (OR: 0.97; 95%CI: 0.94-0.99; p <0.05) | NA | Somatic comorbidity | NA | No |
|  |  | 52 | NR | (OR: 0.94; 95%CI: 0.93-0.96; p <0.05) | NA | Somatic comorbidity | NA | No |
|  | Noh, 2022 | 26 | 38 | (HR: 0.68; 95%CI: 0.58-0.80; p <0.05) | (OR: 0.83; 95%CI: 0.70-0.99) | CD | Maternal age at delivery, monotherapy, TCA, SSRI, SNRI, others AD, combination therapy, medical aid recipients, residence, nulliparity, multifetal gestation, obstetric comorbidity index, depression relapse, PC, MC, outpatient visits, hospitalization due to psychiatric disorder, duration of ADs, prescribed by psychiatric specialists. | No |
|  |  | 26 | 17 | (HR: 0.67; 95%CI: 0.52-0.86; p <0.05) | (OR: 0.94; 95%CI: 0.73-1.22) | Diabetes |  | No |
|  |  | 26 | 17 | (HR: 0.66; 95%CI: 0.52-0.84; p <0.05) | (OR: 0.83; 95%CI: 0.65-1.05) | Epilepsy |  | No |
|  |  | 26 | NA | (HR: 0.86; 95%CI: 0.84-0.89; p <0.05) | (OR: 0.93; 95%CI: 0.90-0.97) | OCI |  | No |
|  | ten Doesschate, 2009 | 104 | 28 | (OR: 1.89; 95%CI: 0.38-9.54; p= 0.440) | NA | NA | NA | No |
|  | Vlahiotis, 2011 | 26 | 1601 | (OR: 1; 95%CI: 0.93-1.08; p= 0.239) | NA | CCI (1-2) | NA | CCI (0) |
|  |  | 26 | 179 | (OR: 1.15; 95%CI: 0.93-1.42; p= 0.239) | NA | CCI (3-5) | NA | CCI (0) |
|  |  | 26 | 80 | (OR: 0.75; 95%CI: 0.53-1.07; p= 0.239) | NA | CCI (≥ 6) | NA | CCI (0) |
|  | Wu, 2012 | 52 | NR | (HR: 0.99; 95%CI: 0.86-1.14) | NA | MC EI (= 1) | NA | No |
|  |  | 52 | NR | (HR: 1.08; 95%CI: 0.93-1.26) | NA | MC EI (= 2) | NA | No |
|  | Wu, 2013 | 26 | 4480 | (HR: 0.97; 95%CI: 0.94-1.01) | NA | CCI (1-2) | NA | CCI (0) |
|  |  | 26 | 4480 | (HR: 0.96; 95%CI: 0.92-1.00) | NA | CCI (≥ 3) | NA | CCI (0) |
|  | Wu, 2014 | 26 | 442 | (HR: 0.99; 95%CI: 0.77-1.17; p= 0.920) | NA | CCI (≥ 1) | NA | CCI 0 |
| PE | ten Doesschate, 2009 | 104 | 28 | (OR: 1.75; 95%CI: 0.98-3.12; p= 0.059) | (OR: 1.82; 95%CI: 0.89-3.73; p= 0.10) | NA | Sex, personality, education level, no of previous episodes, severity of residual, severity of last episode, DAS-A score, and treatment factors | Continuous |
|  | Wu, 2014 | 26 | 442 | (HR: 0.94; 95%CI: 0.34-1.32; p= 0.625) | NA | Previous diagnosis | NA | No |
| Severity | Hung, 2011 | 24 | 85 | (HR: 0.61; 95%CI: 0.39-0.94; p= 0.03) | NA | Chronic MDD | NA | No |
|  |  | 24 | 85 | (OR: 0.40; 95%CI: 0.20-0.81; p= 0.01) | NA | Chronic MDD | NA | No |
|  |  | 24 | 85 | (X^2^: 4.75; DF: 1; p= 0.03) | NA | Chronic MDD | NA | No |
|  | ten Doesschate, 2009 | 104 | 28 | (OR: 1.14; 95%CI: 0.96-1.36; p= 0.122) | (OR: 1.15; 95%CI: 0.92-1.43; p= 0.22) | NA |  | Continuous |
| Initiation | | | | | | | | |
| PC | Holvast, 2019 | 2 | 545 | (OR: 1.23; 95%CI: 0.91-1.67) | NA | Antidepressant | NA | Continuous |
|  |  | 2 | 545 | (OR: 0.67; 95%CI: 0.32-1.38) | NA | Antidepressant | NA | Continuous |
| Suboptimal implementation | | | | | | | | |
| PC | Holvast, 2019 | 52 | 878 | (OR: 1.07; 95%CI: 0.96-1.01) | NA | Antidepressant | NA | Continuous |
|  |  | 52 | 878 | (OR: 1.21; 95%CI: 0.62-1.33) | NA | Antidepressant | NA | Continuous |
| AD: anxiety disorders; AUD: alcohol use/abuse disorder; CCI: Charlson Comorbid Index score; CD: Cardiovascular Disease; DF: degrees of freedom; EI: Elixhauser Index score; HR: hazard ratio; PC: psychiatric comorbidities; MC: medical comorbidities; MDD: Major Depression Disorder; NR: not report; NA: not apply; OCD: obsessive-compulsive disorder; OCI: Obstetric comorbidity index; OR: odds ratio; PE: previous episodes; PTSD: post-traumatic stress disorder; SD: sleep disorder; SUD: substance use/abuse disorder. | | | | | | | | |

Supplementary Figure 1. Forest plot. Age, 25-50 years - Implementation; 39-52 weeks


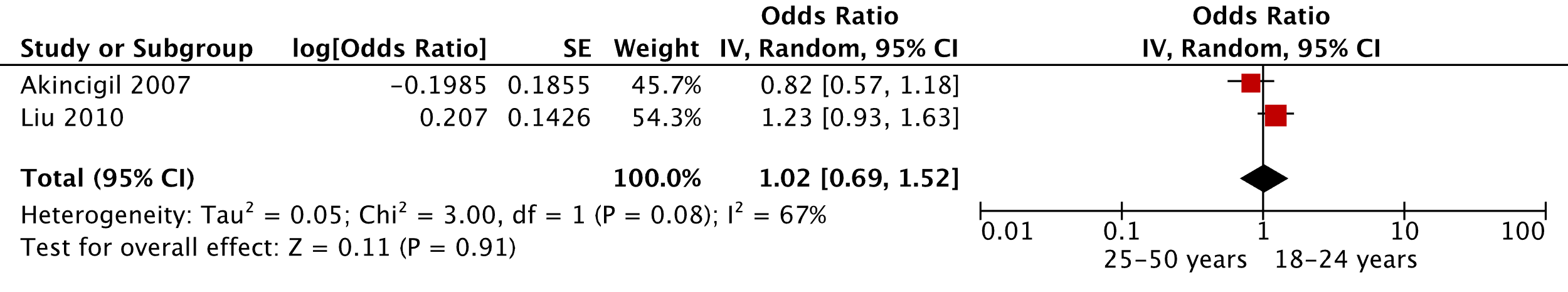


Supplementary Figure 2. Forest plot. Age, 35-49 years - Implementation; 16-52 weeks


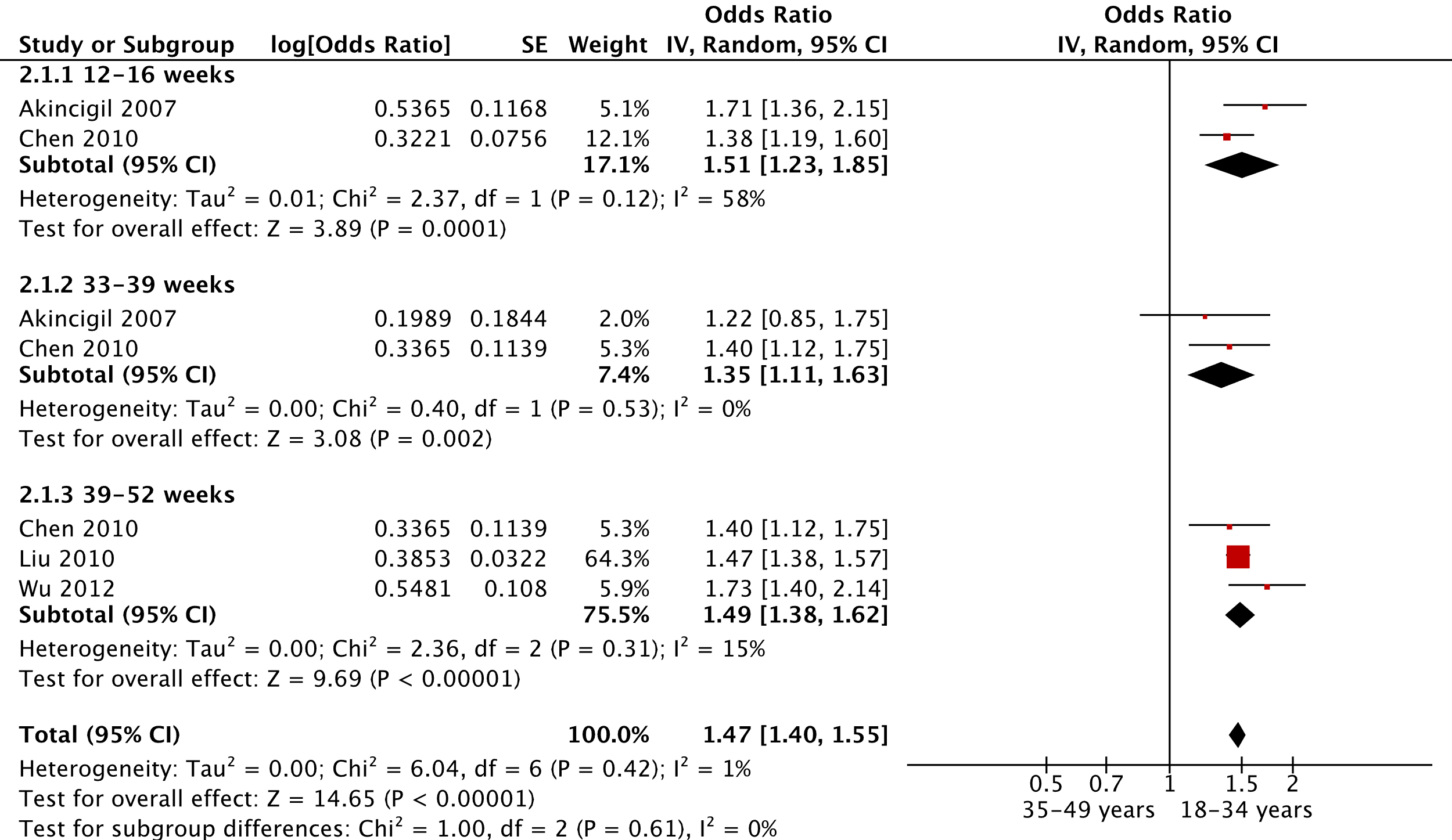


Supplementary Figure 3. Forest plot. Age, 50-65 year - Implementation; 16-39 weeks


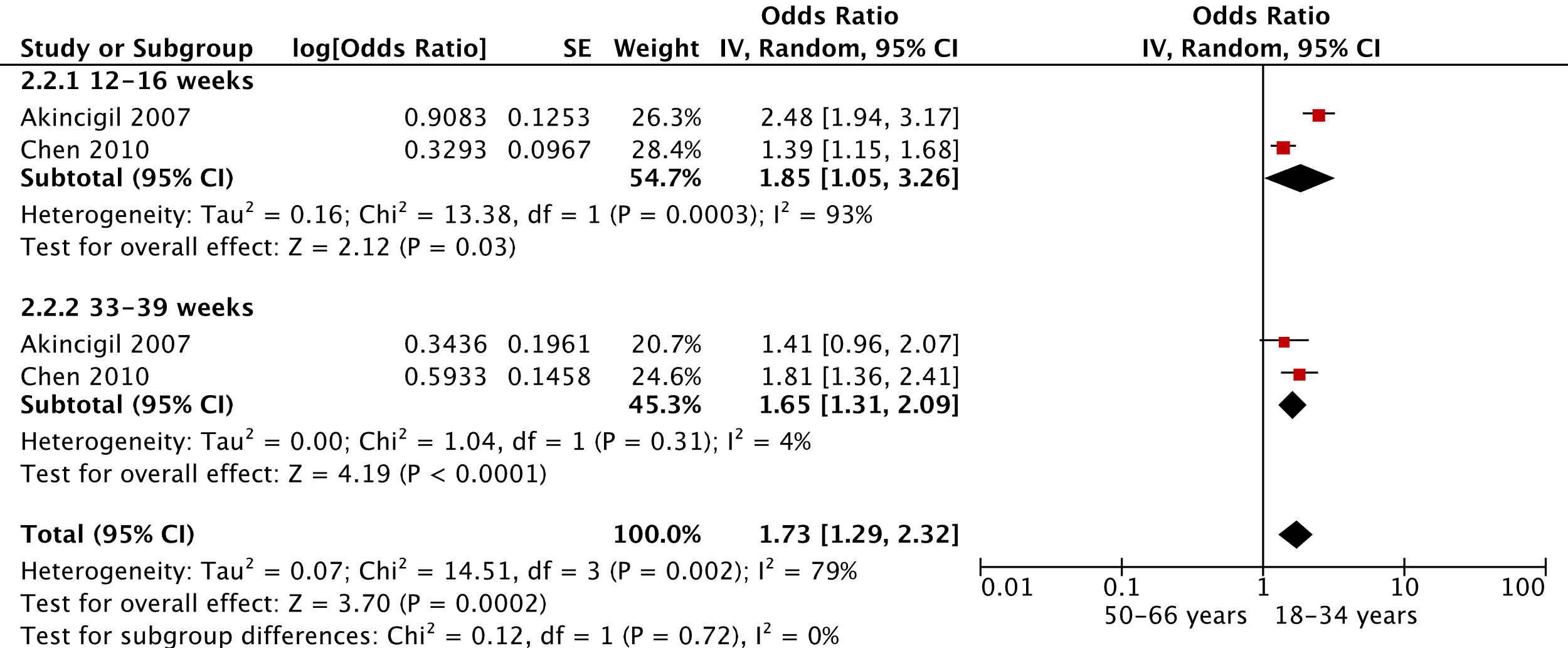


Supplementary Figure 4. Forest plot. Age, > 65 years – Implementation; 16-39 weeks


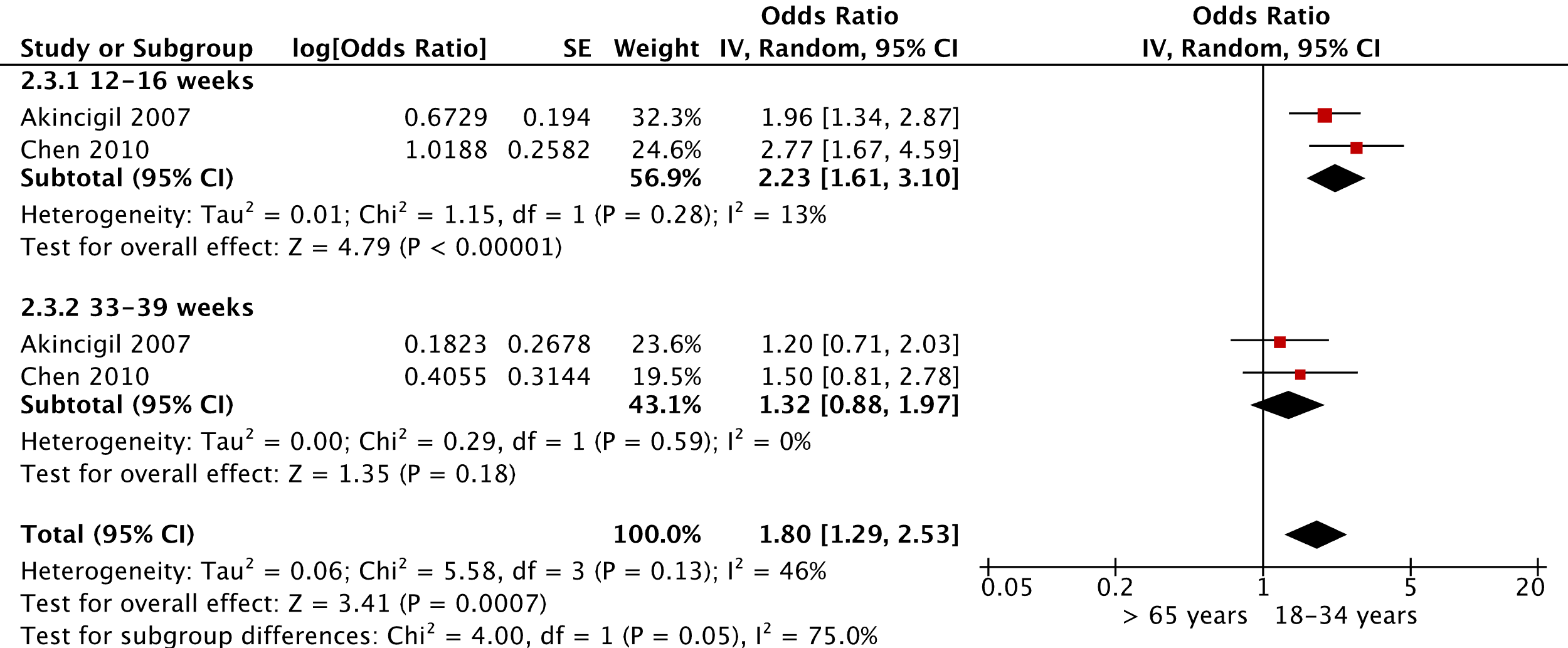


Supplementary Figure 5. Forest plot. Sex - Implementation; 16-52 weeks


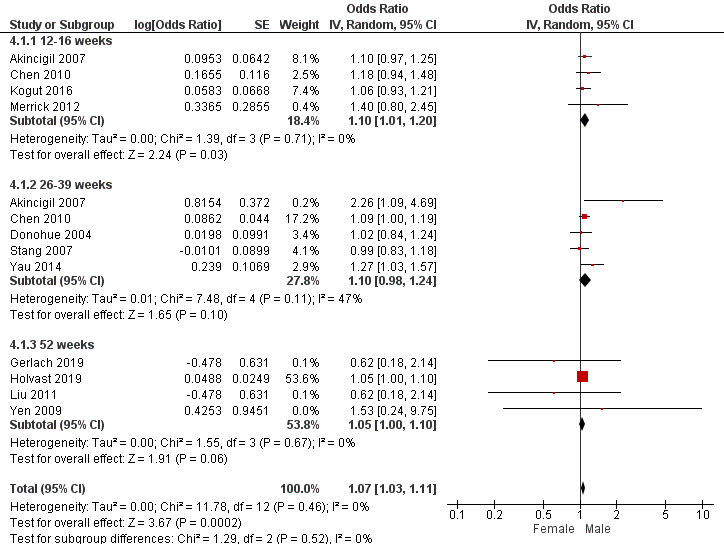


Supplementary Figure 6. Forest plot. Ethnicity – Implementation; 16-52 weeks


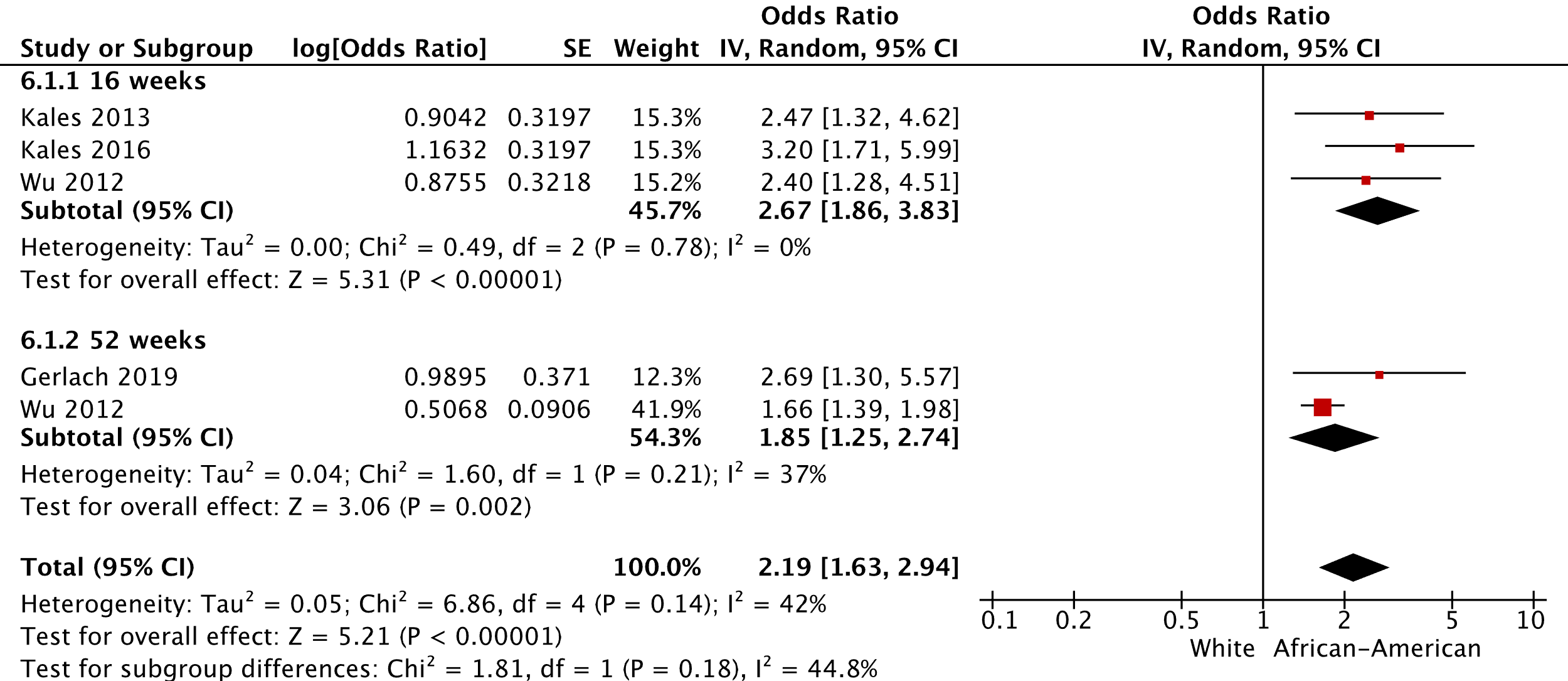


Supplementary Figure 7. Forest plot. Psychiatric comorbidity, Anxiety – Implementation; 16-39 weeks


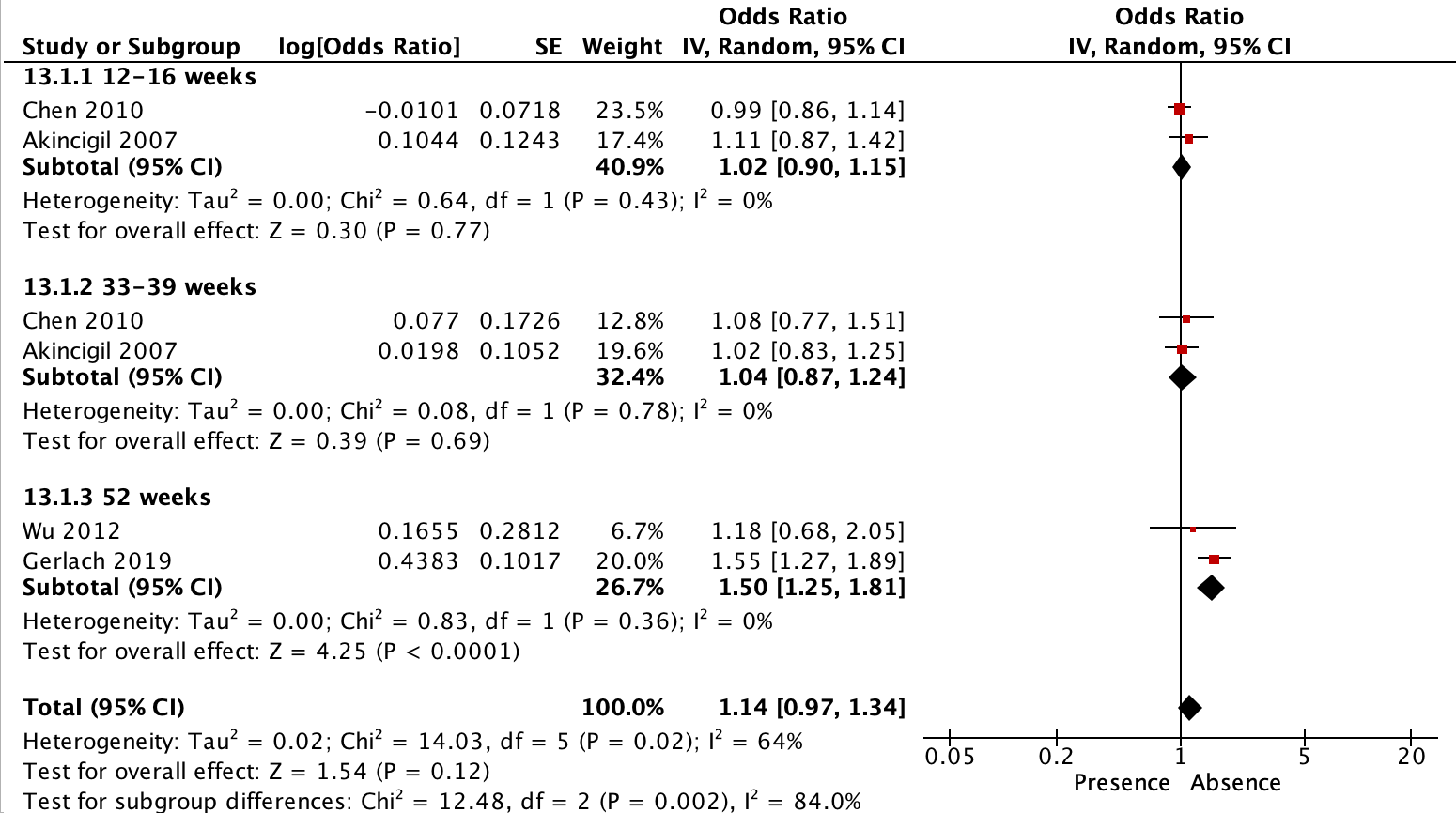


Supplementary Figure 8. Forest plot of comparison: Age - Discontinuation, 12 weeks (continuous).


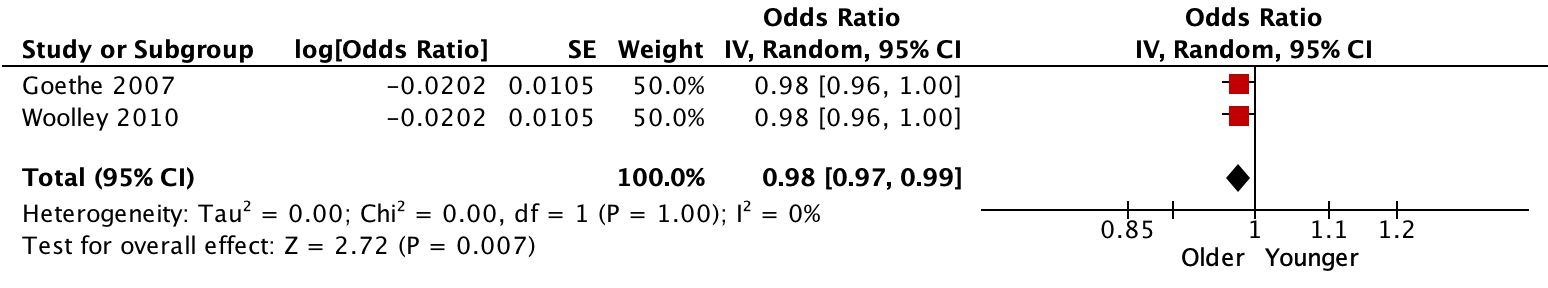


Supplementary Figure 9. Forest plot of comparison: Age, 25-40 years - Discontinuation; 26-52 weeks


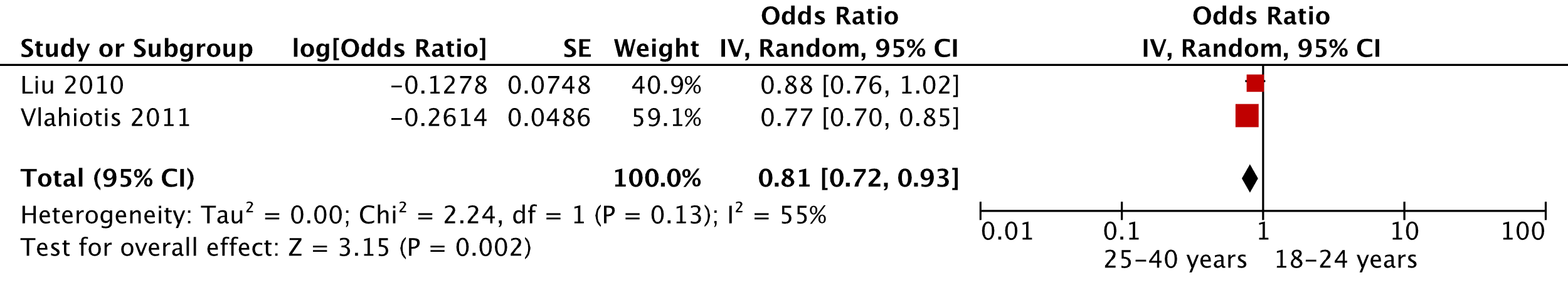


Supplementary Figure 10. Forest plot of comparison: Age, 56-64 years - Discontinuation; 26-52 weeks


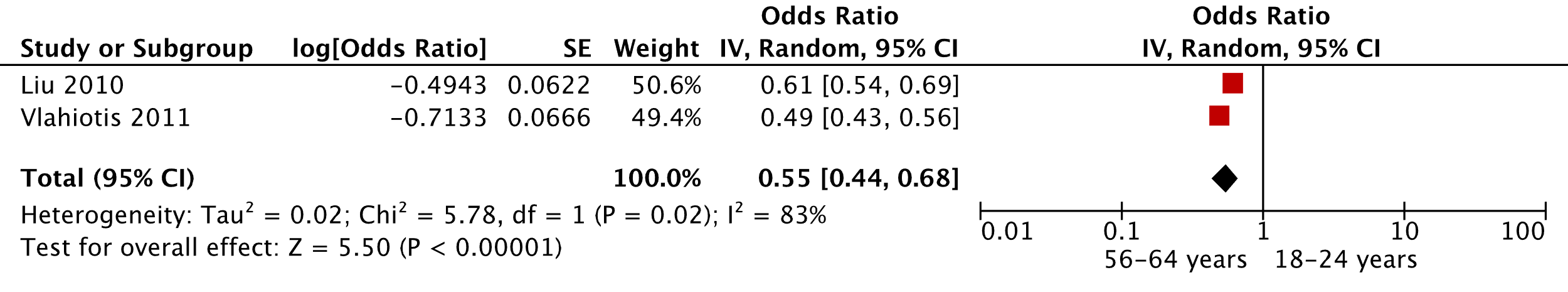


Supplementary Figure 11. Forest plot of comparison: Age, 40-65 years - Discontinuation; 52 weeks


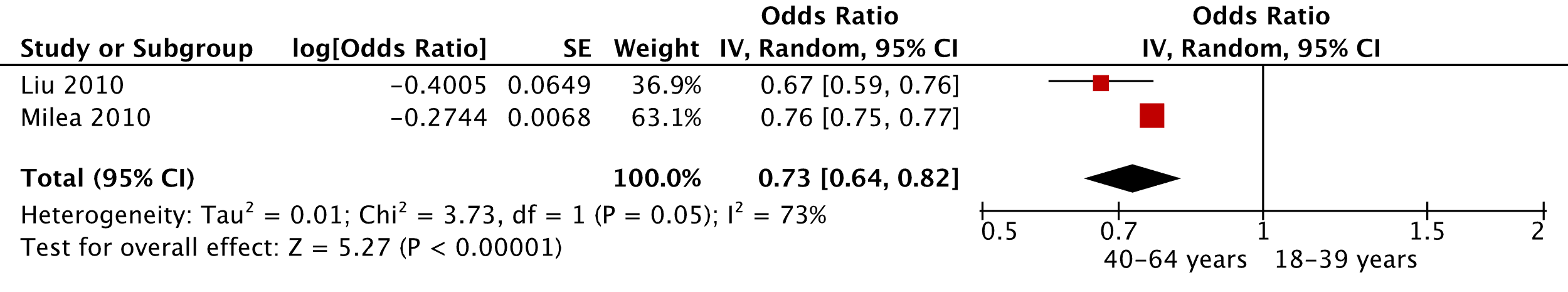


Supplementary Figure 12. Forest plot of comparison: Sex – Discontinuation; 4 weeks


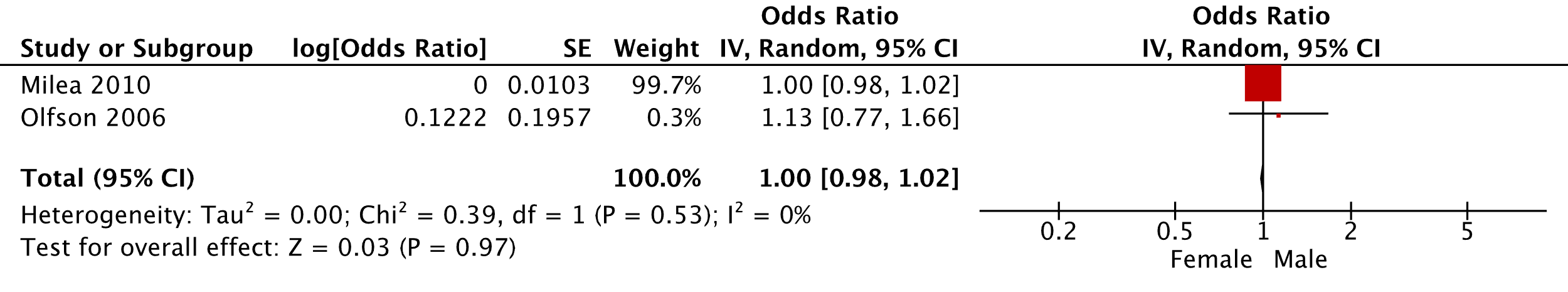


Supplementary Figure 13. Forest plot of comparison: Psychiatric comorbidity – Discontinuation; 26 weeks


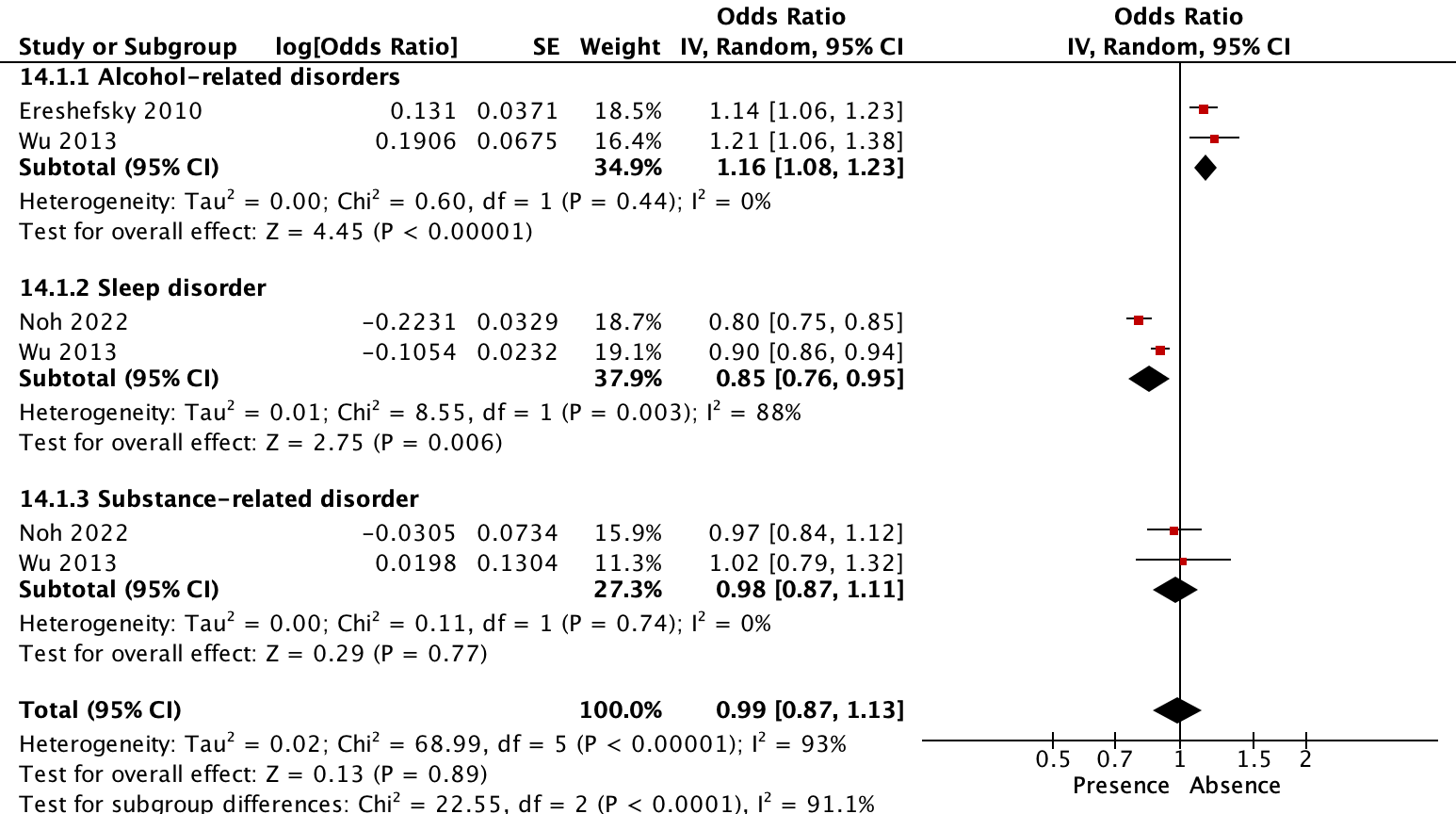

Supplement: Supplementary file 1 [file Table1.DOCX]
